# DNA damage-associated vesicle production in *Stenotrophomonas maltophilia* is mediated by the maltocin endolysin

**DOI:** 10.1128/jb.00158-26

**Published:** 2026-06-25

**Authors:** Darshan Chandramowli, Eva Mortier, Joni Elsmoortel, Jolien Vitse, Stijn De Waele, Stephan Stremersch, Koen Raemdonck, Kevin Braeckmans, Jan Felix, Bart Devreese

**Affiliations:** 1 Laboratory of Microbiology, Department of Biochemistry and Microbiology, Ghent University 366761, Ghent, Belgium; 2 Laboratory of General Biochemistry and Physical Pharmacy, Faculty of Pharmaceutical Sciences, Ghent University 82327, Ghent, Belgium; 3 Unit for Structural Biology, VIB-UGent Center for Inflammation Research and Department of Biochemistry and Microbiology, Ghent, Belgium; Dartmouth College Geisel School of Medicine, Hanover, New Hampshire, USA

**Keywords:** phage, antibiotic response, membrane vesicles

## Abstract

In this study, we examined the effects of antibiotic-mediated DNA damage on vesiculation in a clinical strain of *Stenotrophomonas maltophilia* (strain 44/98, also referred to as LMG 26284). Although genomic analysis of this strain revealed multiple prophage clusters, subsequent proteomic analysis showed that, among the proteins most significantly affected by ciprofloxacin exposure, the majority belonged to the previously described maltocin gene cluster. We showed that deletion of the gene coding for the maltocin endolysin (*mal*) reduces cell lysis and, in turn, the vesiculation capacity of *S. maltophilia*. Furthermore, using a combination of fluorescence and cryogenic electron microscopy, we provided evidence that the spontaneous re-arrangement of shattered membrane fragments attributable to *mal*-induced explosive cell lysis results in the formation of predominantly single-layered explosive outer membrane vesicles, while only a minor portion are double-layered outer-inner membrane vesicles. We also detected putative cytoplasmic vesicles in these samples, suggesting an expanded role of cell lysis in vesicle formation. Finally, we showed that the maltocin endolysin (LysSM) is upregulated rapidly in response to DNA damage stress and accumulates at the cell poles, which are likely the sites of subsequent cell lysis. Our findings expand on the involvement of (cryptic) tailocins in vesicle biogenesis under stress conditions.

**IMPORTANCE:**

In their natural environment, bacterial cells are able to communicate among themselves in multiple ways, and this communication has important implications for their ability to deal with unfavorable conditions. One key consequence is the spread of antimicrobial resistance from resistant cells to the remaining population, and such transfer can be mediated by membrane vesicles. In the highly multidrug-resistant bacterium *Stenotrophomonas maltophilia*, there exists a prophage that produces an endolysin capable of causing cell lysis and atypical vesicle production. The significance of this study lies in the description of such less characterized types of membrane vesicles produced in response to DNA damage, thereby providing insight into how bacteria respond to stress that can be brought about by antibiotic exposure.

## INTRODUCTION

Since the first observation of membrane vesicles (MVs) in a culture of *Escherichia coli* in the mid-1960s, several reports of MVs produced by other organisms have been published, leading to the hypothesis that vesiculation is a conserved process across all domains of life (1, 2). In the decades since, our knowledge about the different types of MVs and their functions has significantly improved—despite this, it has also been posited that our current perceptions severely underestimate their roles (3, 4). In Gram-negative bacteria, the most common type of MV results from the blebbing of the outer membrane, giving rise to outer membrane vesicles (OMVs). Various triggers can result in the production of OMVs, including oxidative stress and exposure to cell wall-destabilizing antibiotics like β-lactams (3). Interestingly, the cargo enveloped within OMVs has been shown to correlate with the trigger that stimulated its production—for example, OMVs produced by the opportunistic pathogen *Stenotrophomonas maltophilia* exposed to a sub-minimum inhibitory concentration (MIC) of benzylpenicillin were found to be enriched with two chromosomally encoded β-lactamases (L1 and L2), and administration of these vesicles to cultures of *Pseudomonas aeruginosa* and *Burkholderia cenocepacia* was demonstrated to protect against imipenem and ticarcillin treatment (5, 6).

One surprising observation from early experiments was the detection of genetic material packaged into MVs (7–9). The presence of these cytoplasmic contents could not be explained under the assumption that vesicles were produced exclusively from the outer membrane—as a result, it was theorized that these represented a new type of MV formed by the protrusion of the inner cell membrane into the periplasm, such that the resulting vesicle would have both outer and inner membranes. The first experimental evidence for this type of vesicle came from experiments conducted on *Shewanella vesiculosa*, where transmission electron microscope (TEM) images unequivocally revealed the presence of vesicles with a bilayered membrane—these were aptly called outer-inner membrane vesicles (O-IMVs or OIMVs) (10). Such vesicles have since been identified in multiple other pathogenic bacteria, including *P. aeruginosa* and *Acinetobacter baumannii* (11).

When faced with DNA damage, an important consequence is the activation of lysogenic bacteriophages, such as the λ phage in *E. coli* (12). This causes a transition to the lytic stage of their replication cycles, wherein the host bacterial cells are lysed to release multiple progeny viruses that can infect other cells. Recently, it was proposed that such an event (termed explosive cell lysis) was responsible for the production of OIMVs in *P. aeruginosa*. It was demonstrated that upregulation of the SOS response (a global mechanism in response to DNA damage) stimulated the production of a prophage endolysin (*lys*), which acts on the peptidoglycan layer of the cell wall, weakening it enough to cause cells to “explode,” with the spontaneous rearrangement of membrane fragments into OIMVs as a consequence (13). This pathway is now considered to be the major mechanism of OIMV biogenesis, although this is still debated by some as a “true” biogenesis route since it typically involves cell death (3).

Unsurprisingly, OIMVs have also been detected in culture supernatants of *S. maltophilia* exposed to ciprofloxacin, a fluoroquinolone that functions by inhibiting the actions of bacterial DNA gyrase and topoisomerases, resulting in single- and double-strand breaks (DSBs) in replicating DNA strands and subsequent cell death (14, 15). Akin to the more well-characterized *P. aeruginosa*, there exists a cryptic prophage within the genome of *S. maltophilia*, first described in the strain P28. The product of this gene cluster is a tailocin (more accurately, a phage tail-like bacteriocin) named maltocin, and it was found to show similarity to the organization of the rigid (R)-type pyocin gene cluster in *P. aeruginosa* (16). A more recent report of another maltocin was made in strain S16, with P28 and S16 gene clusters showing high degrees of similarity (17). One gene within this common maltocin operon (corresponding to the locus name *smlt1054* in the clinical type-strain K279a) is predicted to code for an endolysin that shows broad-spectrum bactericidal activity (18). We previously obtained preliminary indications that this endolysin, accompanied by a number of proteins derived from the maltocin gene cluster, is induced upon ciprofloxacin stress in a clinical isolate of *S. maltophilia* (strain 44/98, LMG 26824) (14). In this study, we work with the hypothesis that this endolysin (which we designate here as LysSM encoded by the gene *mal*) plays an analogous role to the *lys* endolysin in *P. aeruginosa* for the production of MVs in response to DNA damage. We confirmed massive induction of the maltocin gene cluster upon fluoroquinolone stress and report on the effects of antibiotic and genotoxic-mediated DNA damage stress on the membrane integrity by comparing the wild-type (WT) strain with a LysSM deletion mutant (Δ*mal*) using microscopy and other microbiological techniques.

## RESULTS

### Identification and analysis of prophage sequences in *S*. *maltophilia* strain 44/98

**Fig 1:**
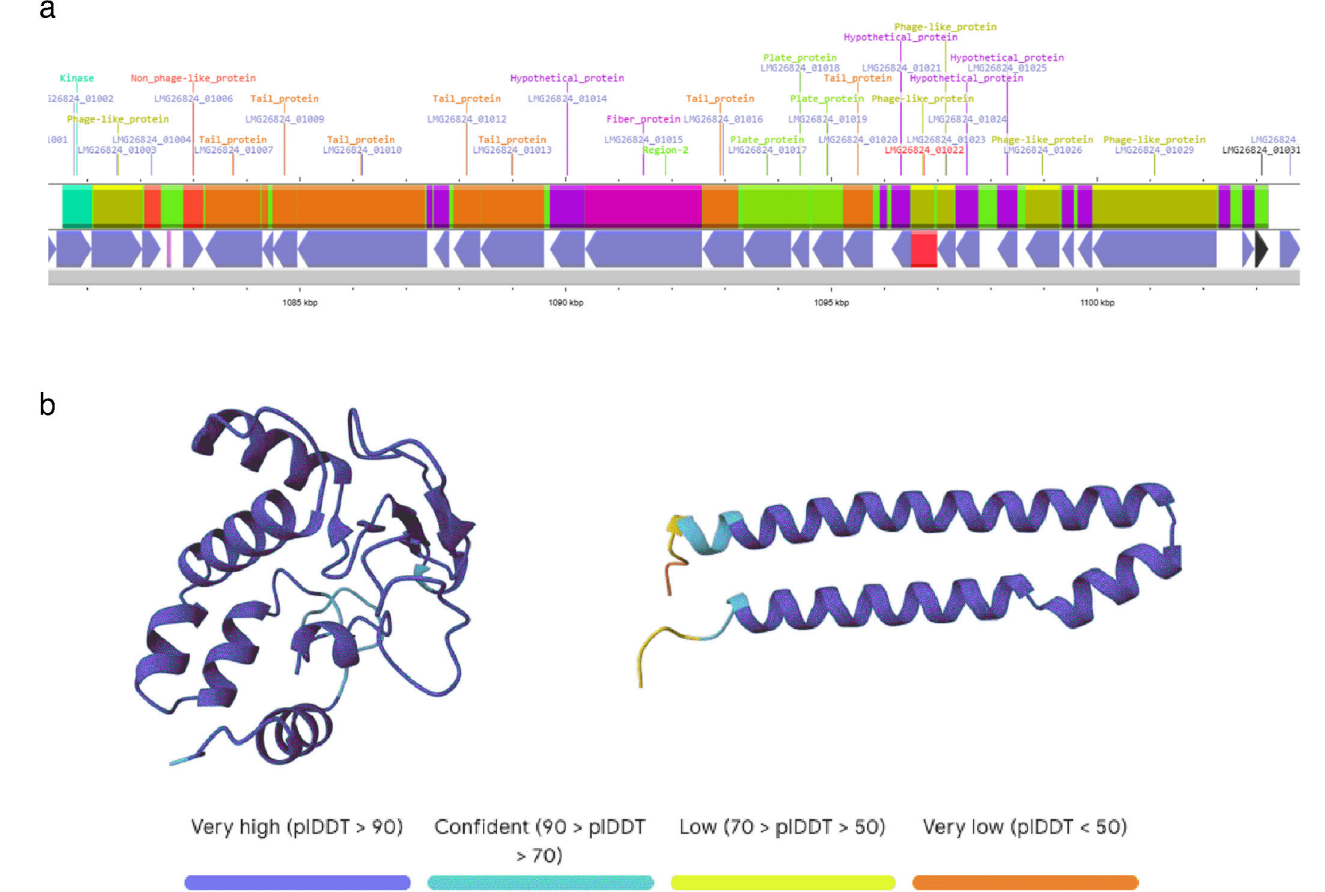
Analysis of the maltocin gene cluster present in *S. maltophilia*. (**a**) PHASTEST annotations of the various genes found within the predicted prophage region 2 tailocin cluster. The endolysin-coding gene (designated *mal* in this study) and the putative holin candidate are marked in red and black, respectively. (**b**) AlphaFold 3.0 structures of the LysSM endolysin (left) and the possible holin candidate (right).

The annotated genome sequence of *S. maltophilia* strain 44/98 is available at the European Nucleotide Archive (ENA) depository under the accession number GCA_977063385. The annotated genome was analyzed using PHASTEST, which identified a total of four prophage regions. All four are considered to be “intact,” although one of these regions (region 3, ORFs 02219–02230) is noticeably shorter than the remaining and lacks several phage-related genes (Material S1 and S2); therefore, region 3 is not further discussed. Among the remaining predicted regions, regions 1 (ORFs 00877–00934) and 4 (ORFs 02658–02724) appear to be proper prophage gene clusters (since structural genes corresponding to head, tail, and plate proteins, as well as key phage enzyme-coding genes like terminase and integrase, are present). Searching the proteins of region 1 against known proteins revealed similarity to the temperate bacteriophage S1, and the operon structure is well conserved (19). Based on a preliminary similarity search for gene products from region 4, this prophage was found to be related to bacteriophage CUB19 (20). Region 2 (ORFs 001002–001031) corresponds to a tailocin gene cluster (due to the absence of capsid-associated structural genes and typical phage-associated enzyme-coding genes) (Fig. 1). This gene cluster corresponds to the maltocin gene cluster originally discovered in strains P28 and S16, and it is virtually identical to the cluster annotated in the type-strain K279a (genes *smlt1035–1065*) with near 100% identity for the individual protein sequences (data not shown) (16, 17).

The PHASTEST gene annotations predicted a total of three phage-encoded lysozymes and endolysins, present in all but region 3.

1. The lysin identified within region 1 (ORF 00920) was found to be in close proximity to possible holin and spanin-coding genes (ORFs 00914 and 00917, respectively) (Material S2 and S3).
2. One of the genes within region 2 (ORF 01022) codes for the maltocin endolysin, which corresponds to the gene *smlt1054* in the type-strain K279a (99% sequence identity). The PHASTEST annotation suggested that the K279a endolysin had an extra 19 aa at the N-terminus; however, verification of the genomic data for strain 44/98 showed that these amino acids are indeed also present (Material S4). Furthermore, a possible holin-coding gene (ORF 01031) was also identified downstream (Fig. 1; Material S5).
3. The lysin annotated within region 4 (ORF 02692) was preceded by an annotated holin (ORF 02690) (Material S2 and S3). This lysin was annotated to be a member of the glycosyl hydrolase family 19 (GH19), whose members are known to exhibit chitinase activity (21). However, a subset of GH19 family members originates from bacteriophages, and it was suggested that these evolved to lysozyme-like enzymes. This was recently proven for two phage endolysins from an Antarctic *Pseudomonas* strain (22). This phage gene cluster was not found in the genome of the type-strain K279a. Furthermore, the annotated lysin shares high similarity (50% sequence identity) with the *lys* endolysin previously described in *P. aeruginosa* (Material S4) (13). The role of the homologous lysozyme within this region has previously been explored in the context of vesiculation in *P. aeruginosa* (13).

### The maltocin gene cluster is dramatically induced upon ciprofloxacin stress

Based on previous proteomic data, the gene products of region 2 (maltocin gene cluster) are markedly detected in *S. maltophilia* cultures treated with ciprofloxacin (14). However, since the data were only qualitative, we performed a quantitative proteomics analysis, where we compared the proteins produced by *S. maltophilia* in a ciprofloxacin-stressed culture versus an untreated control. The mass spectrometry data have been deposited to the ProteomeXchange Consortium via the PRoteomics IDEntification (PRIDE) partner repository (23) under the data set identifier PXD069601. This analysis revealed 188 proteins to be significantly different in abundance (minimum log_2_ fold-change >1), of which the majority showed increased abundance upon exposure to ciprofloxacin. Remarkably, 16 proteins in this list, some of them displaying the highest fold-change, are encoded by the maltocin gene cluster (Table S1, highlighted in yellow). The remaining phage clusters are only represented by two proteins with mild fold-changes (Table S1, highlighted in blue). Therefore, we focused our study on the role of the maltocin endolysin, which we henceforth designate LysSM (encoded by the gene *mal*).

### Construction of Δ*mal* mutants

**Fig 2:**
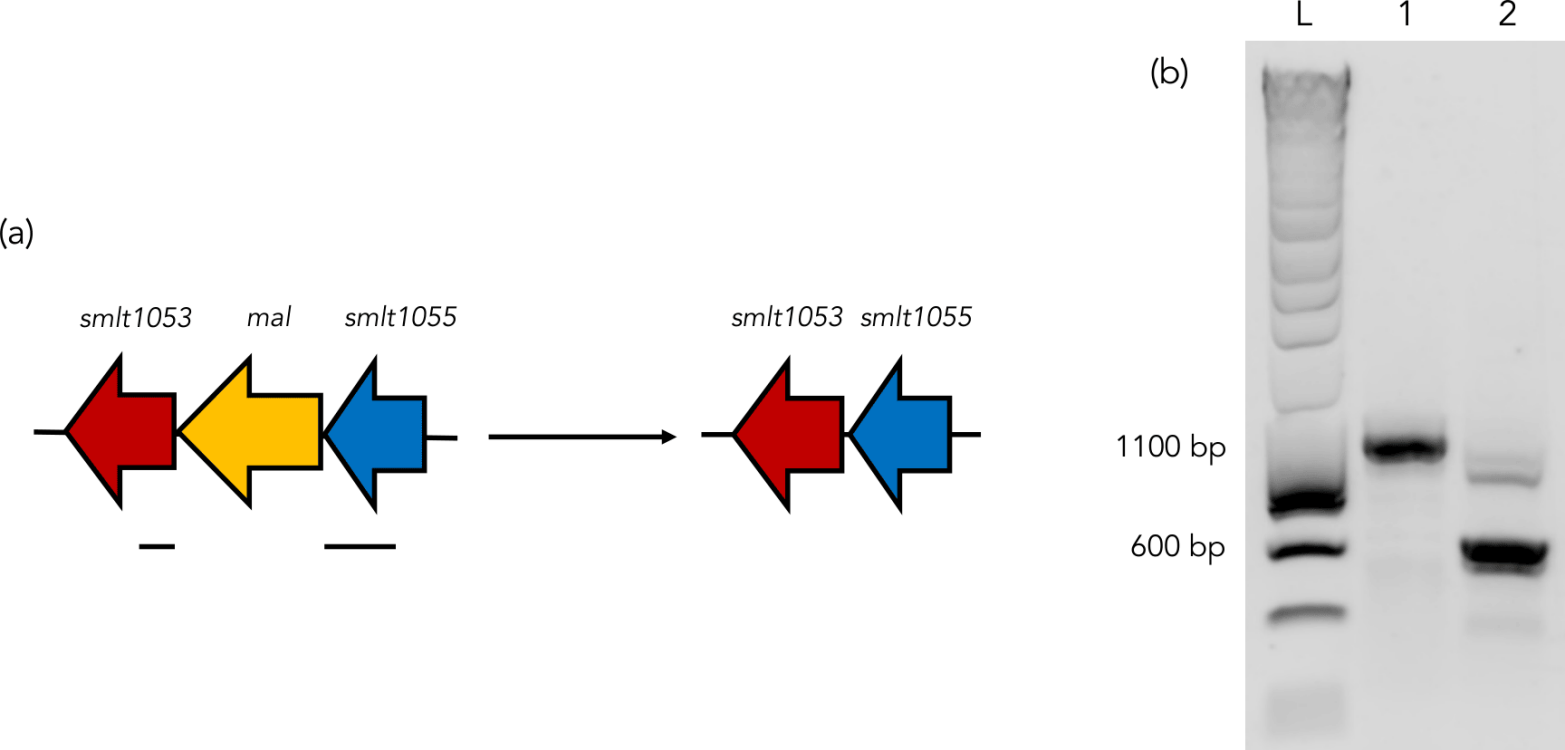
Deletion of *mal*. (**a**) Deletion schematic for Δ*mal*. The upstream and downstream homology regions used for the construction of the suicide plasmid are marked under the figure. (**b**) PCR confirmation of Δ*mal*. Lane L, ladder; lane 1, PCR of WT flanking regions of *mal*; lane 2, PCR of Δ*mal* flanking regions. The locus tags *smlt1053* and *smlt1055* refer to ORFs 01021 and 01023, respectively, in the genome of strain 44/98.

The Δ*mal* mutants were constructed using an allelic-exchange mutagenesis strategy optimized for poorly characterized multidrug-resistant bacteria (24). Of note, our approach was more successful when using homology regions significantly shorter (200–300 bp) than those typically reported in the literature thus far (500–1,500 bp) with comparable efficiency (Fig. 2) (25–27). This finding encourages future studies using this method to expend even less time by reducing the length of homology regions when constructing suicide plasmids, which is by far the most time-consuming and tedious part of the protocol.

### Deletion of *mal* appears to have a slightly positive impact on *S*. *maltophilia* growth but impairs biofilm formation and drastically affects vesiculation

**Fig 3:**
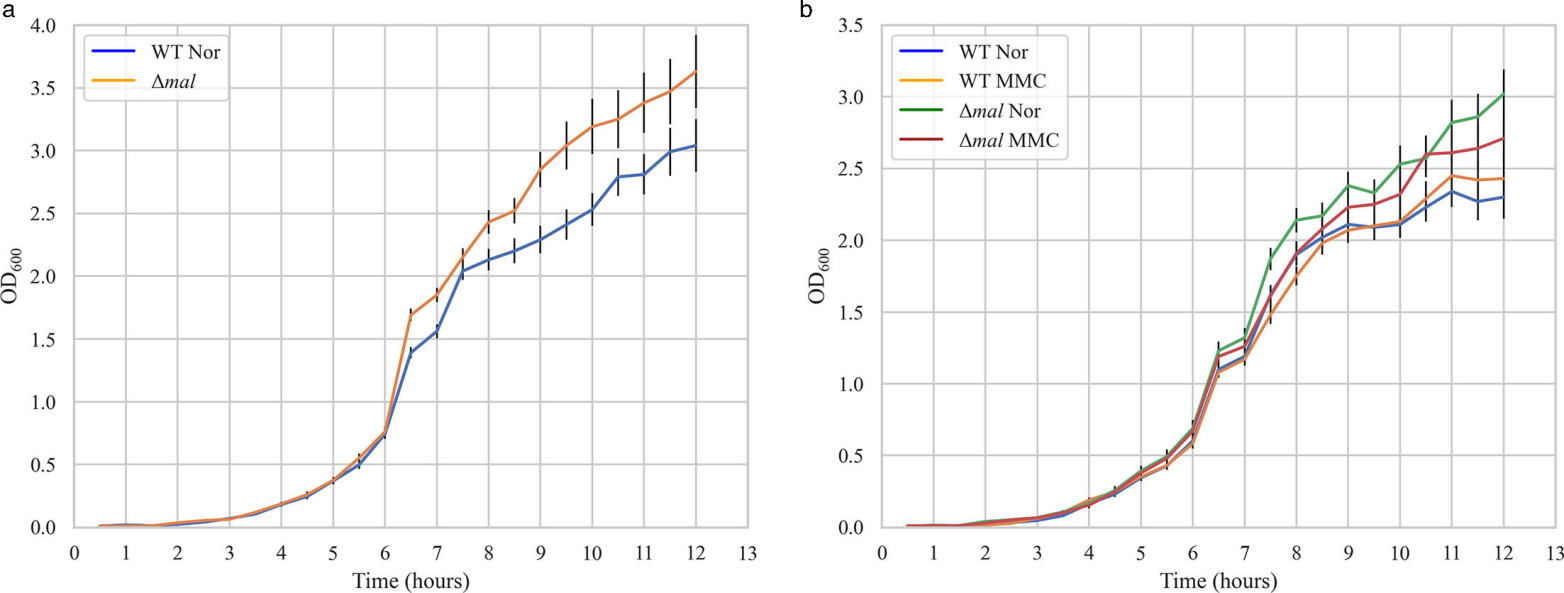
Growth curves of 44/98 WT and Δ*mal*. (**a**) Growth curves were constructed for cultures to which no antibiotics were added. (**b**) Growth curves were constructed for cultures to which sub-MICs of norfloxacin or mitomycin C were added. Antibiotics were added when culture densities reached 0.3–0.4. Nor, norfloxacin; MMC, mitomycin C.

To investigate the effect of the deletion of *mal* on the viability of *S. maltophilia*, growth curves were constructed for the WT and Δ*mal* strains. We observed that the deletion resulted in slightly improved growth under normal conditions, so growth curves were constructed for both strains that were exposed to sub-MICs of norfloxacin and mitomycin C at the start of the exponential phase. Here too, it was observed that growth of the Δ*mal* mutant exposed to the DNA-damaging compounds was better than the corresponding growth in the WT (Fig. 3). We rationalize these findings by correlating the absence of LysSM with decreased cell lysis and subsequent cell death relative to the WT, thereby improving cell growth.

**Fig 4:**
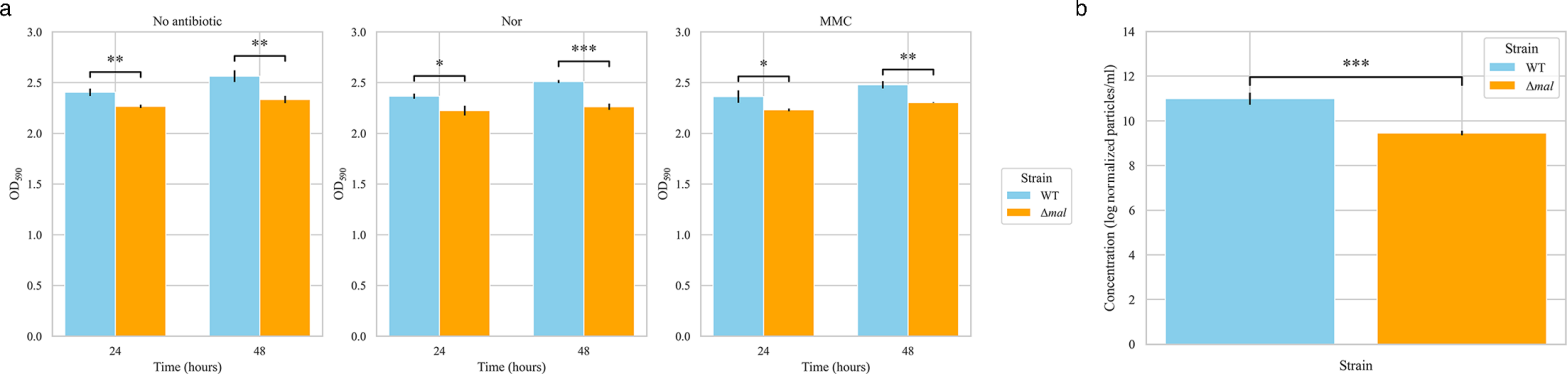
Effect of deletion of LysSM. (**a**) Comparison of biofilm formation between 44/98 WT and Δ*mal* in conditions of no antibiotic exposure, exposure to norfloxacin, and exposure to mitomycin C. Statistical significance was assessed using a t-test. Graph is shown as mean ± S.D. (**b**) Comparison of MV production between 44/98 WT and Δ*mal*. Plotted values have been log-transformed for the sake of convenience. Statistical significance was assessed using a t-test. Graph is shown as mean ± S.D.

**Fig 5:**
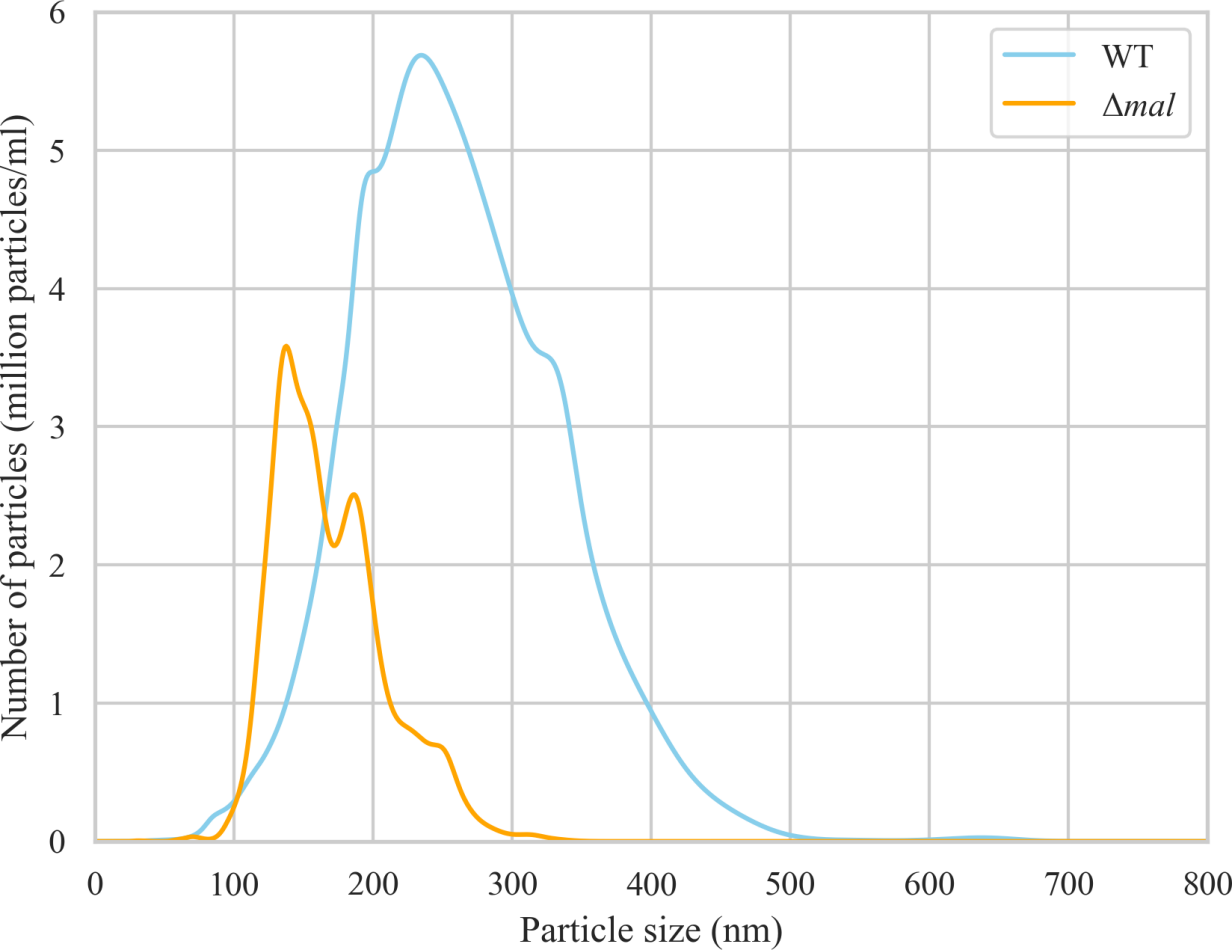
Size distribution of MVs produced by 44/98 WT and Δ*mal*. The average size of MVs produced by the WT is much larger than that of MVs isolated from the Δ*mal* mutant. Furthermore, the size range of WT vesicles (around 100–500 nm) is much more pronounced than that of Δ*mal* vesicles (around 100–300 nm).

To further test this hypothesis, we examined the effect of deletion of LysSM on biofilm-forming capability and MV production. Cell lysis results in the release of extracellular DNA and various cytoplasmic contents, which are known to stimulate biofilm formation and the production of different types of MVs, most notably OIMVs and explosive outer membrane vesicles (E-OMVs). Concurrent with our hypothesis, we observed a decrease in biofilm formation after 24 and 48 hours in the Δ*mal* mutant relative to the WT when no antibiotics were added to the growth medium, as well as in response to DNA-damaging agents. For all conditions, the decrease observed was significant (Fig. 4; Table 1). Furthermore, a significant decrease in MV formation was also observed between WT (1.16 × 10^11^ particles/mL) and Δ*mal* cultures (2.91 × 10^9^ particles/mL) (Fig. 4; Table 1). Taken altogether, these findings highlight the role of LysSM in phage endolysin-triggered cell death and consequent MV production. Based on the results of the SPT experiment, a size distribution graph was constructed for the vesicles isolated from the WT and Δ*mal* cultures after exposure to ciprofloxacin. The optimal size range of the instrument used is around 100–1,000 nm, so particles smaller than the lower limit are underrepresented. Based on the data, it was observed that the average size of MVs obtained from the WT cultures (267.7 nm) was significantly larger than that retrieved from the Δ*mal* cultures (169.24 nm). Furthermore, the size range of WT vesicles was far greater than that of the Δ*mal* vesicles (Fig. 5; Table 1). For both strains, the size of the resulting MVs was considerably larger than those previously shown to be formed in response to β-lactams like imipenem (14). This suggests that LysSM is involved in the production of MVs in *S. maltophilia* in response to DNA damage, and the MVs produced by this route are likely morphologically distinct from those produced in response to cell wall stress.

**TABLE 1:** Statistical analysis of the effect of deletion of LysSM

Condition	*P*-value^*a*^
Biofilm formation	
No antibiotic, 24 hours	0.0039**
No antibiotic, 48 hours	0.0042**
Norfloxacin, 24 hours	0.0107*
Norfloxacin, 48 hours	0.0002***
Mitomycin C, 24 hours	0.0225*
Mitomycin C, 48 hours	0.0012**
MV production	
WT vs. Δ*mal*	0.0007***
MV size	
WT vs. Δ*mal*	0.0001****

^*a*^ *P < 0.05; **P < 0.01; ***P < 0.001; ****P < 0.0001.

### LysSM is implicated in cell lysis and consequent MV production; however, single-layered MVs are the predominant type of MV formed

**Fig 6:**
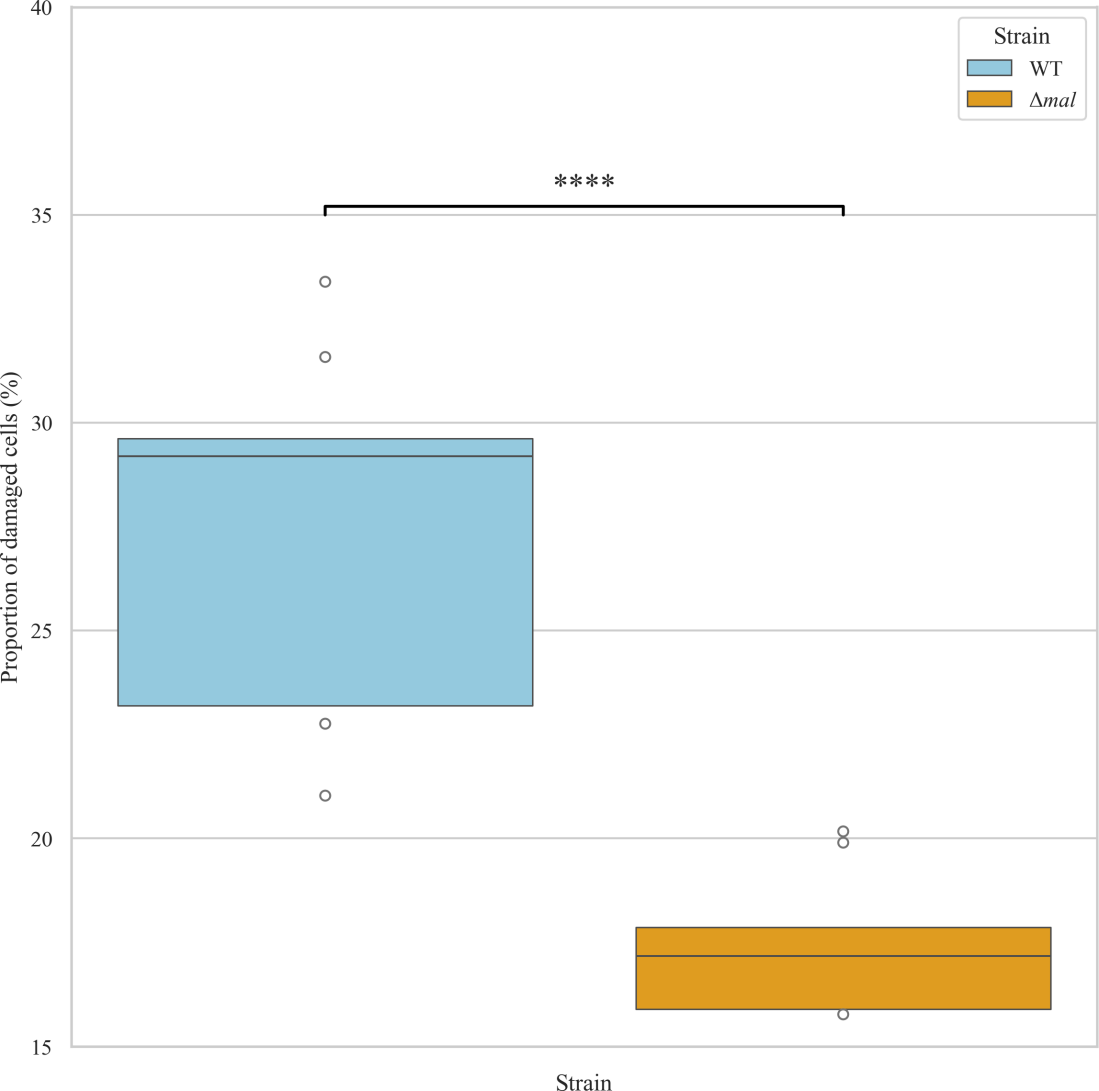
Bar plot displaying the proportions of *S. maltophilia* 44/98 WT and Δ*mal* cells in response to norfloxacin stress. The WT samples showed a significantly higher proportion of membrane-damaged (i.e., dead) cells relative to the Δ*mal* mutant (*P* < 0.0001) when analyzed using flow cytometry.

To assess the role of LysSM in cell lysis, a flow cytometry experiment was performed to compare the proportion of intact and damaged cells in exponentially dividing WT and Δ*mal* cells exposed to a sub-MIC of norfloxacin for 3 hours. While the average cell density was not significantly different (Table 2; Material S6), live/dead cell counting revealed that the Δ*mal* mutant populations had a significantly lower (*P* < 0.0001) percentage of cells with permeable membranes (Fig. 6). This clearly indicated that the LysSM endolysin is effectively engaged in cell lysis occurring after exposure to the fluoroquinolone.

**TABLE 2:** Average cell densities (per mL) of *S. maltophilia* 44/98 WT and Δ*mal* cultures as measured by flow cytometry

Biological replicate	WT	Δ*mal*
1	4.28E + 07	3.39E + 07
2	3.39E + 07	3.33E + 07
3	3.17E + 07	4.33E + 07
Average	3.61E + 07	3.68E + 07

To study the discrepancy in average MV size between the WT and Δ*mal* mutant, fluorophore-tagged mutants were constructed to try and visualize the effect of DNA stress on membrane integrity. For this purpose, fluorophore-tagged mutants were constructed for the WT and Δ*mal* mutant, wherein the outer membrane protein Ax21 was tagged with mCherry and the inner membrane ATP synthase γ subunit AtpG was tagged with eGFP—the resulting strains are referred to as DT (i.e., double-tagged) and DTΔ, respectively, in the subsequent sections.

**Fig 7:**
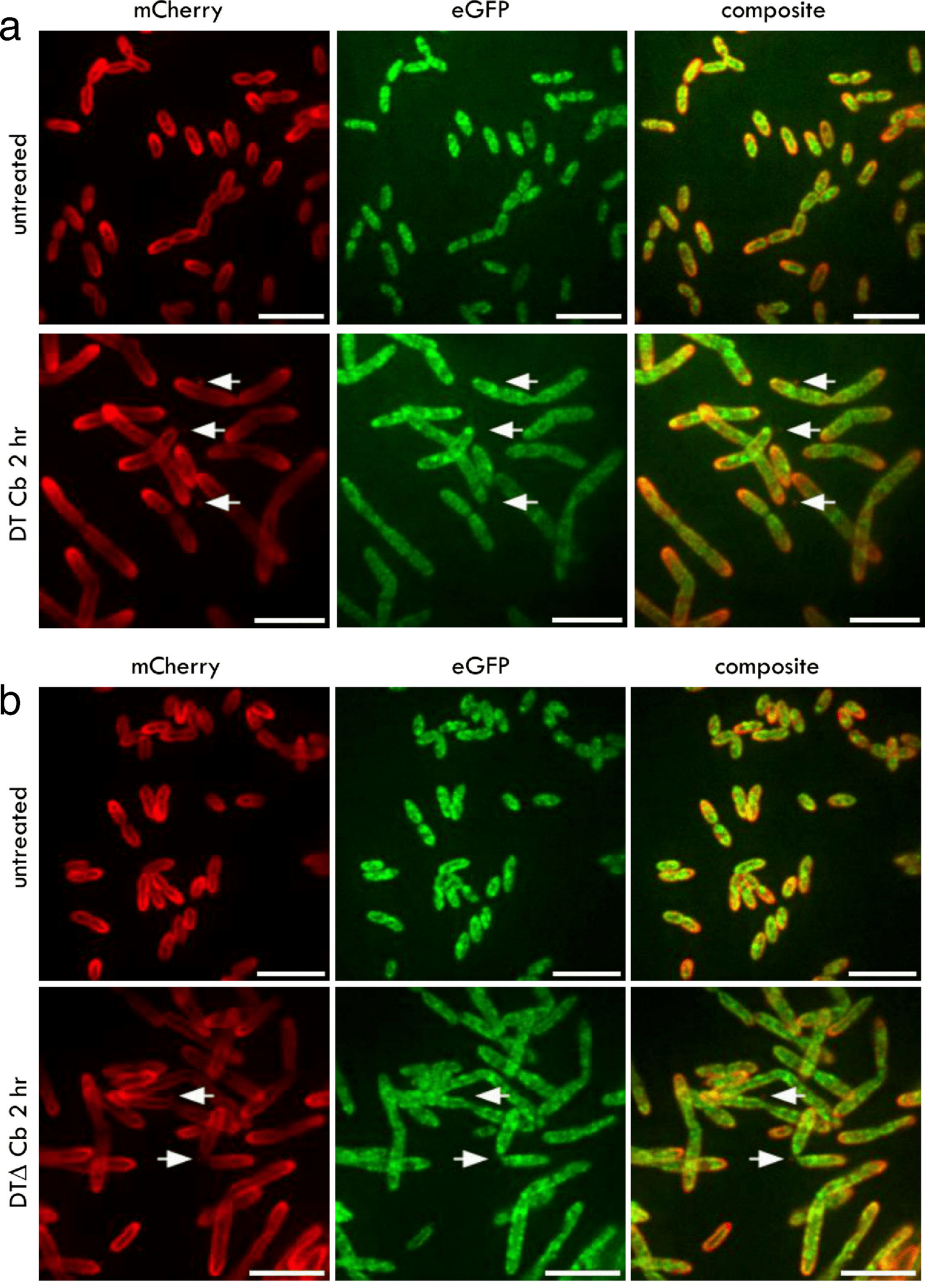
Effect of cell wall-targeting compounds on membrane integrity. (**a**) DT control and carbenicillin-treated cultures. Arrows show OMVs with mCherry signals visible and corresponding eGFP signals absent. (**b**) DTΔ control and carbenicillin-treated cultures. White arrows show OMVs with mCherry signals visible and corresponding eGFP signals absent. Re-localization of Ax21 (outer membrane) and AtpG (inner membrane) is visible in both sets of treated samples. Scale bar, 5 μm. Cb, carbenicillin.

As an initial test, exponentially dividing cultures of DT and DTΔ were subjected to carbenicillin for 2 hours. Exposure to such a cell wall-targeting antibiotic was anticipated to result in elongation of cells due to stalling of cell division and the production of mainly OMVs (28). Deletion of the maltocin endolysin should not have any effect on this type of MV production, since the tailocin is not induced by β-lactams (14). As expected, fluorescence microscopy revealed cell elongation and the presence of several spherical particles that showed signals from the mCherry but not the eGFP channel in both carbenicillin-treated samples—these were presumed to be OMVs (Fig. 7). Curiously, it was observed that AtpG became localized at specific well-defined foci throughout cells in treated samples compared to a more uniform distribution at the cell boundaries in the corresponding untreated controls—to a lesser extent, Ax21 also appeared to concentrate at the polar regions of many cells (Fig. 7; Material S7). Spatiotemporal changes in protein localization are considered good indicators of stress in cells; therefore, we were confident that our mutants could be used to assess membrane integrity (29).

**Fig 8:**
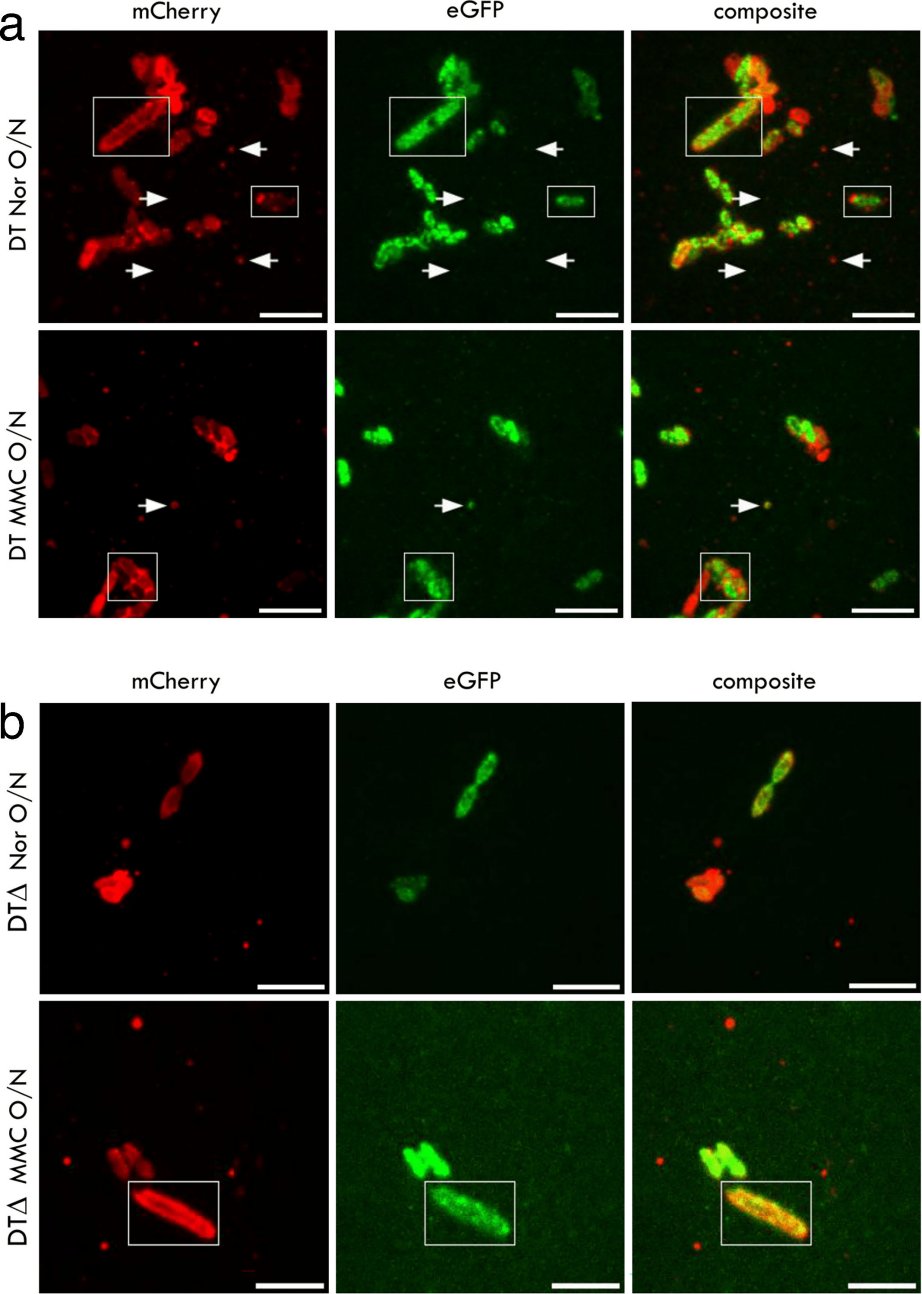
Effect of DNA damage-inducing compounds on membrane integrity after overnight exposure. (**a**) DT norfloxacin- and mitomycin C-treated cultures. (**b**) DTΔ norfloxacin- and mitomycin C-treated cultures. White boxes show cells with bright foci at cell peripheries, indicating membrane damage, and white arrows show bright spots corresponding to MVs or membrane fragments. Scale bar, 5 μm. Nor, norfloxacin; MMC, mitomycin C; O/N, overnight.

We then sought to gauge the severity of the effect of DNA damage by exposing DT and DTΔ cultures to sub-MICs of either norfloxacin or mitomycin C overnight. After 24 hours, fluorescence microscopy revealed cells with numerous distinct bright foci at the cell peripheries along with several bright spots separate from cells and distinct from background fluorescence, likely corresponding to both outer and inner membrane fragments. Analysis of the samples revealed that spots were more abundant in DT samples when compared to DTΔ samples, and overlapping of mCherry and eGFP signals was more accurately detected in DT samples (Fig. 8; Material S7). While the data cannot be considered to be truly quantitative, they appear to suggest that more cell lysis occurs in the WT relative to the Δ*mal* strain in response to DNA damage. When the experiment was repeated with only 2 hours of exposure to norfloxacin or mitomycin C, similar evidence of cell lysis was not seen; however, the localization of the inner membrane protein AtpG was found to be affected, becoming more diffuse in nature (Material S7). We hypothesize that the spots with coinciding fluorescence signals likely correspond to bilayered OIMVs formed by spontaneous re-circularization of membrane fragments after LysSM-induced cell lysis, thus explaining why they were detected less frequently in DTΔ samples.

**Fig 9:**
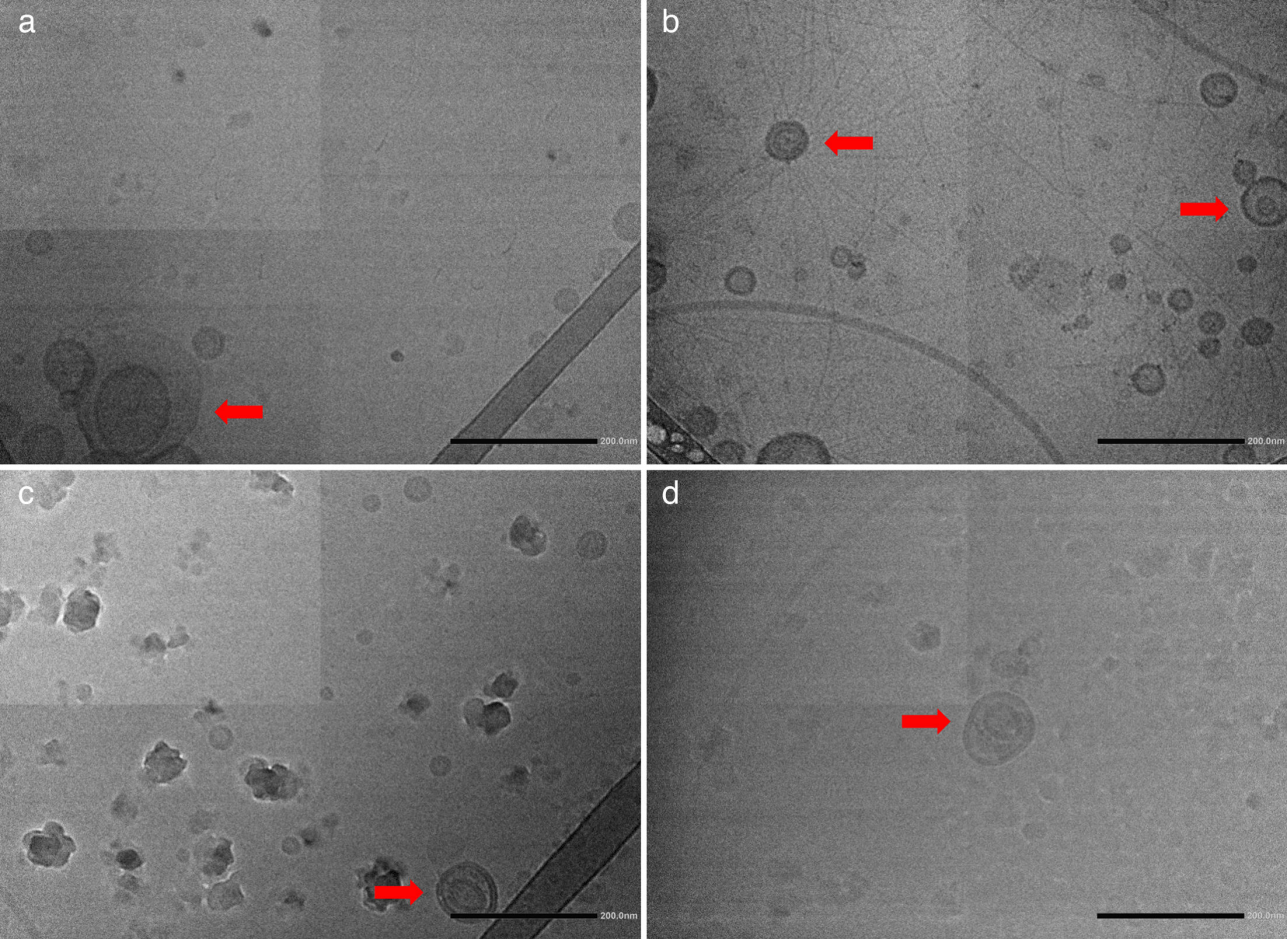
Cryo EM images of *S. maltophilia* 44/98 cultures exposed to DNA-damaging agents showing OIMVs. (**a**) General field of view of the WT culture treated with norfloxacin. A rather large OIMV is clearly visible (red arrow). (**b**) General field of view of the WT culture treated with mitomycin C. Two OIMVs are clearly visible (red arrows). The OIMV on the left appears to have several filamentous particles (possible phage particles) adherent to its surface. (**c** and **d**) General fields of view of Δ*mal* cultures treated with norfloxacin and mitomycin C, respectively. Not many vesicles are visible, although OIMVs are still occasionally visible (red arrows). Scale bar, 200 nm.

**Fig 10:**
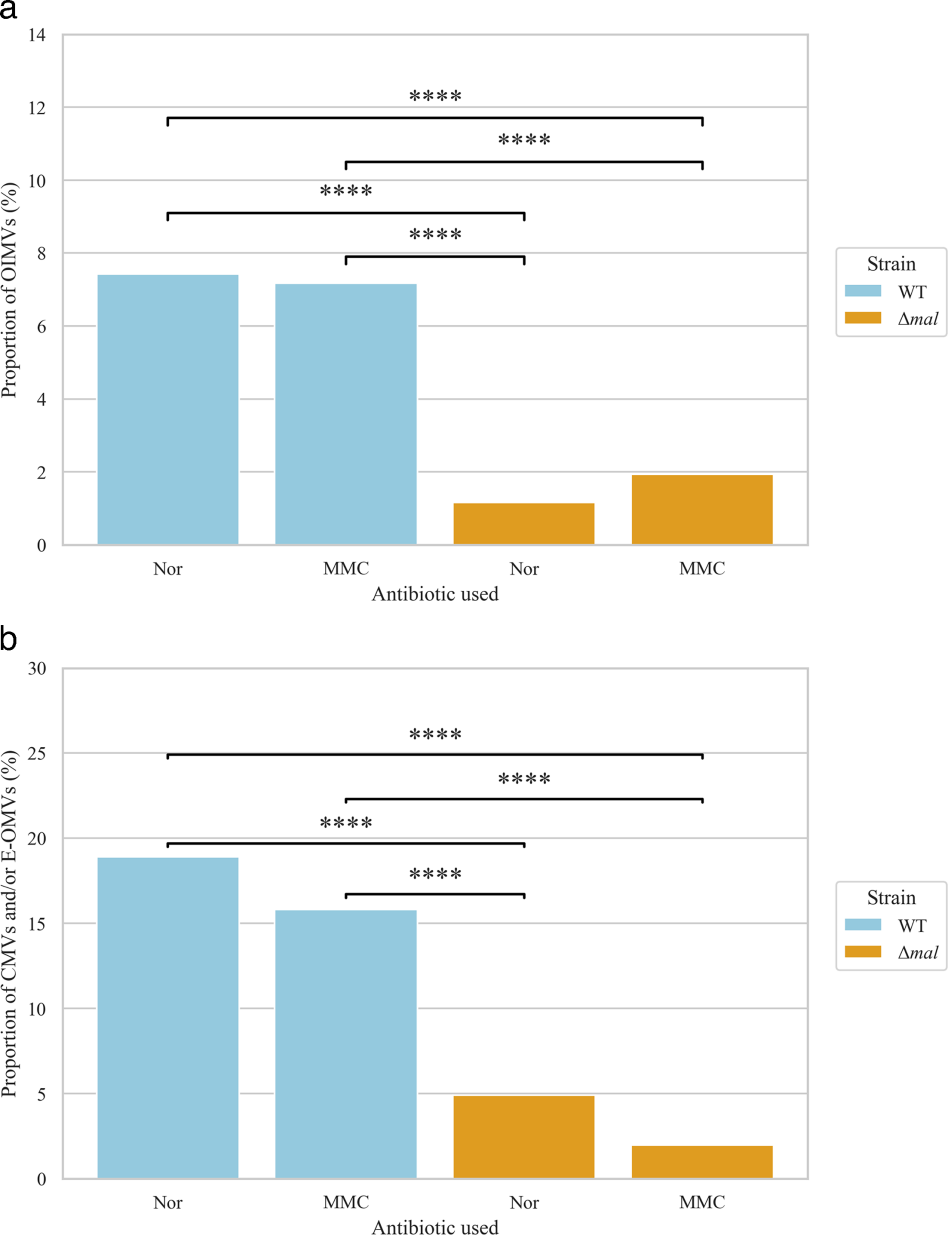
Proportions of different sub-populations of MVs in 44/98 WT and Δ*mal* samples. (**a**) The proportions of OIMVs in WT and Δ*mal* samples treated with norfloxacin and mitomycin C. (**b**) The estimated proportions of putative CMVs and/or E-OMVs in WT and Δ*mal* samples treated with norfloxacin and mitomycin C. Statistical significance was assessed using a chi-square test of independence, followed by post hoc pairwise comparisons using Z-tests and a Bonferroni correction.

**Fig 11:**
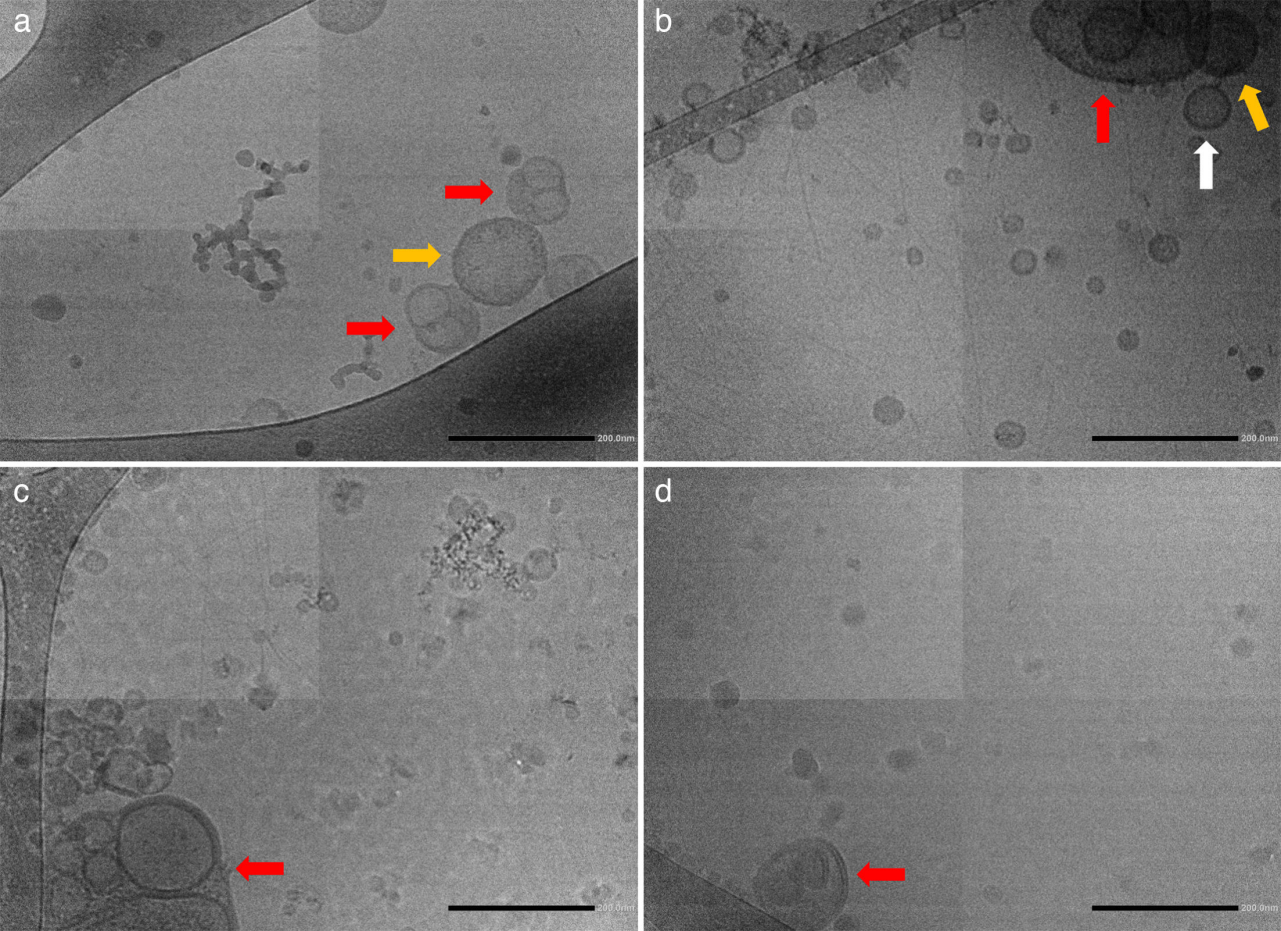
Cryo EM images of *S. maltophilia* 44/98 cultures exposed to DNA-damaging agents showing atypical OIMVs. (**a**) WT culture treated with norfloxacin. Two OIMVs with multiple cytoplasmic compartments (red arrows) are marked, present on either side of what appears to be a putative CMV or an E-OMV (orange arrow). (**b**) WT culture treated with mitomycin C. An OIMV with two cytoplasmic compartments (red arrow) is in close proximity to a regular OMV (white arrow) and a putative CMV or E-OMV (orange arrow). (**c**) WT culture treated with mitomycin C. An extremely large OIMV is clearly visible with at least six cytoplasmic compartments (red arrow). (**d**) Δ*mal* culture treated with mitomycin C. An OIMV with two cytoplasmic compartments is visible (red arrow). Scale bar, 200 nm.

**Fig 12:**
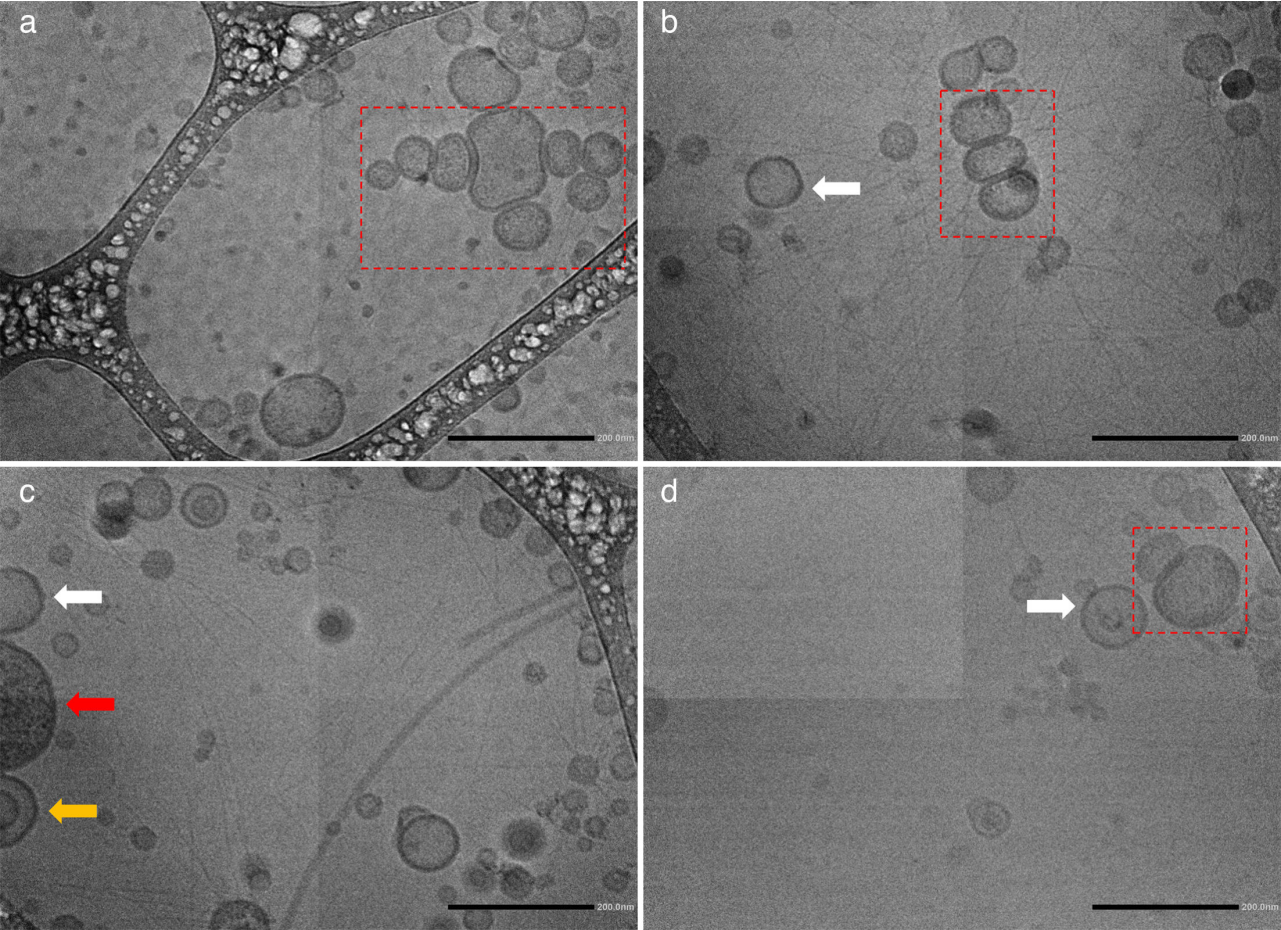
Cryo EM images of *S. maltophilia* 44/98 cultures exposed to DNA-damaging agents showing putative CMVs or E-OMVs. (**a** and **b**) WT culture treated with norfloxacin. Multiple vesicles with granular electron-dense cargo are visible (red boxes) as compared to a regular OMV (white arrow). These vesicles tend to be found in close proximity to each other. (**c**) WT culture treated with mitomycin C. A regular OMV (white arrow), an OIMV (orange arrow), and a putative CMV or E-OMV (red arrow) are clearly visible. (**d**) WT culture treated with mitomycin C. A clump of two single-layered vesicles with granular cargos are seen (red box) next to a single-layered vesicle with periplasmic content (white arrow). Scale bar, 200 nm.

Cryo EM images reveal that the most common type of MVs in all samples are single-layered vesicles, with only a few double-layered vesicles visible across several fields of view (Fig. 9; Material S8). Noticeably, the size of most MVs in both sets of samples is rather small (around 20–50 nm), which is in apparent contradiction to the data obtained from the SPT data; however, this was expected given the optimal size range of the instrument used for measurements. The small diameters of the MVs observed are likely due to overnight exposure to the antibiotics used, resulting in increased cell lysis and formation of small MVs. The proportion of bilayered OIMVs is significantly higher in the WT samples treated with norfloxacin and mitomycin C when compared to the corresponding Δ*mal* samples, although there are unequivocally still some OIMVs found in the latter (Fig. 10; Table 3). While most OIMVs conformed to the archetypal description (a vesicle within a vesicle, both of roughly concentric size), we also observed numerous instances of peculiar OIMV morphologies (multiple vesicles enclosed within a larger vesicle) (Fig. 11; Material S8). Furthermore, we also noticed an appreciable number of single-layered vesicles that enclose matter that stains similarly to that observed in the inner compartments of OIMVs (i.e., electron-dense cargo). Such MVs were predominantly found in the WT samples, and in most cases, they appeared in close proximity to OIMVs, usually in clumps of 2–4 vesicles (Fig. 12; Material S8). From previous reports, it was concluded that MVs that show such darker-stained interiors contained cytoplasmic matter (10, 11, 30). Therefore, we speculate that these darkly stained MVs might be putative cytoplasmic membrane vesicles (CMVs)—due to the inherent randomness associated with their formation, it is also likely that some of these might also be E-OMVs that encapsulate some cytoplasmic cargo, although the distinction cannot be made simply based on their morphology. In any case, according to our estimates, such MVs occur at a much higher incidence in WT samples relative to the Δ*mal* samples (Fig. 10; Table 3).

**TABLE 3:** Statistical analysis of the difference in MV sub-population proportions

Condition	*P*-value
OIMV proportion	
WT treated with norfloxacin	<0.0001
WT treated with mitomycin C	<0.0001
Δ*mal* treated with norfloxacin	<0.0001
Δ*mal* treated with mitomycin C	<0.0001
Putative CMV/E-OMV proportion	
WT treated with norfloxacin	<0.0001
WT treated with mitomycin C	<0.0001
Δ*mal* treated with norfloxacin	<0.0001
Δ*mal* treated with mitomycin C	<0.0001

### LysSM is rapidly upregulated in response to DNA damage stress and re-localizes to the cell poles

**Fig 13:**
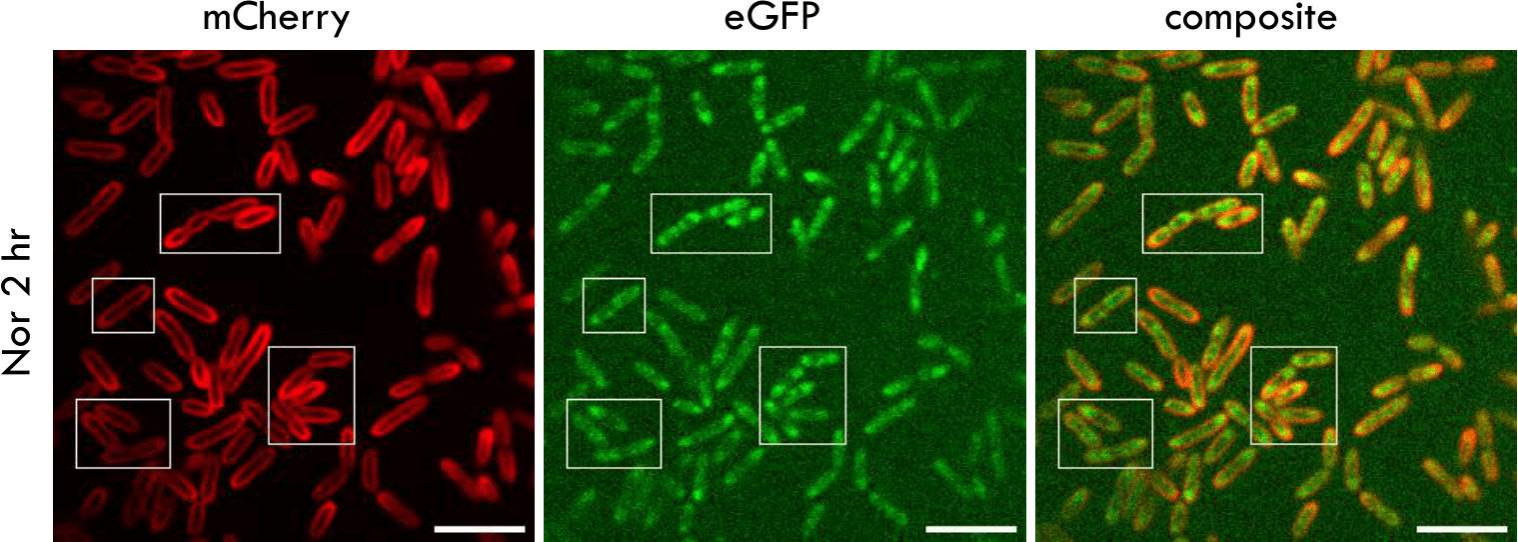
Effect of a sub-MIC of norfloxacin on membrane integrity after 2 hours of exposure. The *ax21:mCherry mal:egfp* mutant was exposed to 2 μg/mL norfloxacin for 2 hours to observe the upregulation of *mal*. Boxes show cells with a typical polar localization of LysSM (eGFP), while the outer membrane (mCherry) remains unaffected. Scale bar, 5 μm. Nor, norfloxacin.

Finally, we explored the spatiotemporal dynamics of LysSM in cells as a response to antibiotic-induced DNA damage—to this end, a standalone eGFP-tagged LysSM mutant and an mCherry-tagged Ax21 double mutant were constructed. A sub-MIC of norfloxacin was added to an exponentially growing culture of the *ax21:mCherry mal:egfp* mutant, and the culture was allowed to grow for an additional 2 hours. Fluorescence microscopy showed little-to-no change in outer membrane stability (based on Ax21 localization); however, LysSM was observed to be present as bright foci at the poles of most cells. At this time point, an excess of MVs was not noted in any of the fields of view imaged (Fig. 13). Wanting to visualize the real-time upregulation of *mal*, we then subjected the *mal:egfp* mutant culture to a high concentration of norfloxacin (10 μg/mL) and imaged cells immediately after and up to a duration of 1 hour post-exposure using time-lapse microscopy. During the initial minutes, little signal was observed; however, after approximately 15 minutes, several transient but non-stochastic spots were visible in multiple cells, similar to those observed after extended growth in the presence of the lower concentration of norfloxacin. In most cases, multiple foci were observed per cell, and they tended to appear most prominent toward the poles (Movies S1 and S2). We hypothesize that these correspond to future explosive lysis events, the products of which are a heterogeneous mixture of OIMVs, E-OMVs, and possibly CMVs.

## DISCUSSION

### Role of latent bacteriophages in vesiculation

In prior research, it was observed that treatment of *S. maltophilia* with ciprofloxacin similarly resulted in the production of OIMVs, along with several phage tail-like particles (i.e., tailocins) (14). Proteomic analysis confirmed a massive increase in proteins encoded by a cryptic prophage gene cluster, encoding the maltocin tailocin. Here, we assessed the role of the endolysin encoded by the maltocin gene cluster in the context of membrane integrity and vesiculation capacity. A decrease in cell lysis was observed in the LysSM-deficient mutant relative to the WT; however, a complete absence of cell lysis was not noted, and OIMVs were still detected in these cultures, indicating that there possibly exists some other trigger for their production. In this regard, it is possible that one or more of the other identified prophage endolysins play a (small) role, and this could form the basis for subsequent studies. The deletion of *mal* was not complemented using an expression plasmid, since genetic manipulation of *S. maltophilia* is notoriously difficult—furthermore, the selective pressure needed to maintain such a plasmid in the Δ*mal* mutant (likely using an antibiotic) would also constitute a state of stress that might confound conclusions. This somewhat limits the establishment of a concrete causal link between the deletion of *mal* and cell lysis, although we present ample evidence to support our claims.

A previous study in *P. aeruginosa* linked decreased MV production to various pyocin deletions (31). Following this, we demonstrated that not only is MV production in response to DNA damage impaired in the Δ*mal* mutant, but also the size of MVs is significantly reduced. The larger MVs secreted by the WT were previously thought to predominantly be OIMVs; however, using fluorescence microscopy, we observed little overlap between signals corresponding to outer and inner membrane fragments. As a result, we concluded that most single-layered MVs produced through LysSM-mediated explosive cell lysis are E-OMVs, a distinct type of OMV formed by the re-arrangement of outer membrane fragments—in additional support of this notion, it has also been proposed that unstable OIMVs can re-arrange to produce “second-generation” E-OMVs (3). The pyocin cluster in *P. aeruginosa* was found to also code for a holin (locus tag *PA0614*) that was observed to increase cell lysis by translocation of the *lys* endolysin to the peptidoglycan layer (13). A definitively annotated holin in the maltocin gene cluster was not found in the genome of strain 44/98, nor in the reference strain K279a; however, holins form a diverse group of proteins, so this is not surprising (32). Genome mining yielded a potential candidate (ORF 01031) that might fulfill this role, although further research is necessary to elucidate the roles of the genes that form these tailocin clusters.

### Qualification and quantification of atypical MVs linked to endolysin-mediated cell lysis

Data from our fluorescence microscopy experiments appear to suggest that the re-arrangement of inner membrane fragments also occurs, resulting in the production of CMVs—this also seems to be corroborated by data from cryo EM images that show single-layered vesicles with distinct darker staining profiles. CMVs have been reported in gram-negative bacteria, but their biogenesis remains unclear (3). It is not possible to provide a definitive answer about the presence of CMVs as yet, since MV sub-populations can neither be easily distinguished nor separated using current technology. Using metal nanoparticles that are specific to each membrane in conjunction with cryo EM is one way to distinguish MV types, although this might not be entirely quantitative (33). Flow cytometry might be another possible method to do so, as demonstrated by a recent study (30). Current hardware limitations make separation of nanometre-scale particles very cumbersome, necessitating further developments in the field before they can be routinely utilized in the MV biology. Such developments in the field of nanoparticle separation may facilitate our understanding of how different types of MVs are distributed over a size range. Another promising approach in this regard is the use of nanofluidic devices—while these have mainly been useful for characterization of larger eukaryotic extracellular vesicles, refinements to the underlying technology could expand the applicability of such methods to bacterial MV biology (34).

While it is tempting to draw quantitative conclusions from our fluorescence microscopy data, this would likely be a hasty endeavor, since the ”spots” visible upon overnight treatment with genotoxic agents could simply be membrane fragments that have not re-circularized. Furthermore, due to the limits of diffraction, smaller spots cannot be captured by the equipment used, leading to underrepresentation of smaller MVs—indeed, this was also observed for the SPT data when cryo EM data were analyzed. As such, we leave the data to be qualitative in nature with the recommendation that future studies make use of more advanced super-resolution microscopy techniques, such as stimulated emission depletion microscopy (STED). In any case, our use of fluorescence microscopy should not be discounted—the polar localization of LysSM in response to norfloxacin challenge is an important finding, and a similar observation was made for tailocins in *Pseudomonas* (35).

While there is a clear link between bacteriophage-encoded endolysins and OIMV formation, the detection of atypical OIMVs with multiple cytoplasmic compartments is more difficult to explain. These are reminiscent of MVs formed in gram-positive bacteria due to “bubbling cell death,” where the activity of a bacteriophage-encoded endolysin weakens the integrity of the peptidoglycan layer, thereby allowing the cell membrane to protrude into the extracellular matrix and pinch off as MVs (36). In this manner, it is conceivable that multiple cytoplasmic protrusions into the periplasm might occasionally be incorporated into a single larger vesicle. In such a scenario, the randomness associated with cell lysis is likely also a contributing factor; however, this remains to be tested. Another observation that is perhaps unusual is the fact that some of the OIMVs we detected seemed to have much smaller cytoplasmic compartments relative to the total MV size, and this has also been reported in previous studies (11). Such OIMVs appear to resemble small CMVs trapped within larger OMVs—since it has been presumed that OIMV formation requires the simultaneous disruption of both outer and inner membranes (meaning that the OM-IM gap would be maintained), our observations appear to partially contradict this fact. One explanation for the existence of such OIMVs is that these are, in fact, simply CMVs that are produced upon cell lysis and incorporate into larger OMVs when these fragments spontaneously re-circularize, although this hypothesis remains to be tested.

## MATERIALS AND METHODS

### Bacterial strains and growth conditions

Strains and plasmids used in this study are listed in Table 4. *E. coli* was maintained in regular LB (LB-Miller), while *S. maltophilia* was maintained in a low-salt LB formulation (LB-Lennox). *S. maltophilia* was also grown on *Pseudomonas* Isolation Agar (PIA) plates for selection of successful transformants. All liquid cultures were grown at 37°C in aerobic conditions, and agar plates were incubated at either 30°C or 37°C. The following antibiotics were used for selection in or against *E. coli*—trimethoprim at 50 μg/mL, kanamycin at 50 μg/mL, tetracycline at 10 μg/mL, and norfloxacin at 2 μg/mL. The following antibiotics were used for selection in *S. maltophilia*—trimethoprim at 50 μg/mL, chloramphenicol at 40 μg/mL, and tetracycline at 15–17 μg/mL. The following antibiotics were used for induction of stress responses in *S. maltophilia*—imipenem at 25 μg/mL, carbenicillin at 200 μg/mL, ciprofloxacin at 2 μg/mL, norfloxacin at 2 μg/mL, and mitomycin C at 1 μg/mL.

**TABLE 4:** Strains and plasmids used in this study

Strain or plasmid	Relevant characteristics^*a*^	Reference or source
*Escherichia coli* strains		
HB101	F^−^, *hsd*S20(r_B_^−^ m_B_^−^) *sup*E44 *leu*B6 *ara*14 *gal*K *lac*Y1 *pro*A2 *rps*L20 *xyl5 mtl*1 *rec*A13	(37)
SY327	F^−^, Δ(lac pro) argE(am) rif malA recA56λ pir	(38)
*Stenotrophomonas maltophilia* strains		
44/98	LMG 26,824, clinical isolate obtained from a patient in Italy (39)	BCCM/LMG^*b*^
44/98 *mal:egfp*	44/98, LysSM with C-terminal eGFP tag	This study
44/98 *ax21:mCherry atpG:egfp*	44/98, Ax21 with N-terminal mCherry tag and AtpG with C-terminal eGFP tag	This study
44/98 *ax21:mCherry mal:egfp*	44/98, Ax21 with N-terminal mCherry tag and LysSM with C-terminal eGFP tag	This study
44/98 Δ*mal*	44/98, *mal* deletion	This study
44/98 Δ*mal ax21:mCherry atpG:egfp*	44/98, *mal* deletion with Ax21 with N-terminal mCherry tag and AtpG with C-terminal eGFP tag	This study
Plasmids		
pRK2013	*ori*_colE1_, RK2 derivative, Kan^R^, *mob*^+^, *tra*^+^	(40)
pGPI-SceI-XCm	*ori*_R6K_, Tp^R^, Cm^R^, *traJ–I*, carries reporter protein XylE, carries I-SceI restriction site	(41)
pAx21-mCherry-N	pGPI-SceI-XCm with N-terminal ax21*:mCherry* fusion protein construct	This study
pAtpG-eGFP-C	pGPI-SceI-XCm with *egfp* sequence flanked by *atpG* upstream and downstream homology regions	This study
pMal-eGFP-C	pGPI-SceI-XCm with *egfp* sequence flanked by *mal* upstream and downstream homology regions	This study
pΔMal	pGPI-SceI-XCm with *mal* upstream and downstream homology regions	This study
pDAI-SceI-SacB	*ori*_pBBR1_, Tc^R^, carries counter-selectable marker SacB, carries I-SceI endonuclease-coding gene	(41)

^*a*^ Kan, kanamycin; Tp, trimethoprim; Cm, chloramphenicol; Tc, tetracycline.

^*b*^ BCCM/LMG, Belgian Coordinated Collection of Microorganisms/Laboratory of Microbiology, Ghent University.

### Genome analysis of *S*. *maltophilia* strain 44/98

DNA of *S. maltophilia* strain 44/98 was extracted from two biological replicates using an automated Maxwell DNA preparation instrument (Promega, Wisconsin). DNA extracts were treated with 2 mg/mL RNAse (5 μL per 100 μL of extract) and incubated at 37°C for 1 hour. DNA quality was checked using agarose gel electrophoresis, and DNA quantification was performed using the QuantiFluor ONE dsDNA System and the Quantus Fluorometer (Promega, Wisconsin). The genome was sequenced using the Illumina HiSeq 4000 System at the Oxford Genomics Centre (Oxford, United Kingdom). The quality of raw Illumina reads was assessed using FastQC version 0.11.9 (42), and the genome was subsequently assembled in Shovill version 1.1.0 (43), considering a minimal contig length of 500 bp and 150× depth. The quality of the final assembly was verified using the Quality Assessment Tool for Genome Assemblies (QUAST) version 5.2.0, which generates summary statistics including the number of contigs, N50, L50, and the GC content (44). To facilitate functional analysis, contigs were rearranged using progressiveMauve version 2.4.0 (45), considering a minimal locally colinear block (LCB) weight of 40 based on alignment with the *S. maltophilia* K279a reference genome (46). Finally, the assembly was functionally annotated using Prokka version 1.14.5 (47).

### Identification and analysis of phage sequences within the *S*. *maltophilia* genome

Prediction of prophage sequences was performed using Phage Search Tool with Enhanced Sequence Translation (PHASTEST) (48) using the Proksee genome analysis and visualization system (49). The genome of strain 44/98 was analyzed and bacterial sequence annotation was performed in “Deep” mode, making use of the PHASTEST Bacterial Sequence Database.

### Sample preparation for LC-MS/MS analysis

Three overnight cultures of *S. maltophilia* 44/98 WT were back-diluted in 100 mL of fresh LB-Lennox to reach an OD_600_ of 0.2, following which they were allowed to grow for 2 hours to reach the early exponential phase. A sub-MIC of ciprofloxacin was added to the treatment cultures, and all cultures were allowed to grow for a further 3 hours. Subsequently, cultures were centrifuged at 8,000 × *g* for 10 minutes at 4°C to pellet cells. Cell pellets were washed with ice-cold phosphate-buffered saline (PBS) and resuspended in a lysis buffer (6 M urea, 2 M thiourea, 50 mM ammonium bicarbonate), to which a cOmplete EDTA-free Protease Inhibitor Tablet (Roche) was added to prevent protein degradation. Cell suspensions were sonicated using the following parameters—1 second on, 8 seconds off at an intensity of 10% for 2 minutes. Cell debris was pelleted by centrifugation at 16,000 × *g* for 10 minutes at 4°C, and trichloroacetic acid was added to the supernatants to reach a final concentration of approximately 2% (vol/vol) to precipitate proteins overnight. The following day, precipitated proteins were pelleted by ultracentrifugation and washed with ice-cold PBS. Proteins were dried in a SpeedVac vacuum concentrator and resuspended in 50 mM ammonium bicarbonate. Proteins were quantified in triplicate using a Coomassie Protein Assay Kit (Thermo Scientific) with bovine serum albumin (BSA) as a standard. Samples were stored at –20°C until required. All growth and extraction experiments were performed simultaneously on the same day.

### LC-MS/MS analysis of the ciprofloxacin response

Previously, a semi-quantitative experiment indicated the induction of the maltocin gene cluster upon treatment of *S. maltophilia* strain 44/98 (14). To confirm this finding, we repeated this experiment in a quantitative proteomics analysis using three parallelly grown biological replicates of cellular extracts from cells treated with ciprofloxacin compared to an untreated control.

A portion (10 μg) of each sample was dried in a SpeedVac vacuum concentrator and resuspended in 50 mM ammonium bicarbonate to reach a total volume of 50 μL. Samples were heated at 80°C for 10 minutes, following which they were reduced using DTT (50 mM, 10 minutes at 60°C) and alkylated using IAA (150 mM, 20 minutes in the dark at room temperature). Sequencing-grade modified trypsin (Promega) was added in a 1:50 ratio, and samples were incubated overnight in a water bath maintained at 37°C to allow for complete trypsinization. The following day, samples were acidified using formic acid to stop the action of trypsin, and samples were centrifuged to precipitate any insoluble substances. The digestion mixtures were passed through 0.22 μm filters and dried in a SpeedVac vacuum concentrator. Dried peptides were resuspended in 0.1% trifluoroacetic acid in water/acetonitrile (98:2 vol/vol) (loading solvent), and half of each sample was injected on an UltiMate 3000 RSLCnano UPHLC system (Thermo Scientific) connected in-line to an LTQ Orbitrap Elite mass spectrometer (Thermo Scientific) equipped with a PST-pneu-Nimbus dual-column source (Phoenix S&T, Pennsylvania). Trapping was performed at 10 μL/min for 4 minutes in solvent A on a 20 mm trapping column made in-house, with a 100 μm internal diameter and 5 μm C18 Reprosil-HD beads (Dr. Maisch, Germany). The samples were loaded on a 200 cm micro pillar array column (µPAC) with C18-endcapped functionality (PharmaFluidics, Belgium), mounted in the UltiMate 3000 column oven at 50°C. To ensure proper ionization, a fused silica PicoTip emitter with a 10 µm inner diameter (New Objective, Massachusetts) was connected to the µPAC outlet union with a grounded connection. Peptides were eluted using a non-linear gradient reaching 9% of 0.1% formic acid in water/acetonitrile (2:8 vol/vol) (solvent B) in 15 minutes, 33% solvent B in 105 minutes, 55% solvent B in 125 minutes, and 99% solvent B in 135 minutes, with an initial flow rate of 750 nL/min that was subsequently decreased to 300 nL/min, followed by a 15-minute wash reaching 99% solvent B and re-equilibration with 0.1% formic acid in water (solvent A). The mass spectrometer was operated in data-dependent positive ionization mode, set to automatically switch between MS and MS/MS acquisition for the 20 most abundant peaks for a given MS spectrum. Source voltage was set at 1.8 kV, and capillary temperature was set at 275°C. Full-scan MS spectra (300–2,000 *m*/*z*; AGC target of 3 × 106 ions, maximum ion injection time of 100 ms) were acquired in the Orbitrap with a precursor resolution of 60,000 at 400 *m*/*z*. The 20 most intense ions that satisfied a defined set of selection criteria (AGC target 5 × 103 ions, maximum ion injection time of 20 ms, centroid spectrum data type, exclusion of unassigned and singly positive charged precursors, dynamic exclusion time of 20 seconds) were subsequently isolated in the linear ion trap and fragmented in the high-pressure cell through collision-induced dissociation (CID). Collision energy was set to 35 V, and the polydimethylcyclosiloxane background ion at 445.120028 Da was used for internal calibration.

Data were processed using MaxQuant (version 2.4.4.0) and the integrated search engine Andromeda (50). Identification of proteins was done by searching the raw data files against a database comprised of protein sequences identified from the genomic analyses performed earlier. Carbamidomethylation of cysteine and oxidation of methionine were selected as fixed or variable modifications, respectively. Trypsin was selected as the proteolytic enzyme, and up to two missed cleavages were allowed. The intensities of the proteins were determined by summing up the intensities of razor and unique peptides, and these were normalized to obtain label-free quantification (LFQ) intensities. Missing values were replaced with low-abundance measurements generated from the normal distribution. Statistical significance was assessed with Perseus (version 2.1.3.0) using a two-sample t-test (fold-change >2, *q*-value <0.05). Identified proteins were searched against the UniProt database to identify their orthologs in the genome of the type-strain K279a.

### Construction of *ax21:mCherry*, *atpG:egfp*, *mal:egfp*, and Δ*mal* mutants

The 44/98 deletion and insertion mutants used in this study were constructed using the protocol described previously (24). The *ax21:mCherry* mutants were constructed by replacing the endogenous *ax21* gene with the corresponding mCherry-tagged version, while the *atpG:egfp* and *mal:egfp* mutants were constructed as in-frame fusions with *egfp*. Construction of all plasmids was outsourced to GenScript Biotech (Piscataway, New Jersey). Verification of all mutations was performed by sequencing of PCR fragments generated using various primer pairs (Table S2).

To construct the *ax21:mCherry* replacement allele, regions upstream (393 bp) and downstream (500 bp) of *ax21* were selected. The upstream region was comprised of mostly intergenic sequences and a small portion of the preceding gene (*smlt0386*), while the downstream region was made up entirely of intergenic sequences. The replacement gene itself was comprised of the mCherry-coding sequence placed *after* the signal peptide sequence, followed by a short flexible linker (coding for the amino acid sequence GSGSGS) and the gene itself (Material S9). The entire allele (2.5 kb) was synthesized and cloned into pGPI-SceI-XCm to give pAx21-mCherry-N.

To construct the insert allele for *atpG*, regions upstream (839 bp) and downstream (861 bp) of the gene were selected. The upstream region consisted of a portion of the gene immediately upstream (*atpD*), while the downstream region was comprised of the gene itself *without* its stop codon. Since *atpG* is found on the minus strand, the reverse complements of the eGFP-coding sequence and linker were used. The final insert was made up of the upstream homology sequence (*atpD*), the eGFP and linker (coding for the amino acids GSGSGS) sequences, and the downstream homology sequence (*atpG*) (Material S9). This sequence was synthesized and cloned into pGPI-SceI-XCm to give pAtpG-eGFP-C.

To construct the insert allele for *mal*, regions upstream (200 bp) and downstream (300 bp) of the gene were selected. The upstream region consisted of a portion of the gene immediately upstream (*smlt1053*), while the downstream region was comprised of a portion of the gene itself *without* its stop codon. *mal* is also found on the minus strand; hence, the reverse complements of the eGFP-coding sequence and linker (coding for the amino acids GSGSGS) were used once again. The final insert was made up of the upstream homology sequence (*smlt1053*), the eGFP and linker sequences, and the downstream homology sequence (*smlt1055*) (Material S9). This sequence was synthesized and cloned into pGPI-SceI-XCm to give pMal-eGFP-C.

To construct the deletion allele for *mal*, regions upstream (200 bp) and downstream (300 bp) of the gene were selected. The upstream portion consisted of a portion of the gene immediately upstream (*smlt1053*), while the downstream region was comprised of a portion of the gene immediately downstream (*smlt1055*) (Material S9). This sequence was synthesized and cloned into pGPI-SceI-XCm to give pΔMal.

While the genome of strain 44/98 was used for the PHASTEST prophage predictions, the genome of the type-strain K279a was used for all bioinformatic predictions pertaining to mutagenesis in this study, since it is much more thoroughly annotated. Previous proteomics experiments have shown that proteins isolated from strain 44/98 are easily identifiable based on genetic information from the type-strain, indicating that the two strains are highly similar (51). In the context of strain K279a sequences, the original gene and locus tags were retained.

### Growth curve construction

Three overnight cultures of *S. maltophilia* 44/98 WT and Δ*mal* were back-diluted to an OD_600_ of 0.01 in 50 mL of LB-Lennox. Cultures were incubated in a rotary shaker at 37°C and 200 rpm, and OD_600_ measurements were taken at intervals of 30 minutes for 12 hours. In addition to monitoring the growth of the WT and Δ*mal* mutant under normal conditions, growth curves were also constructed for cultures exposed to sub-MIC concentrations of norfloxacin and mitomycin C in parallel. For these experiments, the corresponding antibiotic was added once cultures had reached an OD_600_ between 0.3 and 0.4. Graphs were plotted using Matplotlib (52) using observations from three biological replicates for each condition grown on the same day.

### Biofilm assay

Three overnight cultures of *S. maltophilia* 44/98 WT and Δ*mal* were normalized to an OD_600_ of 2.0 and subsequently back-diluted in a ratio of 1:100 in a 3-(*N*-morpholino)propanesulphonic acid (MOPS) minimal medium supplemented with 5 mM glucose and 250 μM L-methionine (53). An amount of 100 μL of these suspensions was inoculated in each well of separate 96-well flat-bottom polystyrene plates. Plates were sealed using microporous sheets (Qiagen) and incubated statically at 37°C for 4 hours to allow cells to adhere. Following this, the medium was discarded, and cells were washed with 150 μL of sterile PBS to remove non-adherent cells. Fresh MOPS medium of 100 μL was added to each well, and plates were sealed once again. Three conditions of stress were tested per plate, viz., MOPS without any antibiotic, and MOPS supplemented with sub-MICs of either norfloxacin or mitomycin C. Three biological conditions with eight technical replicates each grown on the same day were tested per condition. Plates were incubated statically at 37°C for 24 or 48 hours to allow biofilm formation.

After overnight incubation, the medium was discarded, and the cells were washed with 150 μL of sterile PBS to remove non-adherent cells. Cells were fixed with 99% (vol/vol) methanol for 15 minutes, following which methanol was aspirated and plates were air-dried. 100 μL of 0.01% (wt/vol) crystal violet was added to each well, and plates were incubated for 30 minutes at ambient temperature. Following staining, plates were washed thoroughly under running water to remove unbound crystal violet. Bound crystal violet was liberated by the addition of 150 μL of 33% (vol/vol) glacial acetic acid, and absorbance was measured at 590 nm. Graphs were plotted using Matplotlib (52), and unpaired t-tests were performed using an online t-test calculator (GraphPad) to assess the significance of results.

### Isolation of MVs

Three overnight cultures of *S. maltophilia* 44/98 WT and Δ*mal* cultures were back-diluted in 50 mL of fresh LB-Lennox at a ratio of 1:50 and allowed to grow until they reached the mid-exponential phase (OD_600_ between 0.65 and 0.75). Vesiculation was stimulated by the addition of a sub-MIC concentration of β-lactam (imipenem or carbenicillin), fluoroquinolone (ciprofloxacin or norfloxacin), or mitomycin C, and cultures were allowed to grow for 3 hours. Subsequently, cultures were centrifuged at 8,000 × *g* for 10 minutes at 4°C to pellet cells, and the supernatant was passed through a 0.45-μm-pore-size polyethersulphone (PES) membrane filter (Merck Millipore, MA, USA). Filtered supernatant (40 mL) was transferred to a polycarbonate vial and ultracentrifuged at 108,800 × *g* for 3 hours at 4°C (Avanti J-30I, Beckman Coulter, California) to pellet MVs. The MV pellets were resuspended in PBS and stored at –20°C until required. All samples were collected on the same day.

### MV quantification and size determination

The concentration and diameters of MVs were determined by single-particle tracking (SPT) using a NanoSight LM10 HS system (Malvern Panalytical, United Kingdom) equipped with a 405 nm laser. Following the manufacturer’s instructions, a preliminary analysis was performed, allowing for the dilution factor to the optimal concentration range of the instrument. A dilution series of the original solution was made in PBS and measured. For the actual measurement, a dilution factor of 10 was used for the mutant, whereas a dilution factor of 100 was used for the WT samples. Three biological replicates of each condition were accordingly diluted in PBS. A PBS solution was used as a negative control. Movies of 30 seconds were recorded and analyzed using the NTA Analytical Software version 2.3 (Malvern Panalytical, United Kingdom). Calculations were made as previously described (54). Graphs were plotted using Matplotlib (52), and unpaired t-tests were performed using an online t-test calculator (GraphPad) to assess the significance of results.

### Flow cytometry

Three overnight cultures of *S. maltophilia* 44/98 WT and Δ*mal* cultures were back-diluted in 50 mL of fresh LB-Lennox at a ratio of 1:50 and allowed to grow until they reached mid-exponential phase (OD_600_ between 0.65 and 0.75). Vesiculation was stimulated by the addition of a sub-MIC concentration of norfloxacin, and cultures were allowed to grow for 3 hours. To remove growth medium components, cells were washed twice by centrifugation at 8,000 × *g* for 10 minutes at 4°C, followed by careful removal of the supernatants and resuspension in an identical volume of 0.22 µm filtered peptone-buffered saline (8.5 g/l NaCl, 1 g/l bacteriological peptone) (Oxoid).

Flow cytometry measurements were performed on an Attune NxT Acoustic Focusing Cytometer (Thermo Scientific) equipped with blue (488 nm) and red (637 nm) lasers, using Focusing Fluid (Thermo Scientific) as the sheath fluid. Instrument performance was checked using Attune Performance Tracking Beads (Thermo Fisher Scientific, USA) according to the manufacturer’s instructions. Data were recorded at a flow rate of 25 µL/min for a maximum volume of 100 µL or until 20,000 events were recorded. Experiments for all biological replicates were performed on the same day.

For total cell count (TCC) analysis, staining was performed three times to reduce technical variation using 1% (vol/vol) SYBR Green I (SG; 10,000× concentrate in dimethyl sulfoxide, DMSO) (Invitrogen, USA), followed by an incubation for 13 minutes at 37°C in the dark (55, 56). For the live/dead count (LDC) analysis, staining was performed in triplicate, combining 1% (vol/vol) SG with propidium iodide (PI; 50× dilution of 20 mM PI in DMSO). Using this dye combination (“SGPI”), samples were incubated in the dark at 37°C for 13 minutes. Samples were analyzed immediately after incubation (55).

Data were acquired using the Attune software (version 6.2.3269.1), using precedence density visualizations and histogram plots. Scatter and fluorescence parameters were displayed on a logarithmic scale. The voltages of detection channels were optimized using the “voltage walk” approach (57), with thresholds set on the blue laser’s primary fluorescence channel (BL1; 530/30 nm) and the side scatter channel (SSC) to eliminate noise while retaining particles of interest. Control samples included 0.22 µm filtered Focusing Fluid, stained peptone-buffered saline, and the unstained samples for TCC analysis, with an additional SGPI-stained heat-killed control (20 min at 80°C) for LDC analysis.

Raw flow cytometry data were exported as FCS file format and imported into R (version 4.3.2) using the flowCore package (version 2.12.2) (58) and further analyzed using the PhenoFlow (version 1.1.2) (https://github.com/rprops/PhenoFlow), flowViz (version 1.66.0) (59), Biobase (version 2.62.0), and grid (version 4.3.2) and gridExtra (version 2.3) packages. All fluorescent and scatter signals were displayed and analyzed using the voltage peak height (-H) parameter. Parameters of interest were transformed using the hyperbolic arcsine function. Coincidence events were removed from the data set by gating on the SSC-A vs. SSC-H plot, before gating the populations of interest—SG-positive for TCC, membrane-intact or membrane-damaged for LDC (Material S6). For the comparison of the proportion of dead cells in WT and Δ*mal* samples, a graph was plotted using Matplotlib (52) and an unpaired t-test was performed using an online t-test calculator (GraphPad) to assess the significance of the results.

### Fluorescence microscopy

Three overnight cultures of *S. maltophilia* mutants with fluorophore insertions were back-diluted in fresh LB-Lennox at a ratio of 1:50 and allowed to grow for 3 hours. When the cultures reached an OD_600_ between 0.3 and 0.4, the appropriate antibiotic was added, and cultures were grown for an additional 2 hours. Meanwhile, plastic microscopy dishes (refractive index 1.5) were coated with 0.1% (wt/vol) poly-L-lysine for 20 minutes, following which they were washed with sterile MilliQ water and air-dried in a laminar flow hood. For imaging, 200 μL of each culture was pipetted onto each poly-L-lysine-coated dish and placed in a laminar flow hood for 20 minutes. After incubation, the excess culture was removed, and plates were allowed to air-dry for an additional 10 minutes. A drop of immersion oil (refractive index 1.52) was placed on the dish before transferring it to the microscope stage. For time-lapse microscopy, samples were prepared in the same way described above—prior to imaging, 20 μL of high-concentration norfloxacin was added directly to air-dried plates for a duration of 1 minute and subsequently removed. Experiments were performed across multiple days under similar conditions.

Samples were visualized using a spinning disk confocal microscope (Nikon Eclipse Ti, Japan) equipped with an MLC 400 B laser box (Agilent Technologies, California, USA), a Yokogawa CSU-X confocal spinning disk device (Andor, Belfast, United Kingdom), an iXon Ultra EMCCD camera (Andor Technology, Belfast, United Kingdom), and a Plan Apo VC 100 × 1.4 NA oil immersion objective lens (MRD01902, Nikon, Japan). A 1.5× zoom lens was used for additional magnification on the camera. The NIS Elements software package (Nikon, Japan) was used for imaging. The 488 nm and 561 nm diode laser lines were applied sequentially to excite the eGFP and mCherry-tagged proteins, respectively, and fluorescence emission was detected through a quad band filter (440/40, 521/21, 607/34, and 700/45). Exposure time was set to 300 ms for mCherry and 1 s for eGFP, and 8× averaging was used to improve the signal-to-noise ratio. 16-bit images were recorded with an EM gain of 300 (without binning). The image size was 512 × 512 pixels, with a pixel size of 90 nm. For time-lapse videos, images were taken at 5-second intervals for a duration of 5 minutes each, with the Perfect Focus System (PFS) enabled. Samples were maintained at ambient temperature during imaging. Images were analyzed using Fiji (60).

### Cryogenic electron microscopy

A portion (4 µL) of sample was applied to glow-discharged Lacey Formvar/Carbon 400 mesh copper grids (Ted Pella Inc., California). Grids were blotted and plunge-frozen in liquid ethane using a Leica EM GP2 Plunge Freezer (Leica Microsystems, Germany) operated at 95% humidity, using sensor blotting with 2 mm additional move and blotting times of 4.5 seconds (WT and Δ*mal* cultures exposed to norfloxacin), 5 seconds (Δ*mal* culture exposed to mitomycin C), or 5.5 seconds (WT culture exposed to mitomycin C). Micrographs were recorded on a JEM-1400 Plus transmission electron microscope (JEOL, Tokyo) operated at 120 keV, equipped with a Ruby charge-coupled device camera (JEOL, Tokyo) at a magnification of 60,000×, corresponding to a pixel size of 2.639 Å at the specimen level. At least 100 images were taken for each sample using random fields of view. Three biological replicates were collected per condition, and experiments were performed across multiple days. Graphs were plotted using Matplotlib (52), and statistical significance between population proportions was assessed using a chi-square test of independence, followed by post hoc pairwise comparisons using Z-tests and a Bonferroni correction.

## Data Availability

The genome of *S. maltophilia* strain 44/98 is available at the ENA depository under the accession number GCA_977063385. The mass spectrometry proteomics data are available at the PRIDE database with the data set identifier PXD069601.

## SUPPLEMENTAL MATERIAL

Supplementary figures and tables jb.00158-26-s0001.docx Tables S1 and S2, and Figures S1 to S5.

Supplementary Material S6 jb.00158-26-s0002.docx Figures S6.1 to S6.3.

Supplementary Material S7 (Part 1 of 2) jb.00158-26-s0003.docx Figures S7.1 to S7.6.

Supplementary Material S7 (Part 2 of 2) jb.00158-26-s0004.docx Figures S7.7 to S7.12.

Supplementary figures and material jb.00158-26-s0005.docx Figure S8, Supplementary Material S9, and legends for Supplementary Movies S1 and S2.

Table S1 jb.00158-26-s0006.xlsx List of *S. maltophilia* 44/98 proteins that display a significant change in abundance upon treatment with ciprofloxacin.

Supplementary Tables jb.00158-26-s0007.xlsx Tables S6.1 and S6.2.

Movie S1 jb.00158-26-s0008.mp4 Upregulation of *mal* in response to norfloxacin exposure. Time-lapse video (3 min) showing the upregulation of *mal* in response to excessive norfloxacin (10 µg/mL). The arrow shows a cell with polar foci appearing, corresponding to the localization of LysSM. Scale bar, 5 µm.

Movie S2 jb.00158-26-s0009.mp4 Upregulation of *mal* in response to norfloxacin exposure. Time-lapse video (3 min) showing the upregulation of *mal* in response to excessive norfloxacin (10 µg/mL). Multiple cells in this field of view show polar foci appearing, corresponding to the localization of LysSM, and the white arrow shows one example. Scale bar, 5 µm.

ASM does not own the copyrights to Supplemental Material that may be linked to, or accessed through, an article. The authors have granted ASM a non-exclusive, world-wide license to publish the Supplemental Material files. Please contact the corresponding author directly for reuse.
