# Supplementary material for: DNA damage-associated vesicle production in Stenotrophomonas maltophilia is mediated by the maltocin endolysin
Source: J Bacteriol. 2026 Jun 25;208(7):e00158-26. doi: 10.1128/jb.00158-26 (PMC13393414; doi:10.1128/jb.00158-26)
Supplement: Supplementary figures and tables — Tables S1 and S2, and Figures S1 to S5. [file jb.00158-26-s0001.docx]

**DNA damage-associated vesicle production in *Stenotrophomonas maltophilia* is mediated by a cryptic tailocin endolysin**

**Supplementary Table 1. List of *S*. *maltophilia* 44/98 proteins that display a significant change in abundance upon treatment with ciprofloxacin.** Provided as a separate Excel spreadsheet.

**Supplementary Table 2. List of screening primers used.**

| **Type of primer** | **Primer sequence (5’ 🡪 3’)** |
| --- | --- |
| *egfp* gene-specific | FP: CACATGAAGCAGCACGACTT  RP: TGCTCAGGTAGTGGTTGTCG |
| *atpG* gene-specific | FP: TGTGCTTTGAACGAACGCGG  RP:TGATTTCGCGTCCGCTTGCCAT |
| *mal* gene-specific | FP: CATTGATGGTGATGCCGCG  RP: AGACGCTCTGTTGAACCTGG |
| *mal* flanking region | FP: CATTGATGGTGATGCCGCG  RP: AGACGCTCTGTTGAACCTGG |
| *ax21* gene-specific | FP: ATGAAGAATTCGCTGATTGCTCTGG  RP: GCGGAATCCAATTCGTTGCAAT |


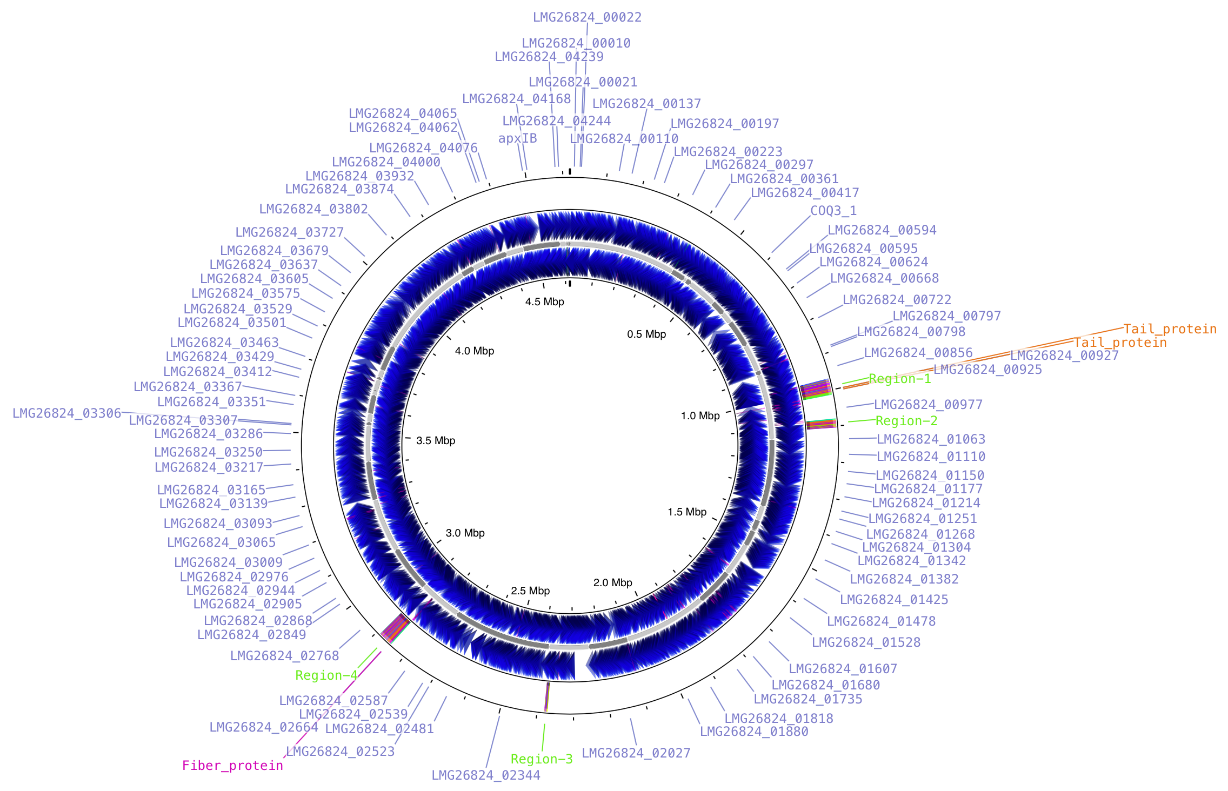
**Supplementary Material 1. Summary of PHASTEST predicted prophage regions**

**Figure S1. PHASTEST predicted prophage regions.** Based on PHASTEST predictions, there exist four prophage gene clusters within the genome of *S*. *maltophilia* strain 44/98 – of these, two clusters (regions 1 and 4) appear to be bacteriophage clusters, one cluster (region 2) appears to be a tailocin cluster, and the last (region 3) appears to be the remnant of a bacteriophage.

**Table S1.1. Summary of prophage regions based on PHASTEST predictions.**

| **Region** | **Length (kb)** | **Position** | **GC content (%)** | **Number of proteins** | **Score** | **Completeness** |
| --- | --- | --- | --- | --- | --- | --- |
| 1 | 43.3 | 968474–1011850 | 64.01 | 48 | 130 | Intact |
| 2 | 22.6 | 1080534–1103224 | 66.22 | 32 | 140 | Intact |
| 3 | 5.8 | 2372908–2379589 | 61.97 | 9 | 116 | Intact |
| 4 | 39.7 | 2850018–2889800 | 64.50 | 56 | 150 | Intact |

**Supplementary Material 2. Figures of remaining predicted prophage regions in *S*. *maltophilia* strain 44/98**

**
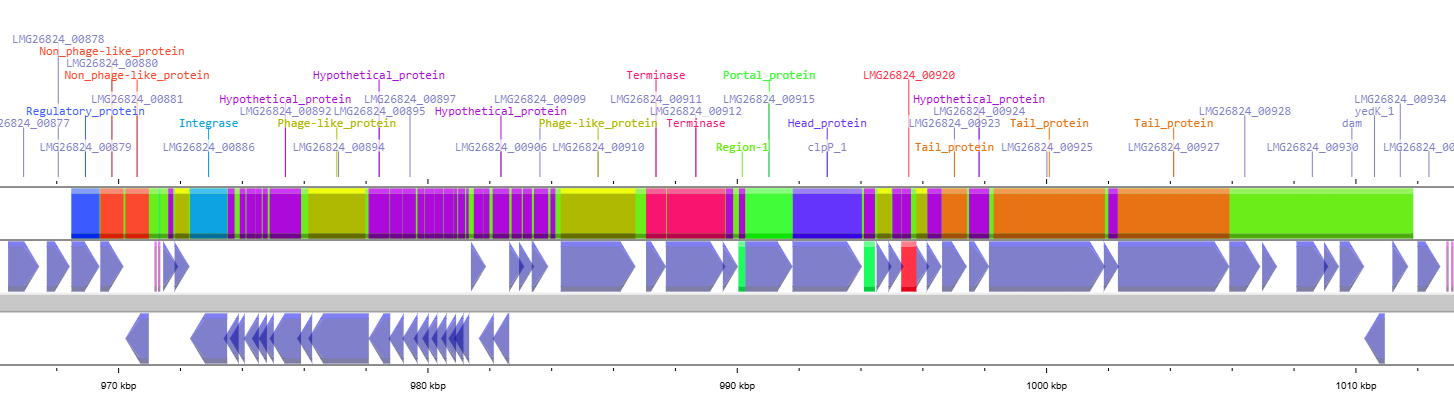
**


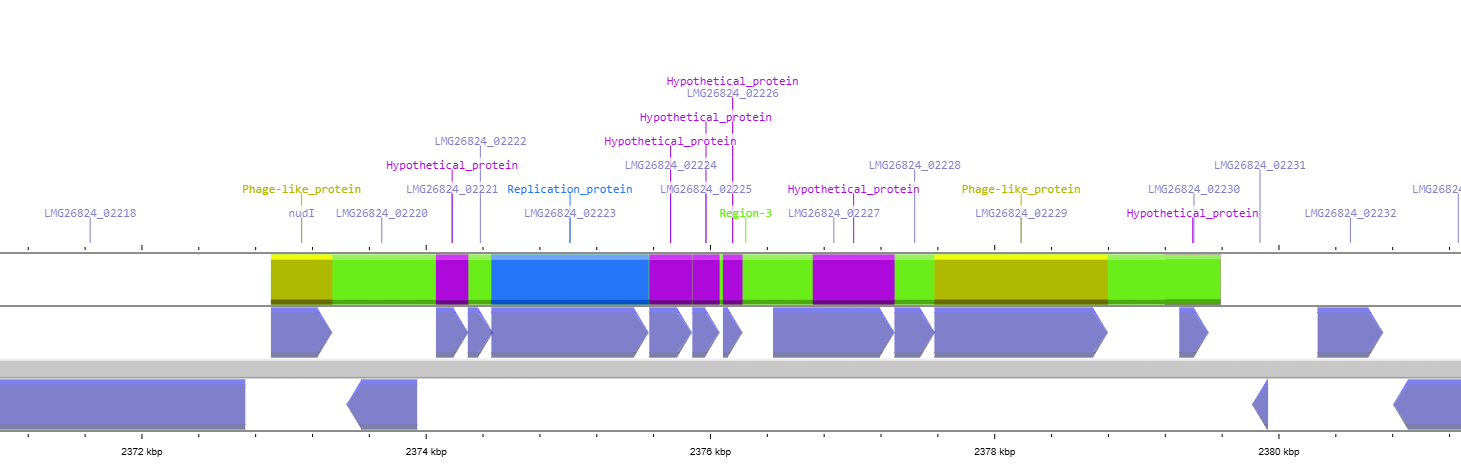


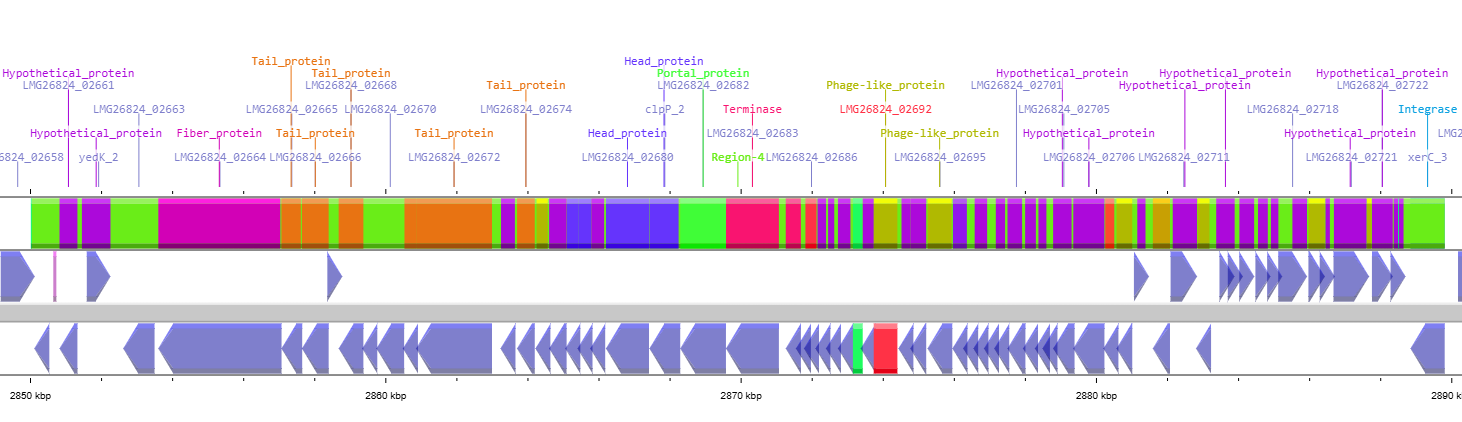


**Figure S2. PHASTEST predicted prophage regions.** Based on predictions, all the prophage regions were annotated as ‘intact,’ although region 4 is considerably shorter than the others and lacks several key phage-associated genes. The lytic enzymes and putative holin/spanin candidate genes are coloured in red and green respectively in the lower panels.

Top – region 1, middle – region 3, bottom – region 4.


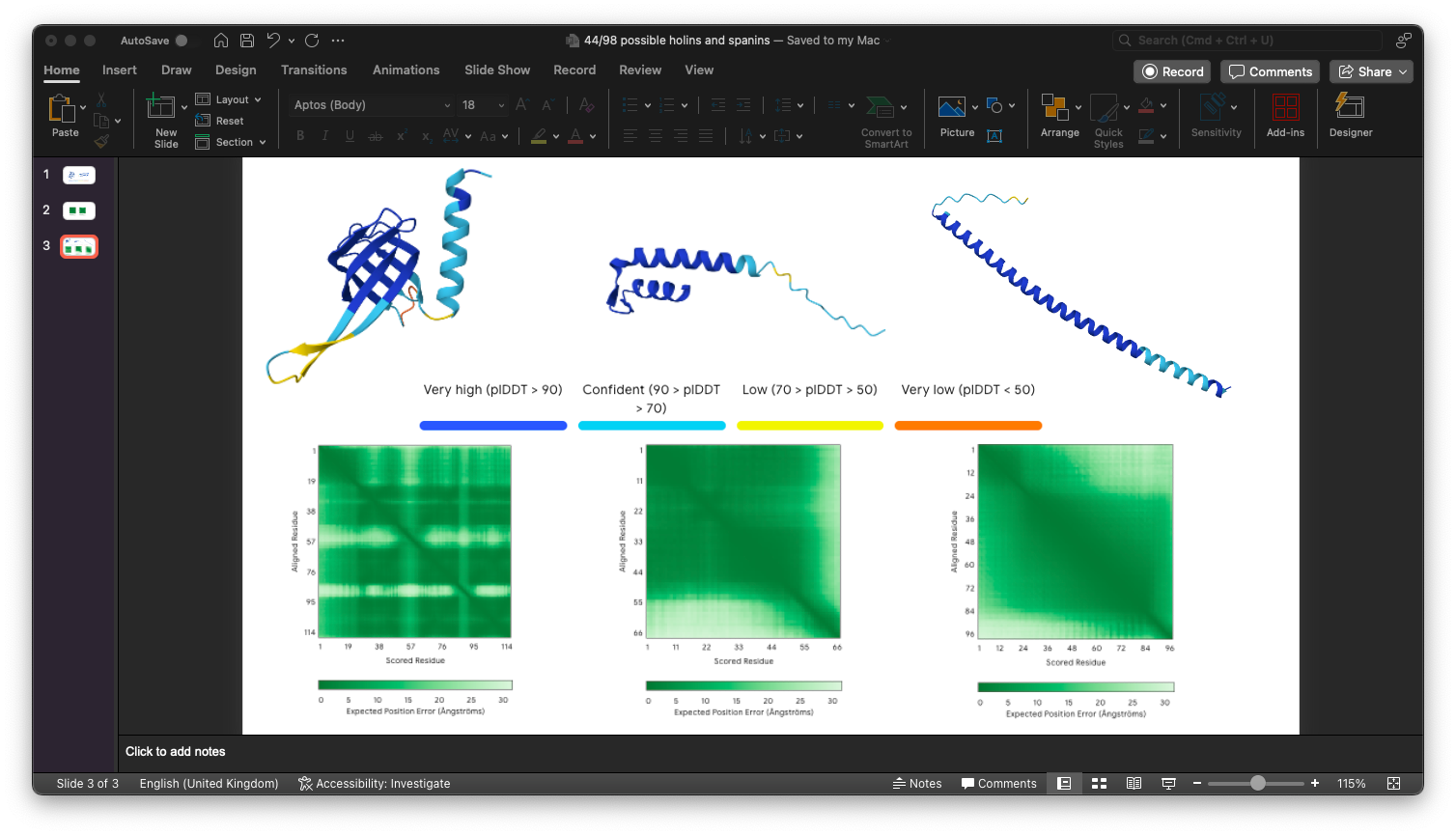
**Supplementary Material 3. Putative holins and spanins within *S*. *maltophilia* strain 44/98 predicted prophage regions**


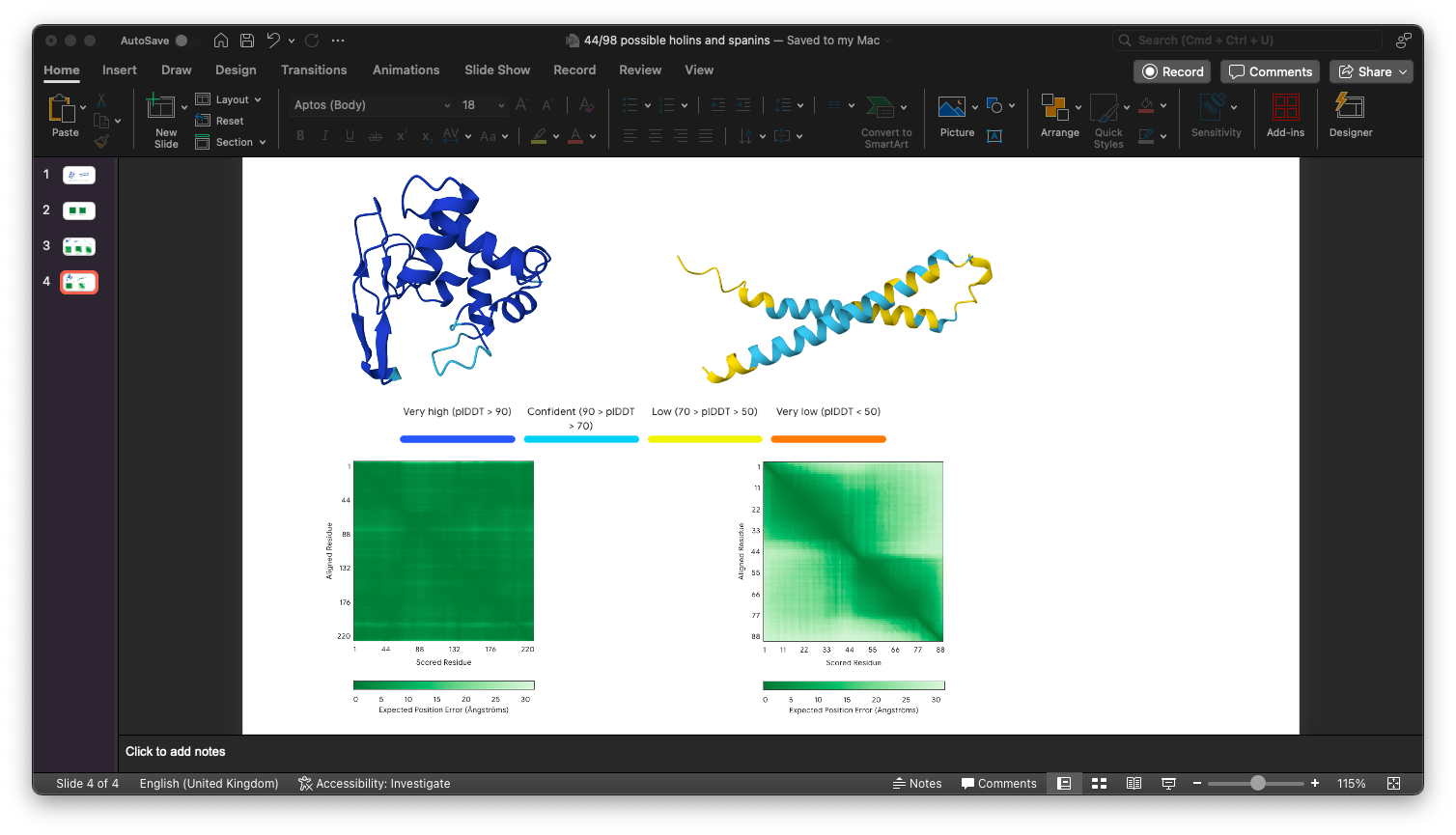
**Figure S3.1. Lysin and putative holin and spanin within predicted prophage region 1.** The holin and spanin-coding genes were annotated as holins due to their close proximity to the endolysin-coding gene as well as their characteristic transmembrane domains. Protein structures predicted by AlphaFold 3.0. Predicted local distance different test (pLDDT) score heatmap and predicted aligned error (PAE) scores (lysin, left; holin, middle; spanin, right) taken from AlphaFold 3.0.

**Figure S3.2. Lysin and holin within predicted prophage region 4.** The possible holin-coding gene was annotated as such due to its close proximity to the endolysin-coding gene as well as its characteristic transmembrane domains. Protein structures predicted by AlphaFold 3.0. Predicted local distance different test (pLDDT) score heatmap and predicted aligned error (PAE) scores taken from AlphaFold 3.0.


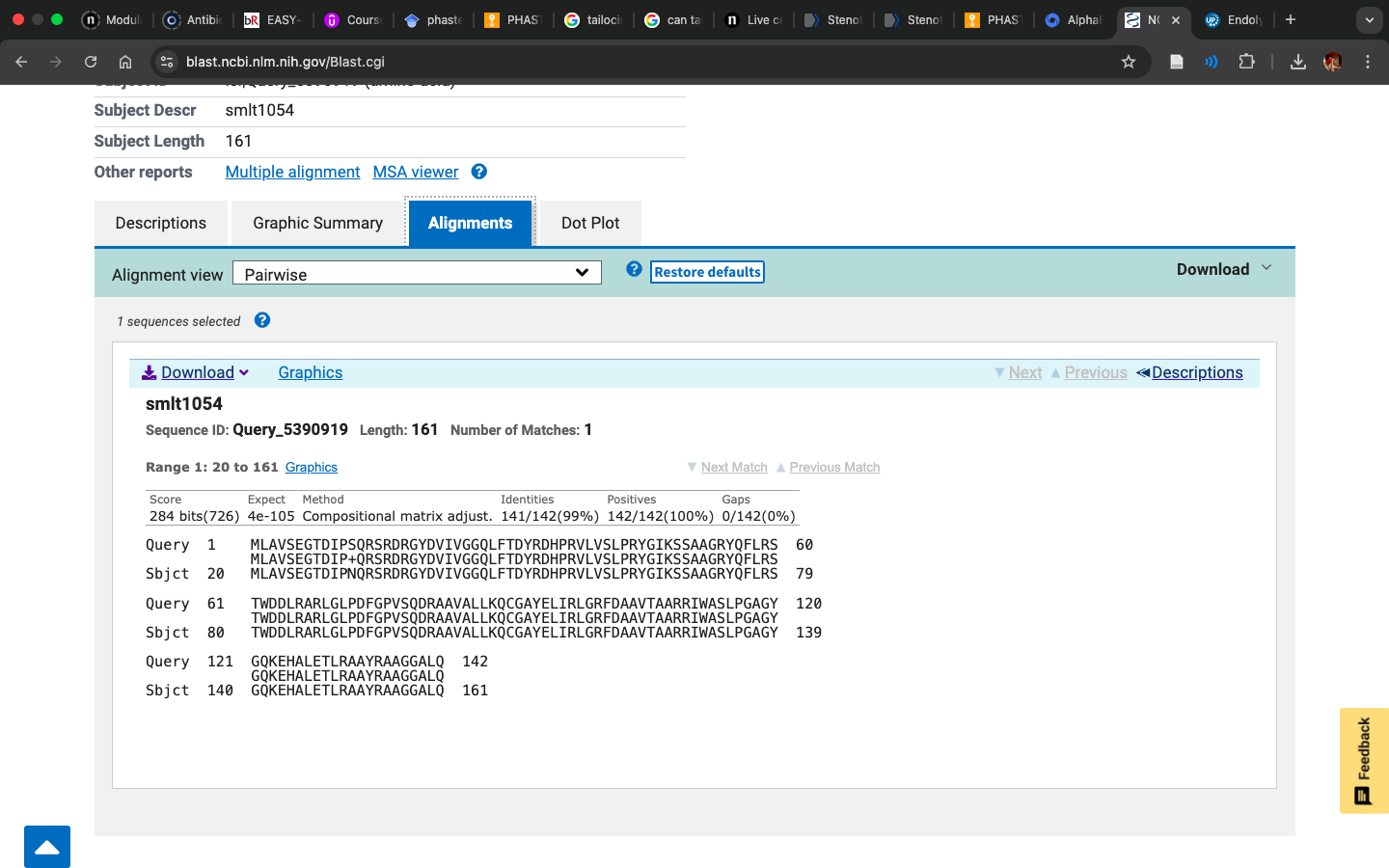
**Supplementary Material 4. Protein sequence alignments between endolysins**


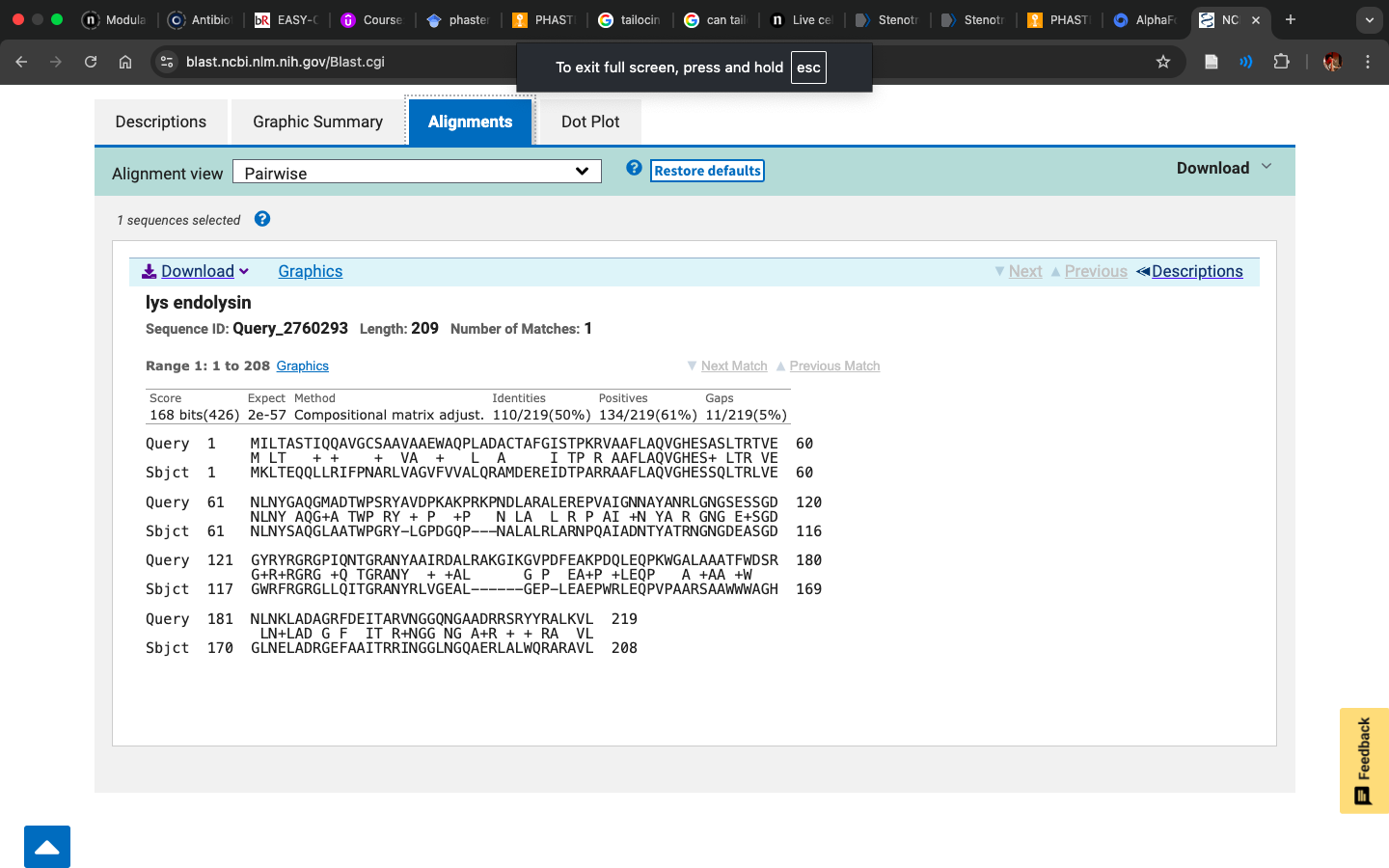
**Figure S4.1. Sequence alignment between maltocin endolysin from *S*. *maltophilia* strains 44/98 and K279a.** The maltocin endolysins from strains 44/98 and K279a are virtually identically, with the exception of 19 aa at the N-terminus of the K279a endolysin – this corresponds to a small α-helix and a small portion of another. However, based on available proteomic data, this was found to be an annotation error, since these extra amino acids are present even in the endolysin from strain 44/98.

**Figure S4.2. Sequence alignment between *S*. *maltophilia* lysozyme and the *P*. *aeruginosa* endolysin.** The *S*. *maltophilia* lysozyme found in prophage region 2 (query) and the endolysin previously described in *P*. *aeruginosa* (subject) share many identities and even more positives, indicating high similarity between the two proteins.


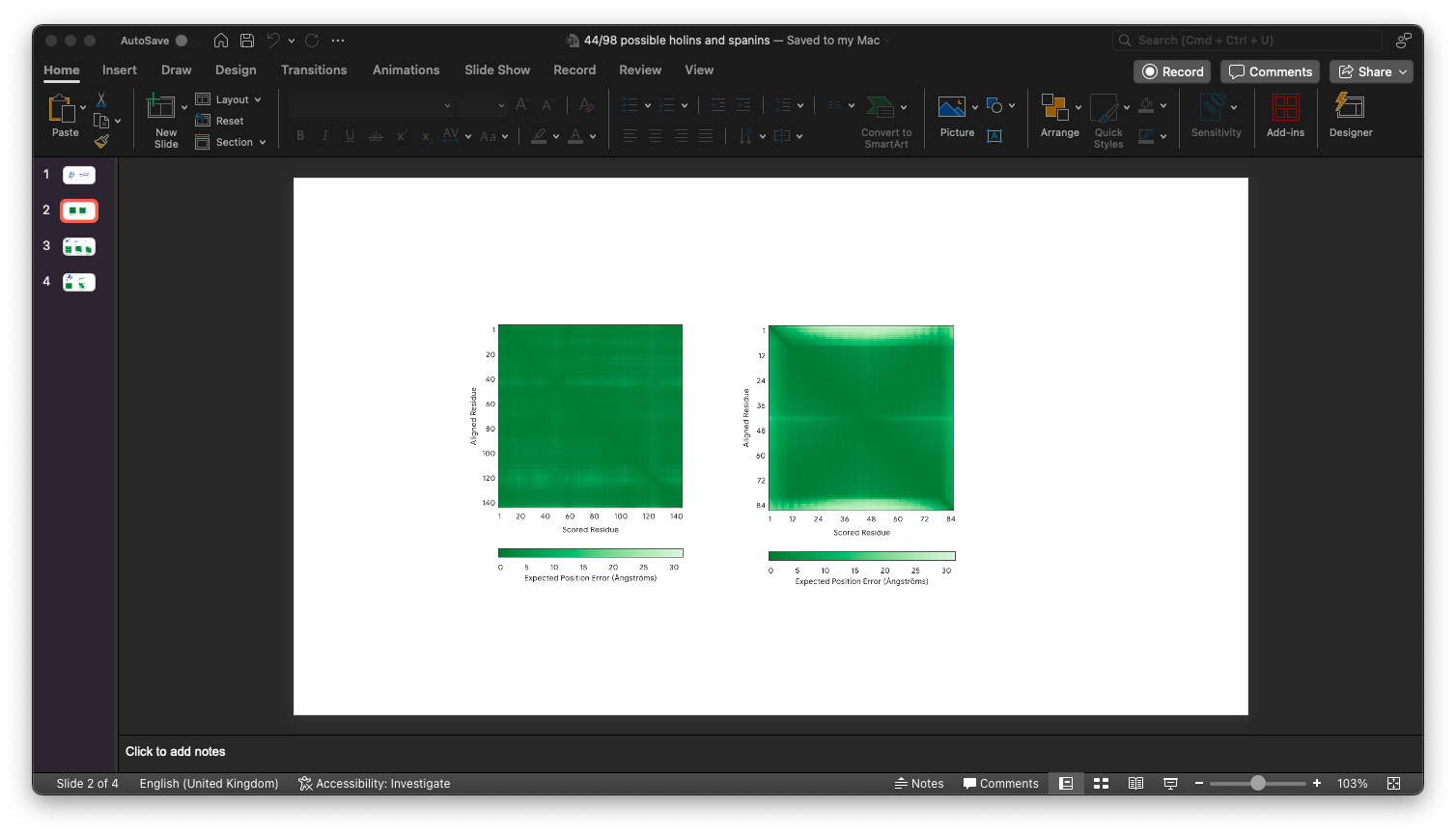
**Supplementary Material 5. AlphaFold metrics for *S*. *maltophilia* strain 44/98 maltocin endolysin and potential holin**

**Figure S5. AlphaFold metrics for *S*. *maltophilia* strain 44/98 maltocin endolysin and potential holin.** Predicted aligned error (PAE) scores taken from AlphaFold 3.0.
