# Supplementary material for: DNA damage-associated vesicle production in Stenotrophomonas maltophilia is mediated by the maltocin endolysin
Source: J Bacteriol. 2026 Jun 25;208(7):e00158-26. doi: 10.1128/jb.00158-26 (PMC13393414; doi:10.1128/jb.00158-26)
Supplement: Supplementary Material S6 — Figures S6.1 to S6.3. [file jb.00158-26-s0002.docx]

**Supplementary Material 6. Data from flow cytometry experiments**


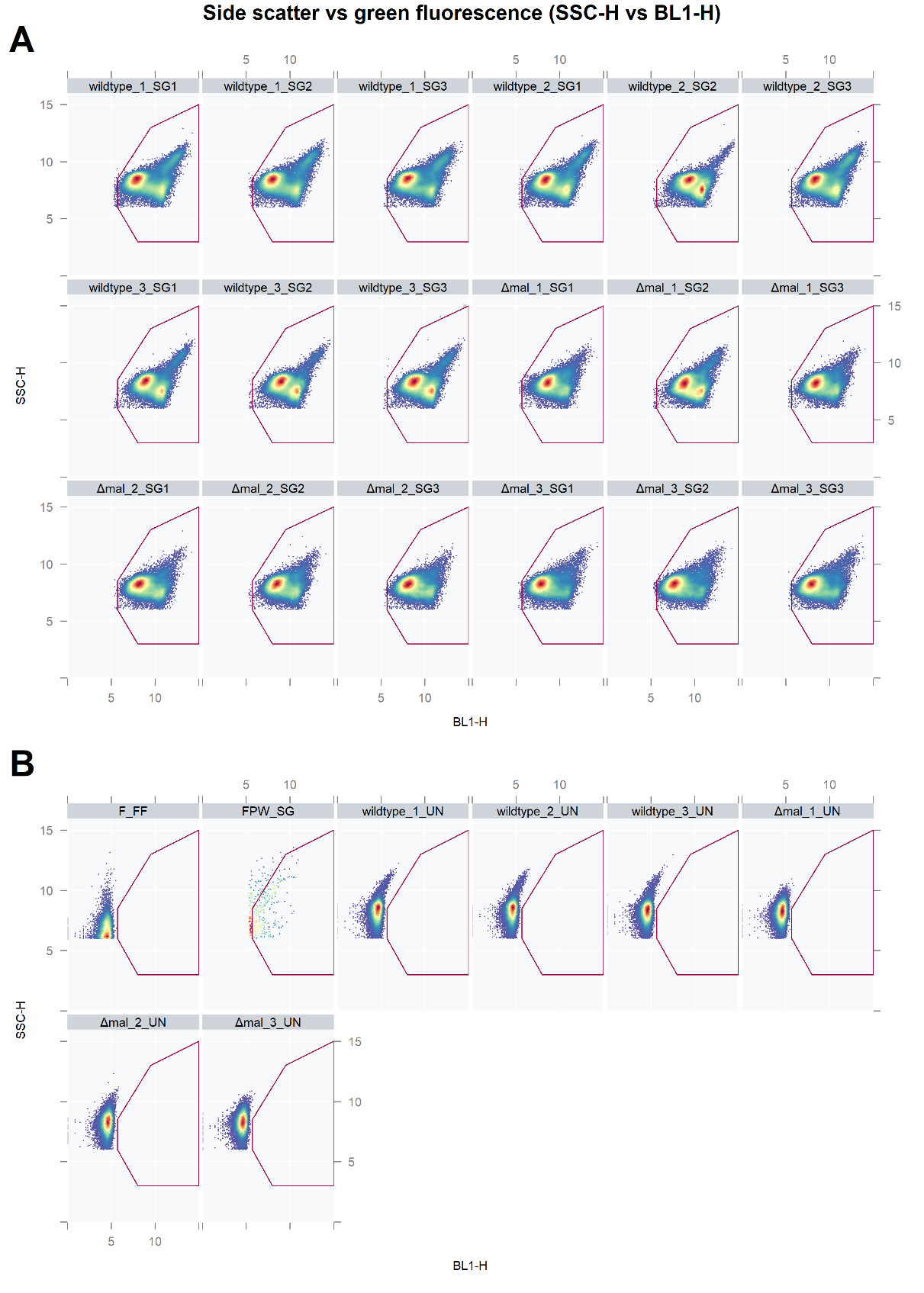


**Figure S6.1. Flow cytometry gating strategy for total cell count of *S*. *maltophilia* 44/98 treated with norfloxacin.** Side scatter (voltage height parameter; SSC-H) was plotted against SYBR Green I fluorescence detected in the blue laser primary channel (voltage height parameter; BL1-H). Flow cytometry data was transformed using the arcsine hyperbolic function and singlet gating was performed. Total bacterial cells from samples (A) were gated, where background was excluded based on negative control samples (B) which included filtered focusing fluid (F_FF), SYBR Green I-stained filtered peptone-buffered saline (FPW_SG), and unstained (_UN) biological replicates of WT and Δ*mal*.


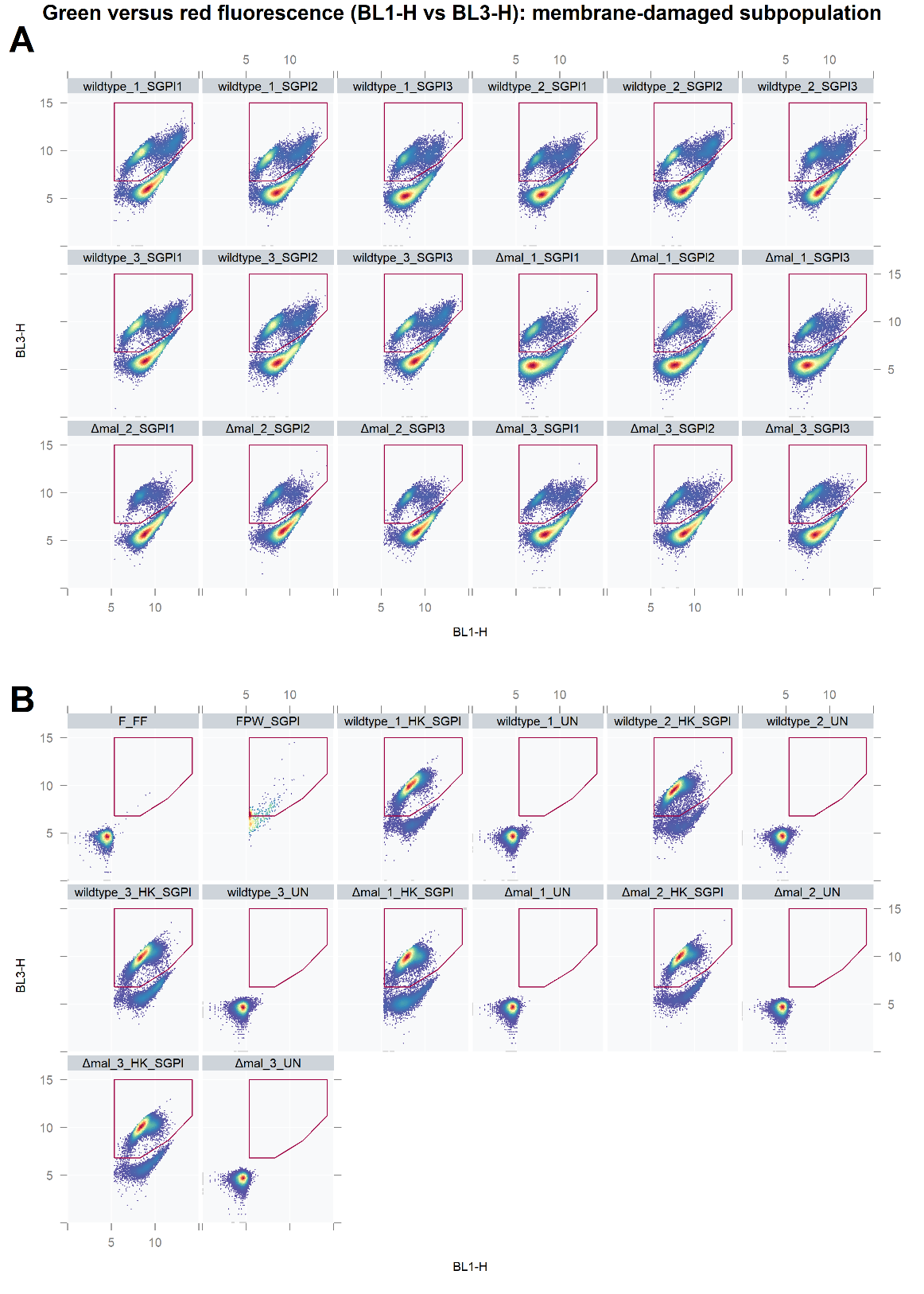
**Figure S6.2 Flow cytometry gating strategy for damaged-cell population of *S*. *maltophilia* 44/98 treated with norfloxacin.** Propidium iodide fluorescence detected in the blue laser third channel (voltage height parameter; BL3-H) was plotted against SYBR Green I fluorescence detected in the blue laser primary channel (voltage height parameter; BL1-H). Flow cytometry data was transformed using the arcsine hyperbolic function and singlet gating was performed. Cells with membrane damage (i.e., dual-stained) from samples (A) were gated. Signal from cells with intact membrane (i.e., single-stained with SYBR Green I) and background signal were excluded based on negative control samples (B) which included filtered focusing fluid (F_FF), SYBR Green I plus propidium iodide-stained filtered peptone-buffered saline (FPW_SGPI), heat-killed stained (_HK_SGPI), and unstained (_UN) biological replicates of WT and Δ*mal*.


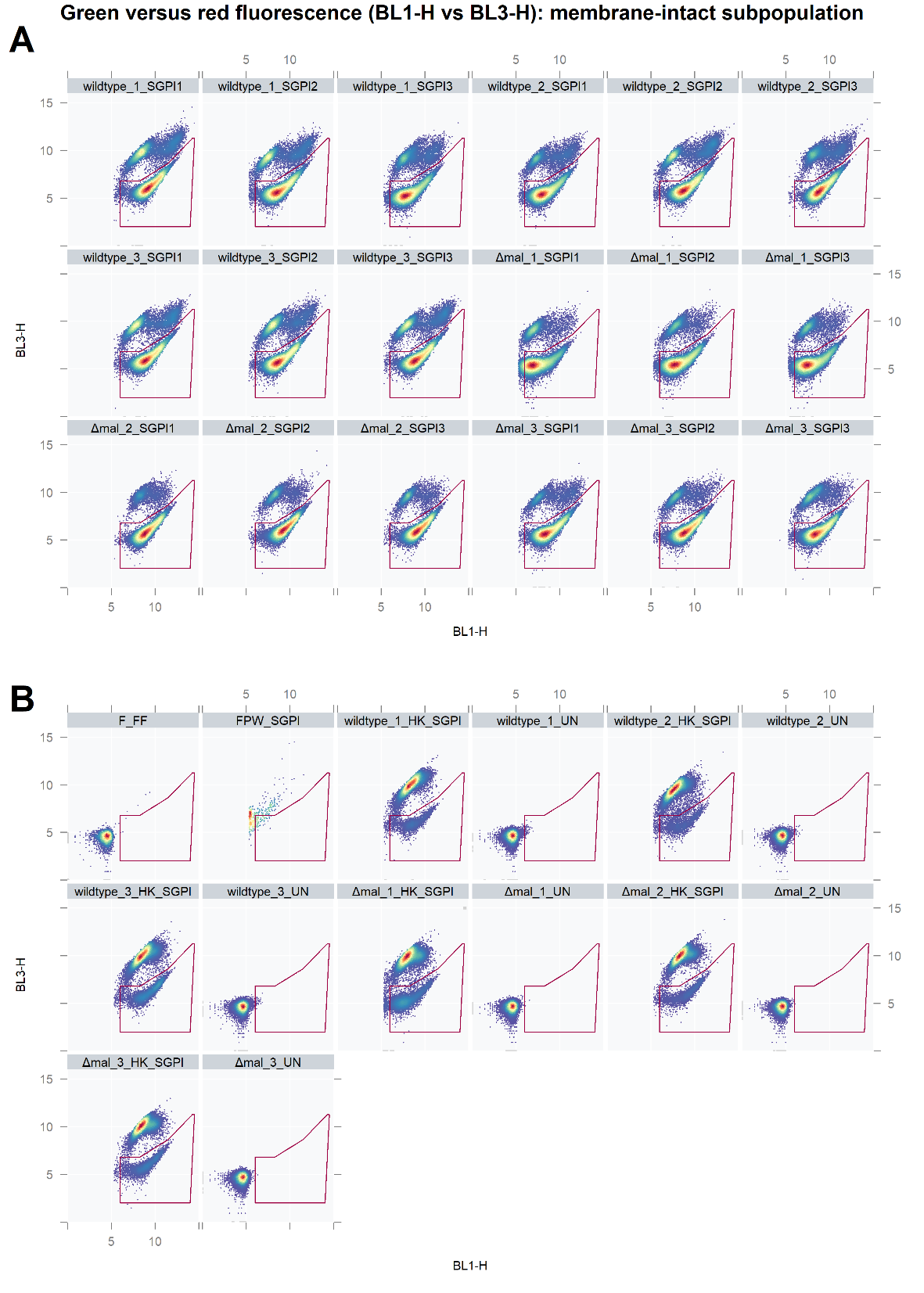
**Figure S6.3 Flow cytometry gating strategy for intact-cell population of *S. maltophilia* 44/98 treated with norfloxacin.** Propidium iodide fluorescence detected in the blue laser third channel (voltage height parameter; BL3-H) is plotted to SYBR Green I fluorescence captured in the blue laser primary channel (voltage height parameter; BL1-H). Flow cytometry data was transformed using the arcsine hyperbolic function and singlet gating was performed. Bacterial cells with intact membrane (i.e., single stained with SYBR Green I) from samples (A) were gated, signal from cell with membrane damage (i.e., dual stained) and background signal were excluded based on negative control samples (B) which included filtered focusing fluid (F_FF), SYBR Green I plus propidium iodide-stained filtered peptone-buffered saline (FPW_SGPI), heat-killed stained (_HK_SGPI), and unstained (_UN) biological replicates of WT and Δ*mal*.

**Table S6.1. Total *S*. *maltophilia* 44/98 cell counts for each replicate.** Provided as a separate Excel spreadsheet.

**Table S6.2. Summary of *S*. *maltophilia* 44/98 live/dead cell counting in response to norfloxacin.** Provided as a separate Excel spreadsheet.
