# Supplementary material for: DNA damage-associated vesicle production in Stenotrophomonas maltophilia is mediated by the maltocin endolysin
Source: J Bacteriol. 2026 Jun 25;208(7):e00158-26. doi: 10.1128/jb.00158-26 (PMC13393414; doi:10.1128/jb.00158-26)
Supplement: Supplementary Material S7 (Part 1 of 2) — Figures S7.1 to S7.6. [file jb.00158-26-s0003.docx]

**
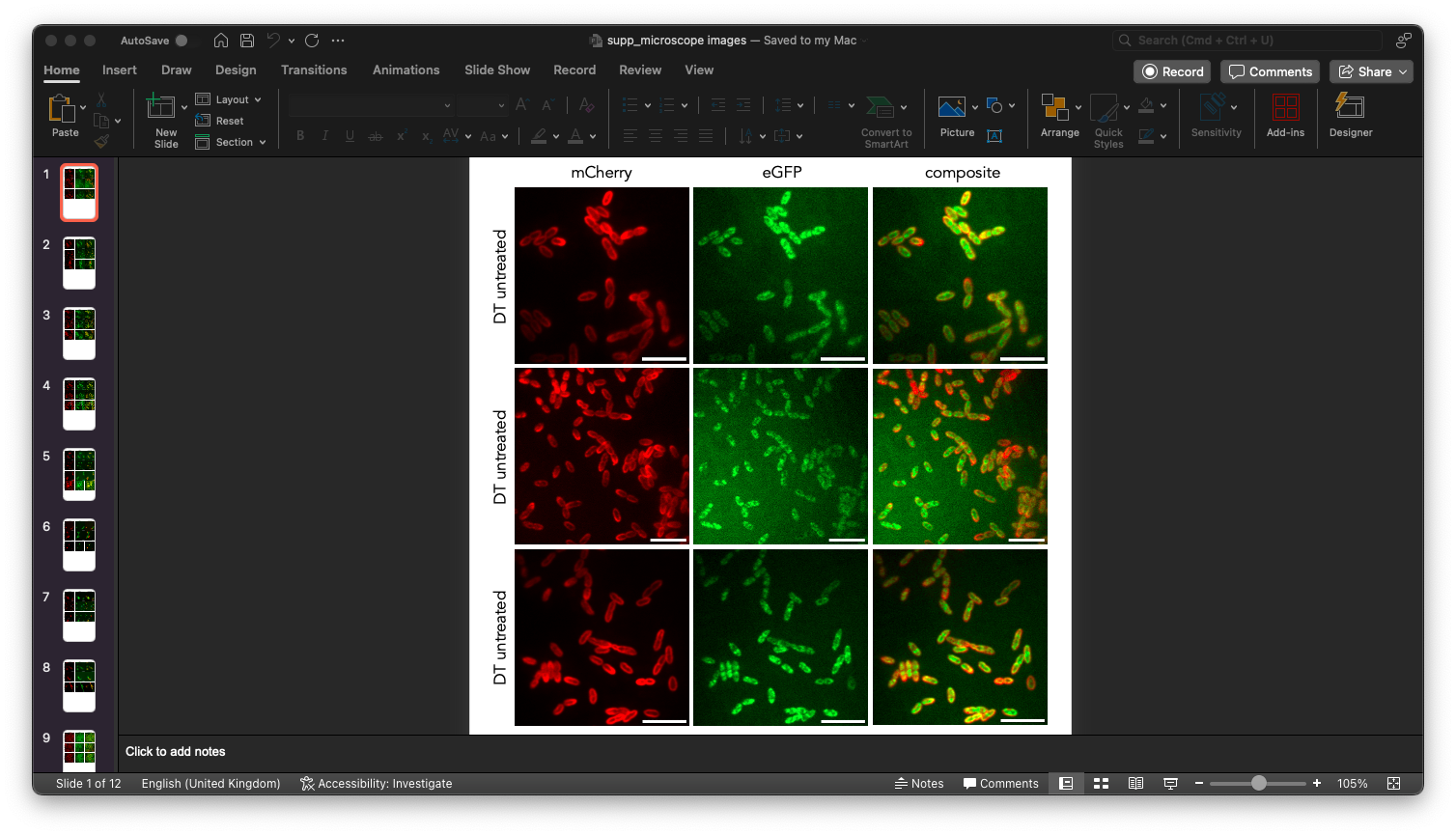
Supplementary Material 7. Additional fluorescence microscopy images**

**Figure S7.1. *S*. *maltophilia* cells under normal conditions.** When cells are not exposed to any stress-inducing compounds, the outer (represented by the mCherry-tagged Ax21) and inner (represented by the eGFP-tagged AtpG) membranes remain unaffected.

Scale bar – 5 μm.

**
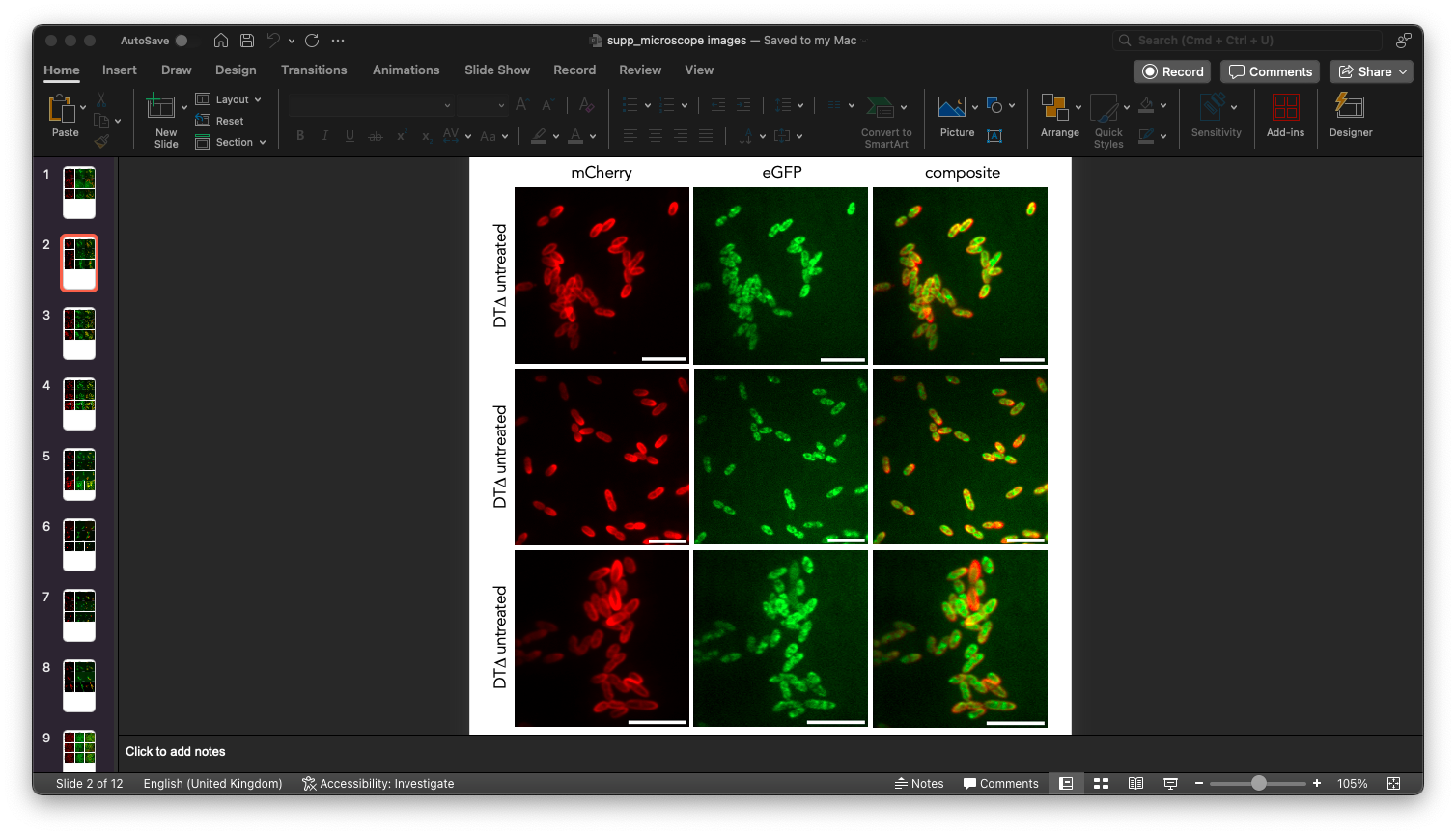
Figure S7.2. *S*. *maltophilia* Δ*mal* mutant cells under normal conditions.** Similar to wild-type cells, the outer (represented by the mCherry-tagged Ax21) and inner (represented by the eGFP-tagged AtpG) membranes remain unaffected in the absence of stressors.

Scale bar – 5 μm.


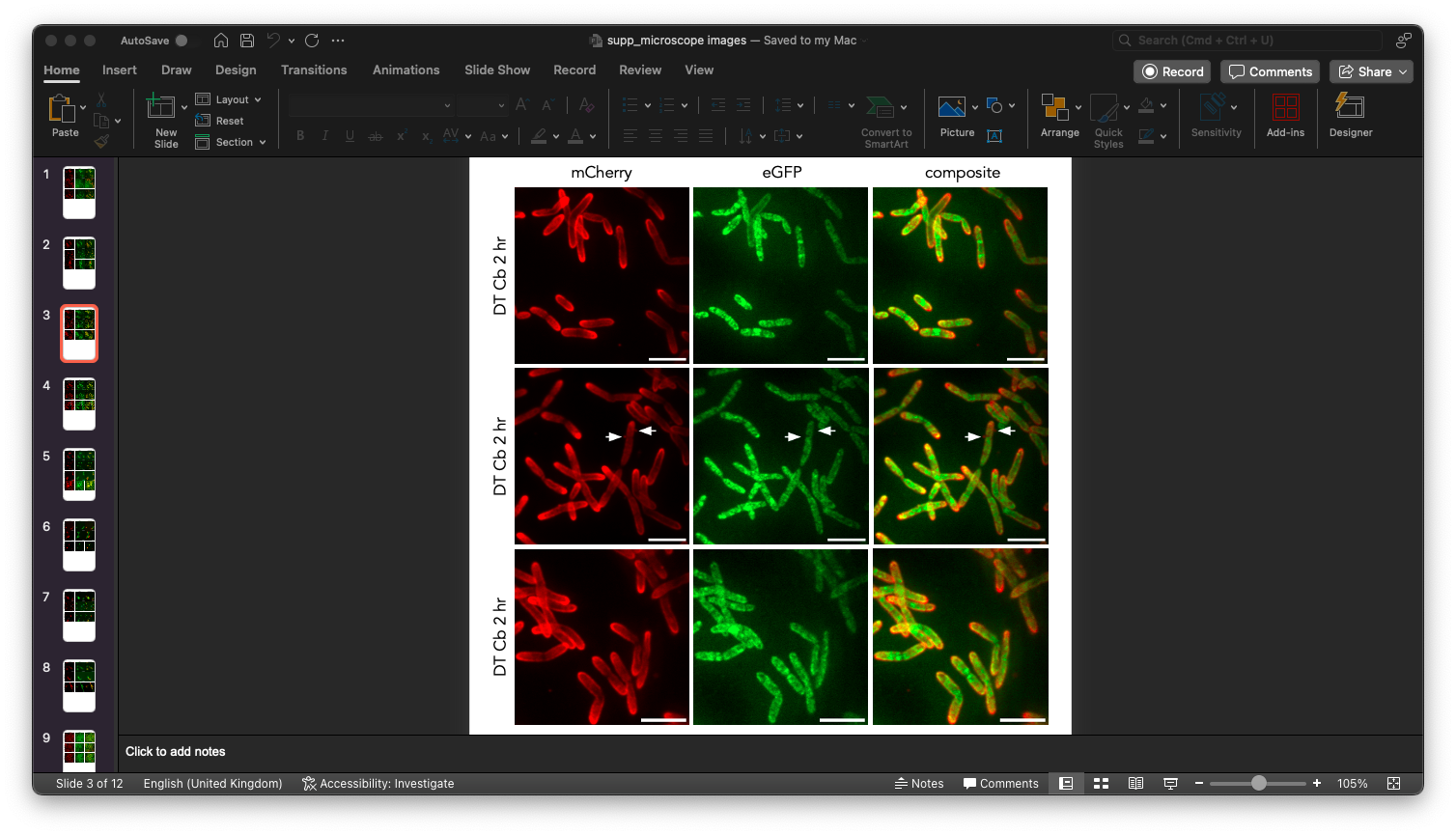
**Figure S7.3. Effect of carbenicillin on membrane integrity of *S*. *maltophilia* cells.** White arrows show OMVs with mCherry signals visible and corresponding eGFP signals absent.

Scale bar – 5 μm, Cb – carbenicillin.


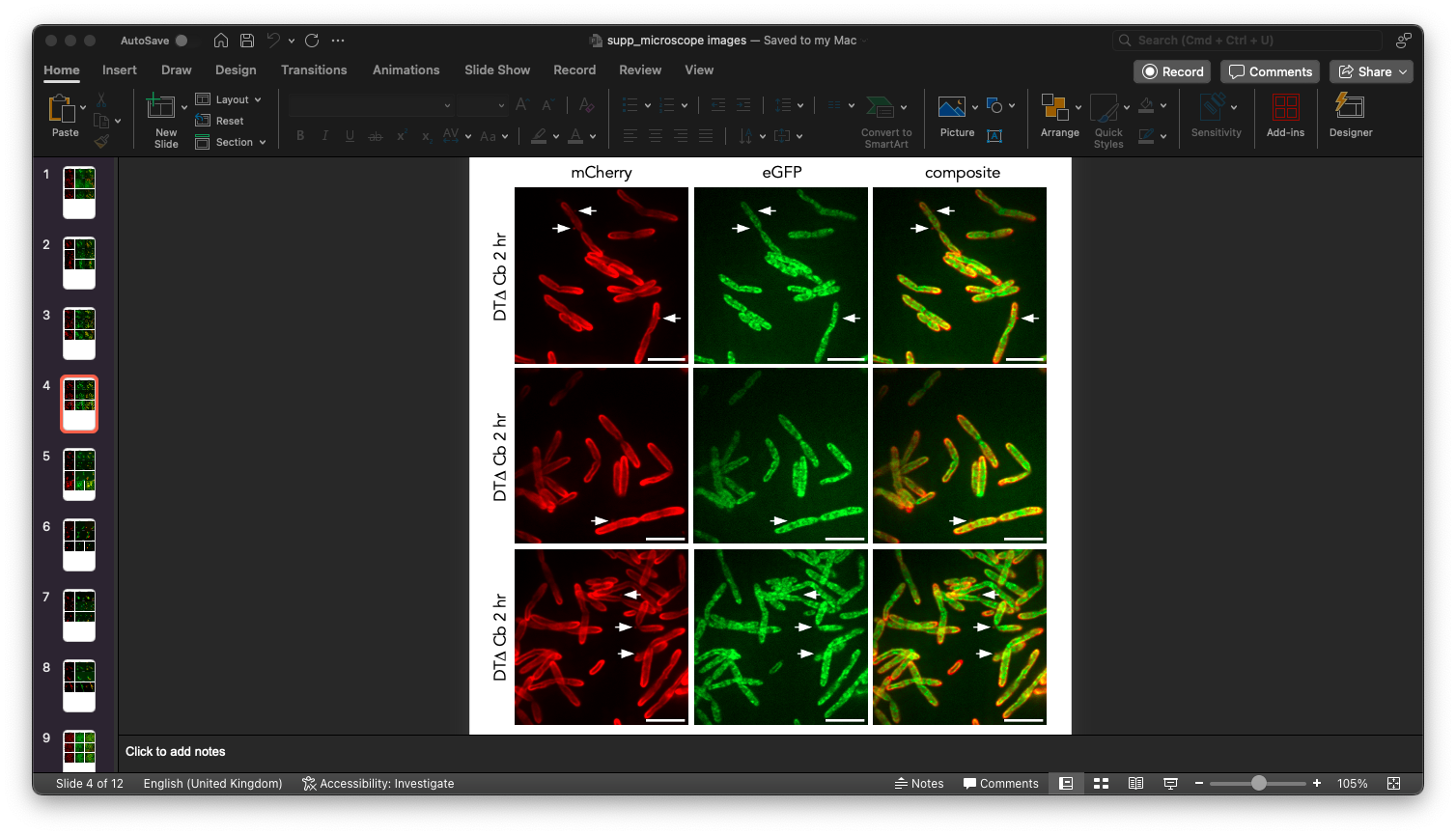
**Figure S7.4. Effect of carbenicillin on membrane integrity of *S*. *maltophilia* Δ*mal* mutant cells.** White arrows show OMVs with mCherry signals visible and corresponding eGFP signals absent.

Scale bar – 5 μm, Cb – carbenicillin.


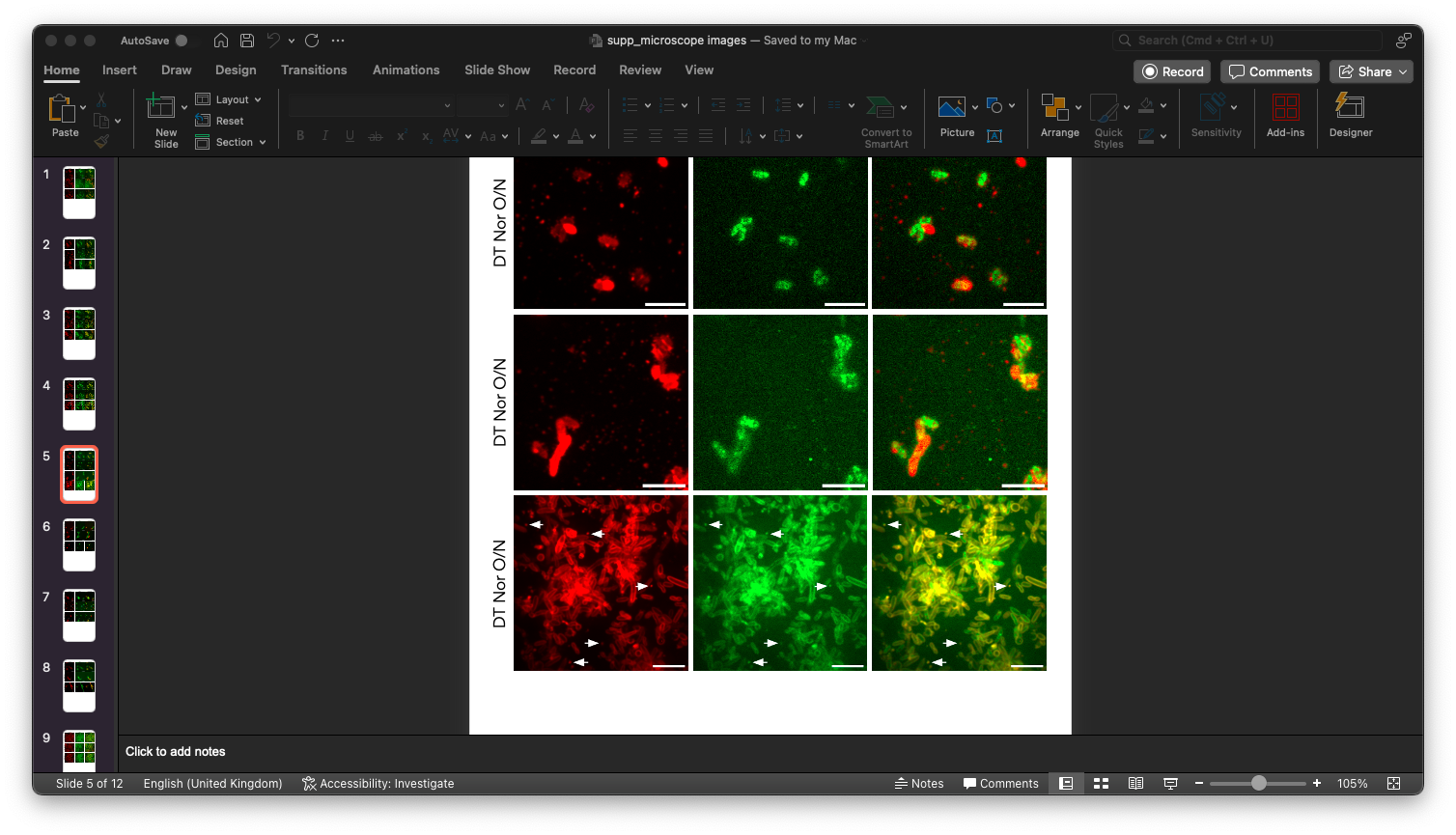
**
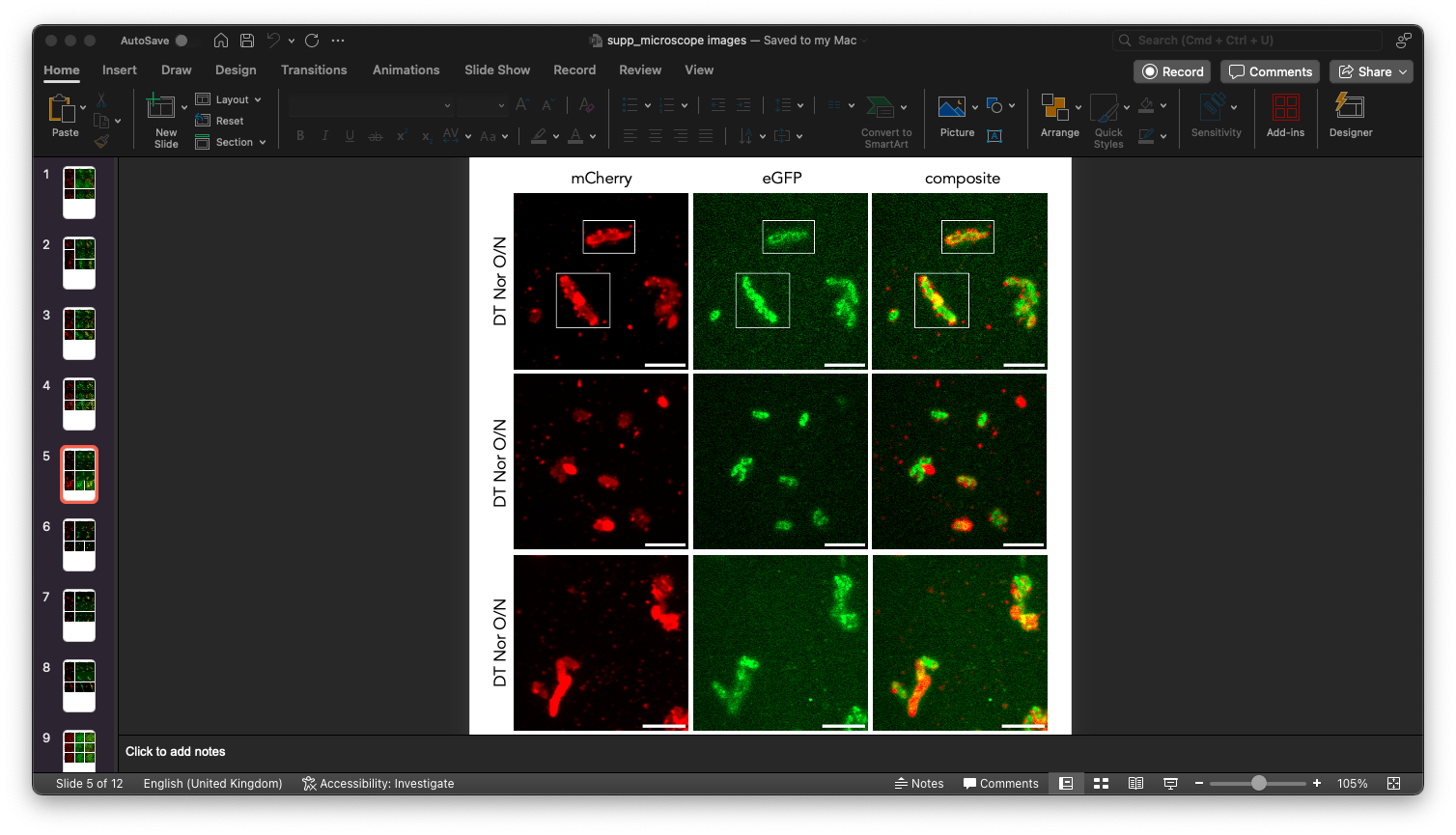
Figure S7.5. Effect of overnight norfloxacin exposure on membrane integrity of *S*. *maltophilia* cells.** Fluorescent spots separate from cells are visible in every field of view – these likely correspond to a mixture of OMVs, CMVs, and membrane fragments that have not re-circularised. White boxes show cells with bright foci at cell peripheries, indicating membrane damage. White arrows show a mix of OMVs and OIMVs with mCherry and corresponding eGFP signals.

Scale bar – 5 μm, Nor – norfloxacin.

**
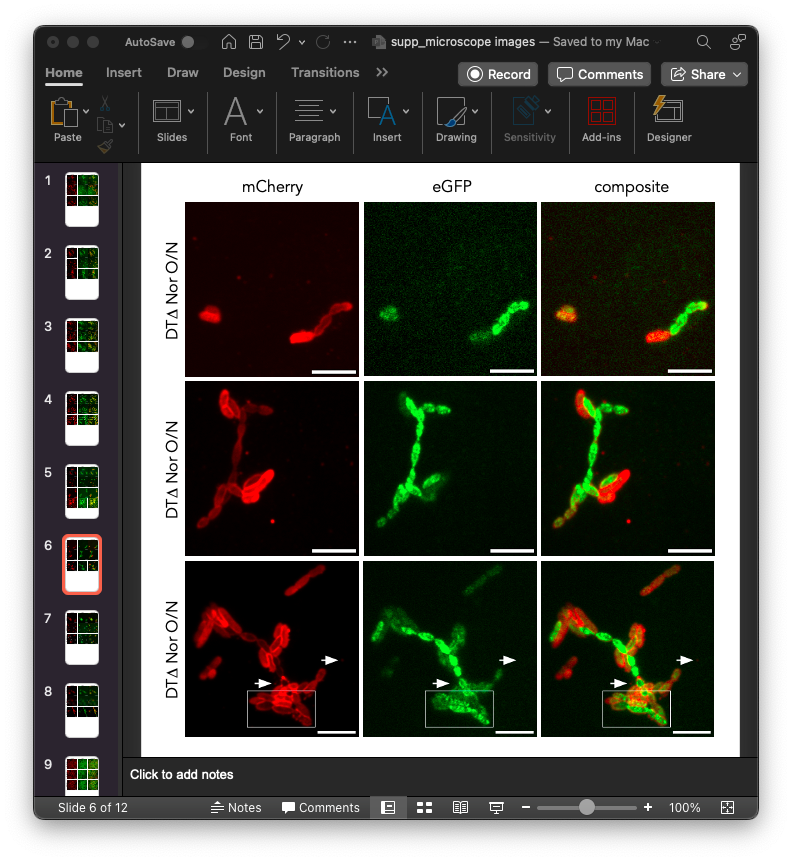
Figure S7.6. Effect of overnight norfloxacin exposure on membrane integrity of *S*. *maltophilia* Δ*mal* mutant cells.** Fluorescent spots separate from cells are visible in every field of view – these likely correspond to a mixture of OMVs, CMVs, and membrane fragments that have not re-circularised.

Scale bar – 5 μm, Nor – norfloxacin.
