# Supplementary material for: DNA damage-associated vesicle production in Stenotrophomonas maltophilia is mediated by the maltocin endolysin
Source: J Bacteriol. 2026 Jun 25;208(7):e00158-26. doi: 10.1128/jb.00158-26 (PMC13393414; doi:10.1128/jb.00158-26)
Supplement: Supplementary Material S7 (Part 2 of 2) — Figures S7.7 to S7.12. [file jb.00158-26-s0004.docx]

**Supplementary Material 7. Additional fluorescence microscopy images**


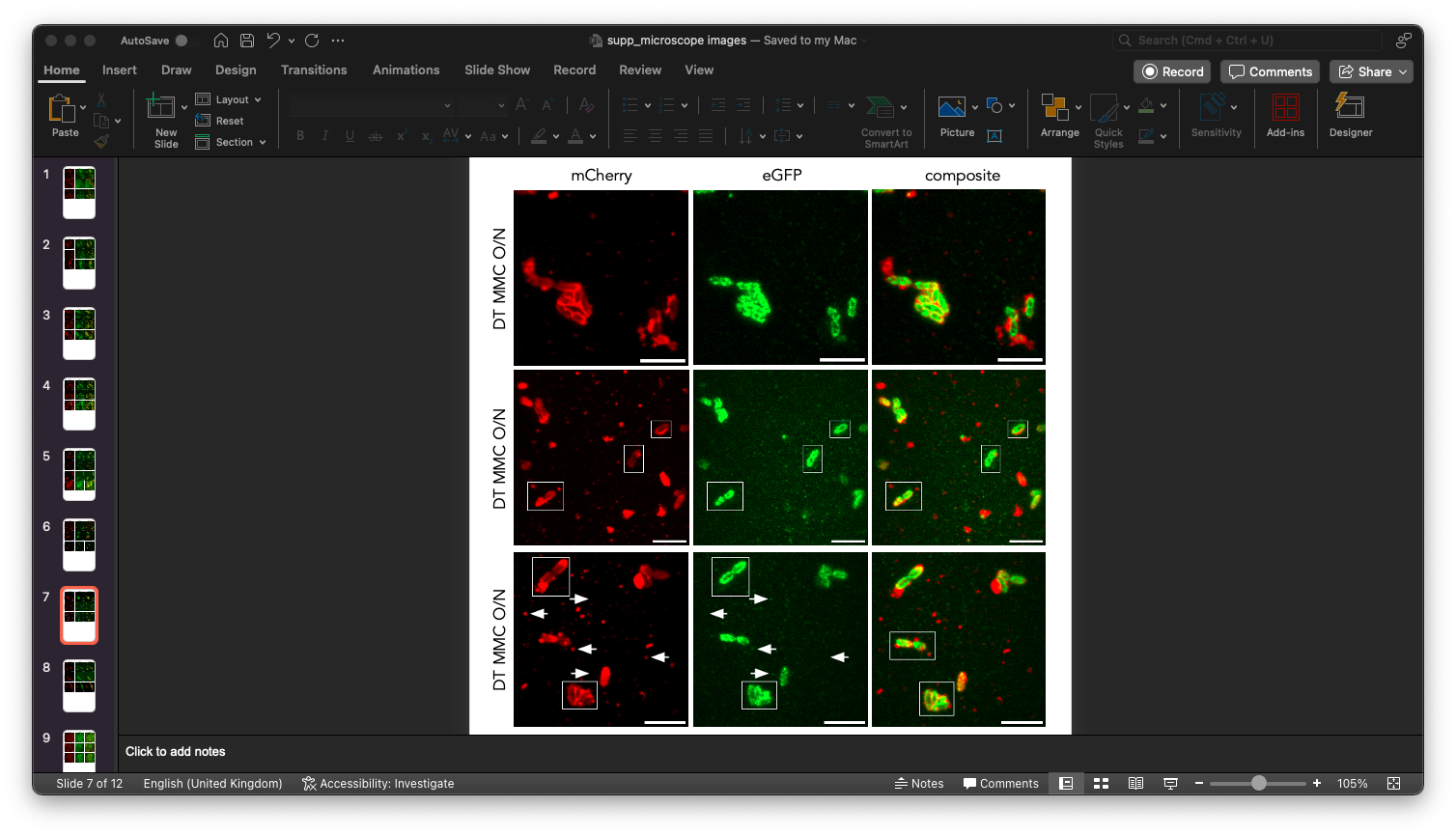
**Figure S7.7. Effect of overnight mitomycin C exposure on membrane integrity of *S*. *maltophilia* cells.** Fluorescent spots separate from cells are visible in every field of view – these likely correspond to a mixture of OMVs, CMVs, and membrane fragments that have not re-circularised. White boxes show cells with bright foci at cell peripheries, indicating membrane damage. White arrows show a very prominent OIMV with mCherry and the corresponding eGFP signals.

Scale bar – 5 μm, MMC – mitomycin C.


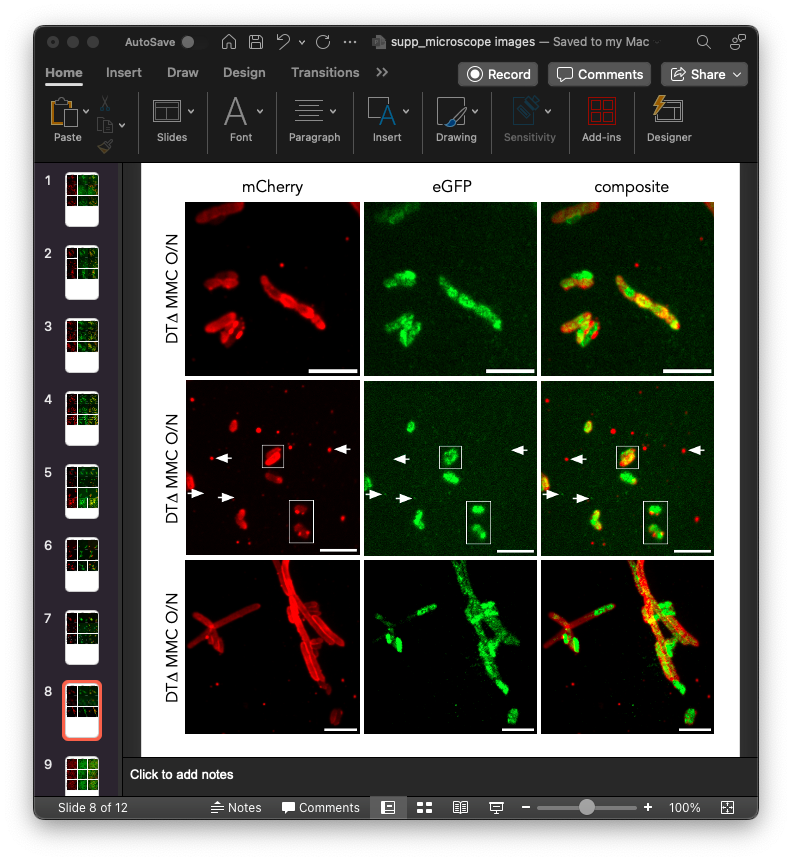
**Figure S7.8. Effect of overnight mitomycin C exposure on membrane integrity of *S*. *maltophilia* Δ*mal* mutant cells.** Fluorescent spots separate from cells are visible in every field of view – these likely correspond to a mixture of OMVs, CMVs, and membrane fragments that have not re-circularised. White boxes show cells with bright foci at cell peripheries, indicating membrane damage.

Scale bar – 5 μm, MMC – mitomycin C.

**
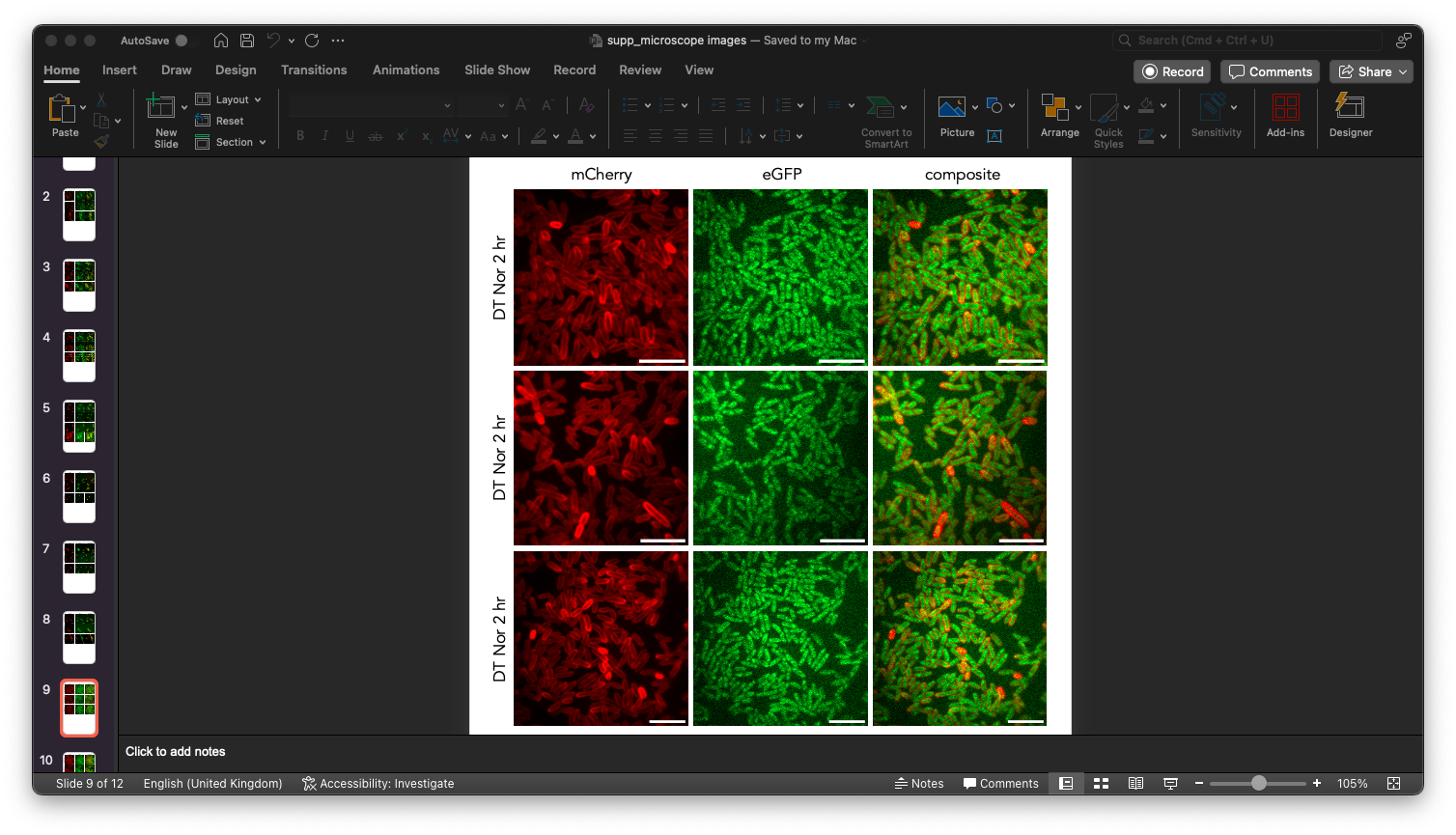
Figure S7.9. Effect of 2 hours of norfloxacin exposure on membrane integrity of *S*. *maltophilia* cells.** Re-localisation of AtpG (inner membrane) is very apparent – by contrast, localisation of Ax21 (outer membrane) remains relatively unchanged.

Scale bar – 5 μm, Nor – norfloxacin.

**
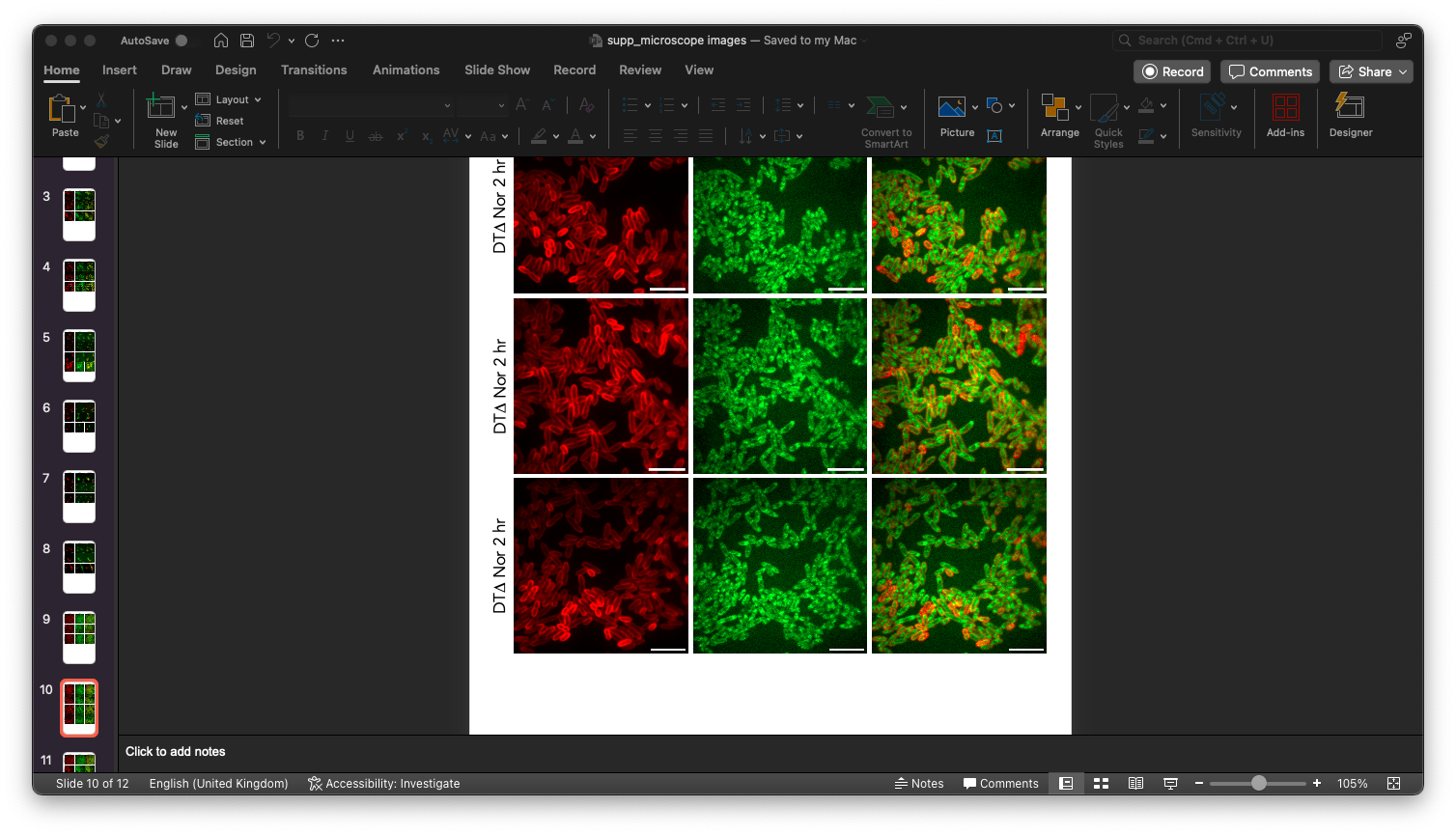

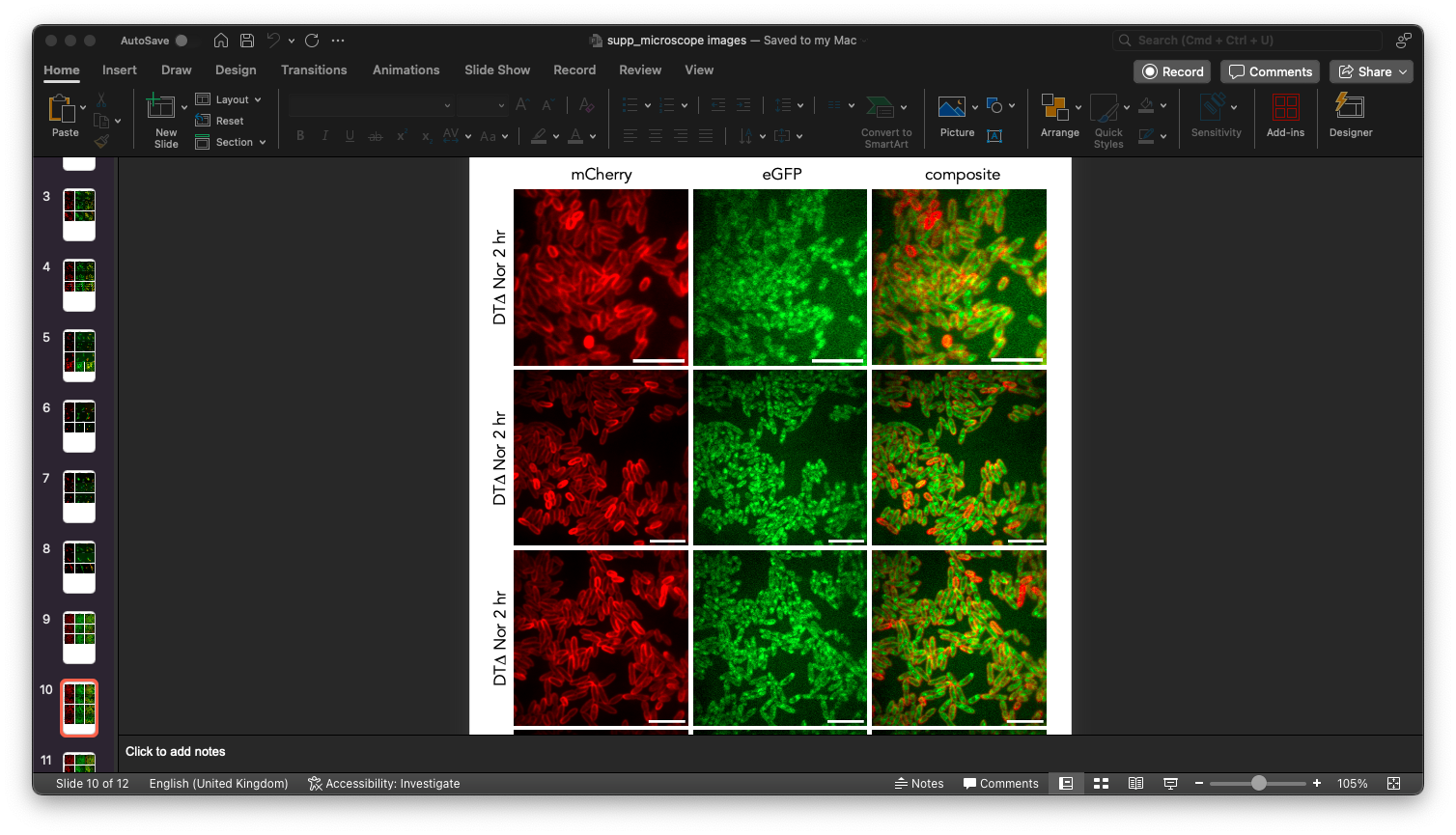
Figure S7.10. Effect of 2 hours of norfloxacin exposure on membrane integrity of *S*. *maltophilia* Δ*mal* mutant cells.** Re-localisation of AtpG (inner membrane) is very apparent – by contrast, localisation of Ax21 (outer membrane) remains relatively unchanged.

Scale bar – 5 μm, Nor – norfloxacin.

**
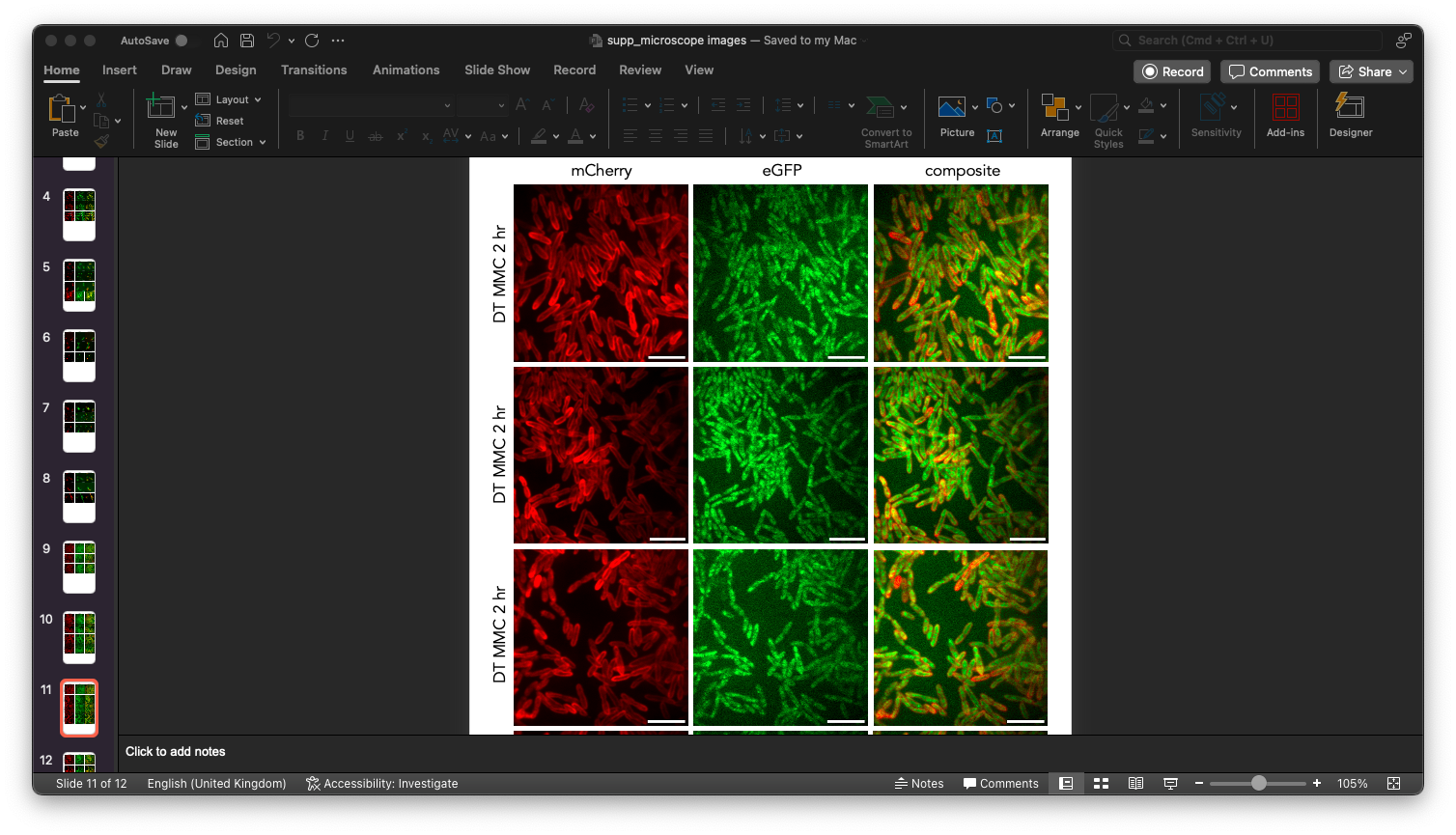
**
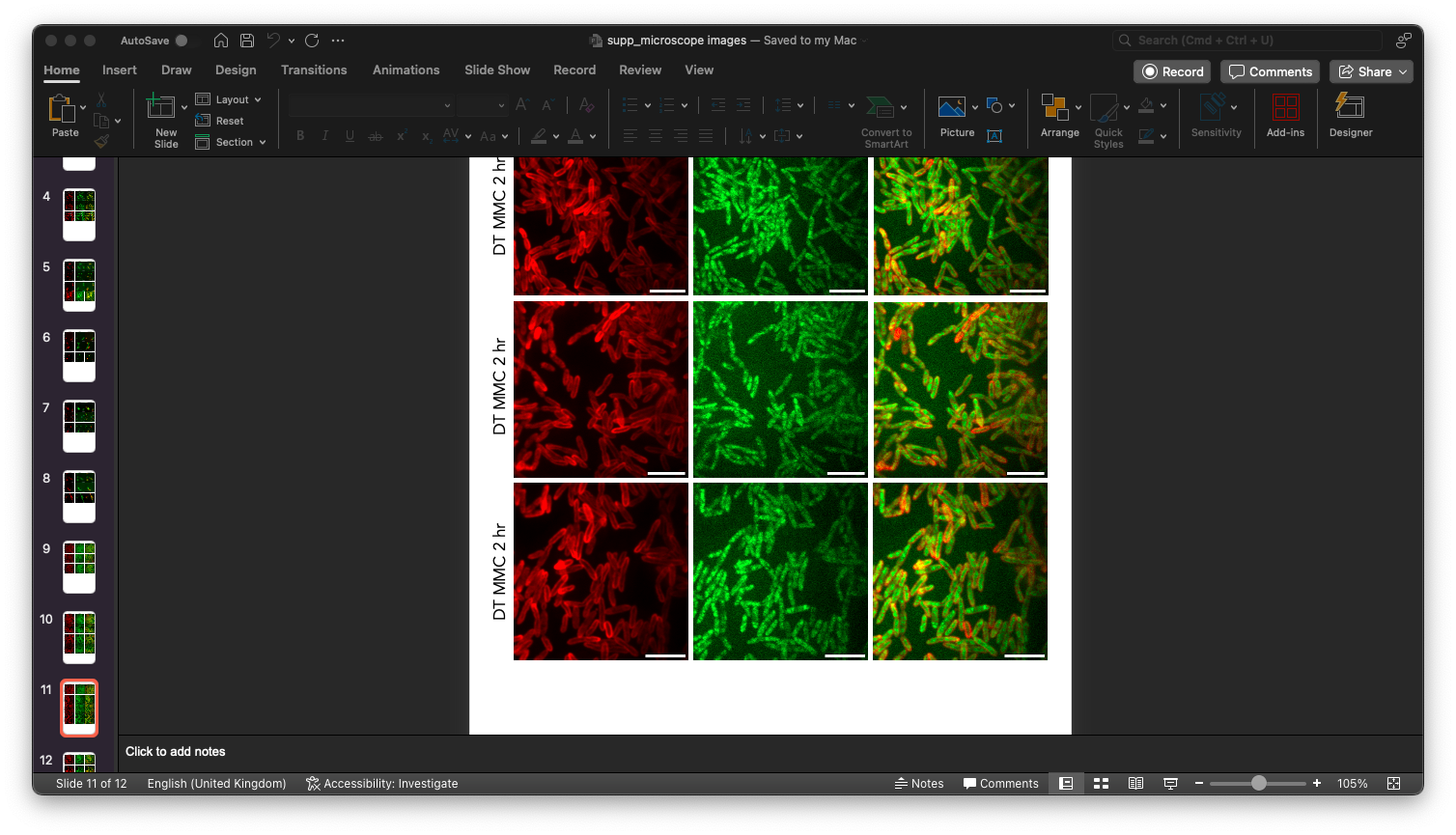
**Figure S7.11. Effect of 2 hours of mitomycin C exposure on membrane integrity of *S*. *maltophilia* cells.** Re-localisation of AtpG (inner membrane) is very apparent – by contrast, localisation of Ax21 (outer membrane) remains relatively unchanged.

Scale bar – 5 μm, MMC – mitomycin C.

**
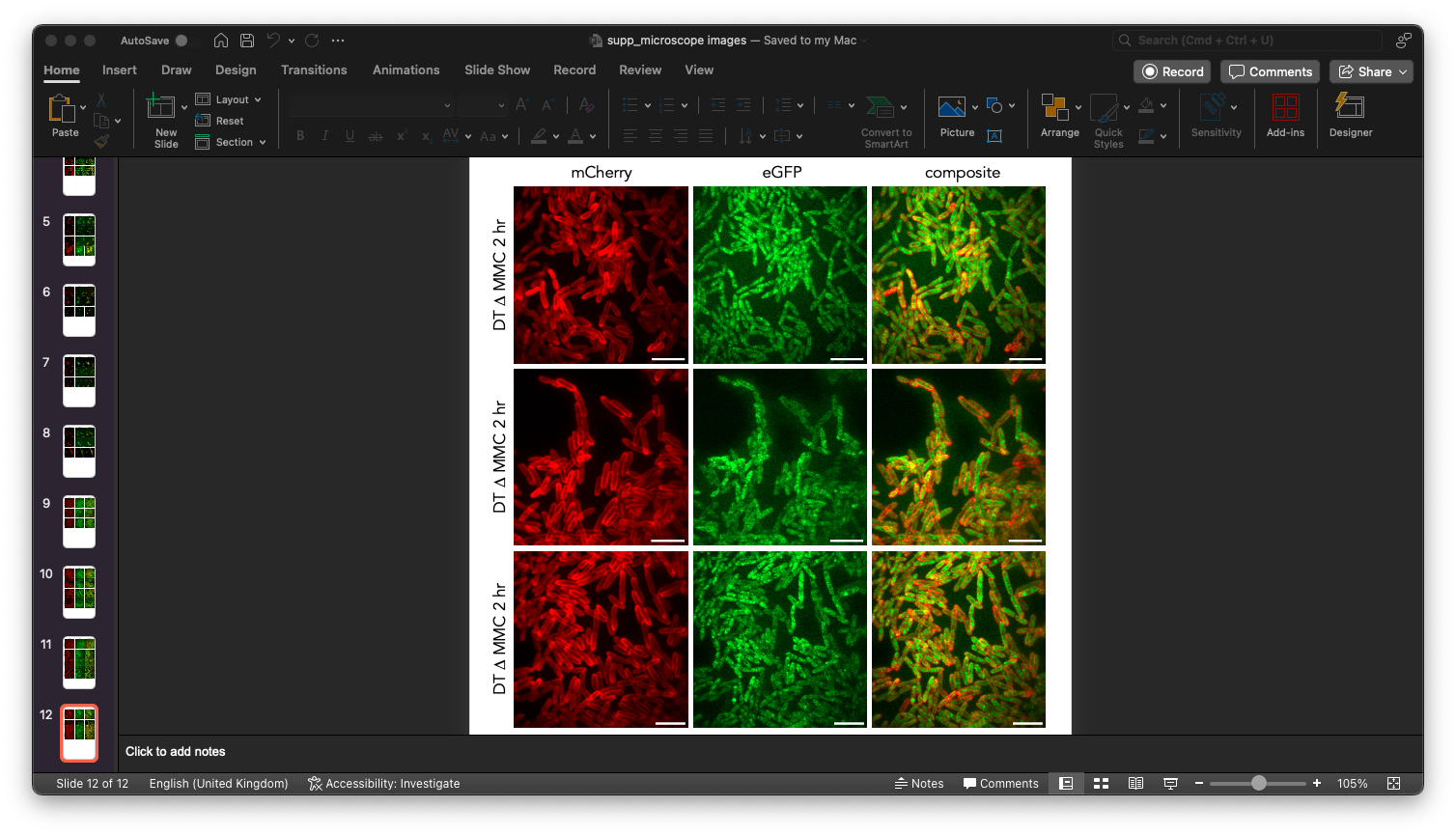
Figure S7.12. Effect of 2 hours of mitomycin C exposure on membrane integrity of *S*. *maltophilia* Δ*mal* mutant cells.** Re-localisation of AtpG (inner membrane) is very apparent – by contrast, localisation of Ax21 (outer membrane) remains relatively unchanged.
Scale bar – 5 μm, MMC – mitomycin C.
