# Supplementary material for: DNA damage-associated vesicle production in Stenotrophomonas maltophilia is mediated by the maltocin endolysin
Source: J Bacteriol. 2026 Jun 25;208(7):e00158-26. doi: 10.1128/jb.00158-26 (PMC13393414; doi:10.1128/jb.00158-26)
Supplement: Supplementary figures and material — Figure S8, Supplementary Material S9, and legends for Supplementary Movies S1 and S2. [file jb.00158-26-s0005.docx]

**Supplementary Material 8. Additional cryo EM images**


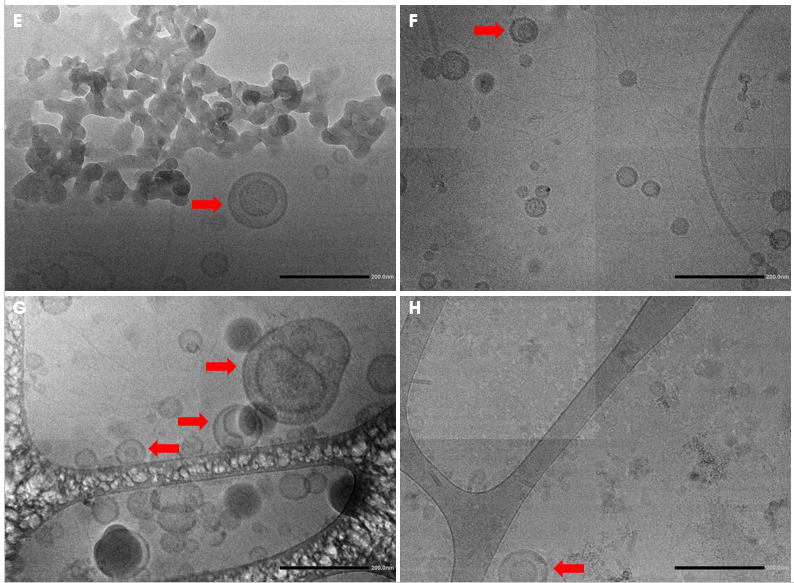


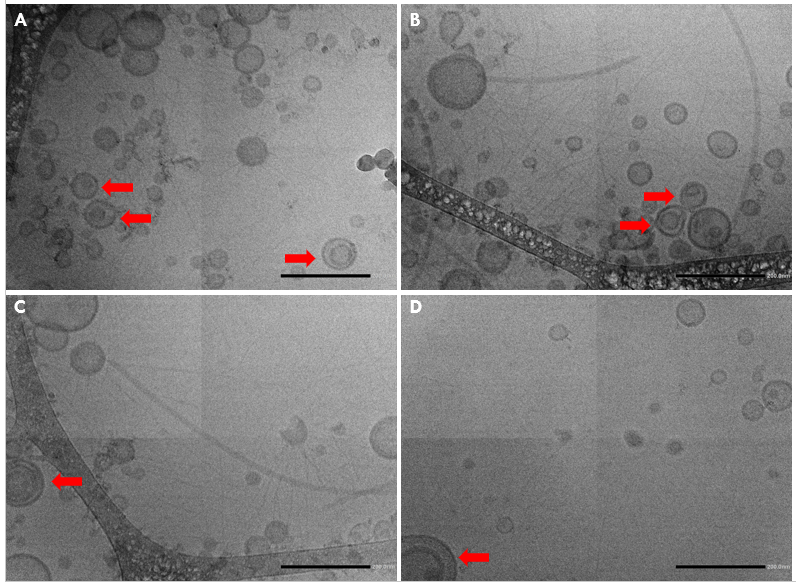


**Figure S8.1. Cryo-EM images of different samples showing prominent OIMVs.** OIMVs are marked with red arrows. (A) to (G) WT culture treated with norfloxacin or mitomycin C. (H) Δ*mal* culture treated with mitomycin C.

Scale bar – 200 nm.


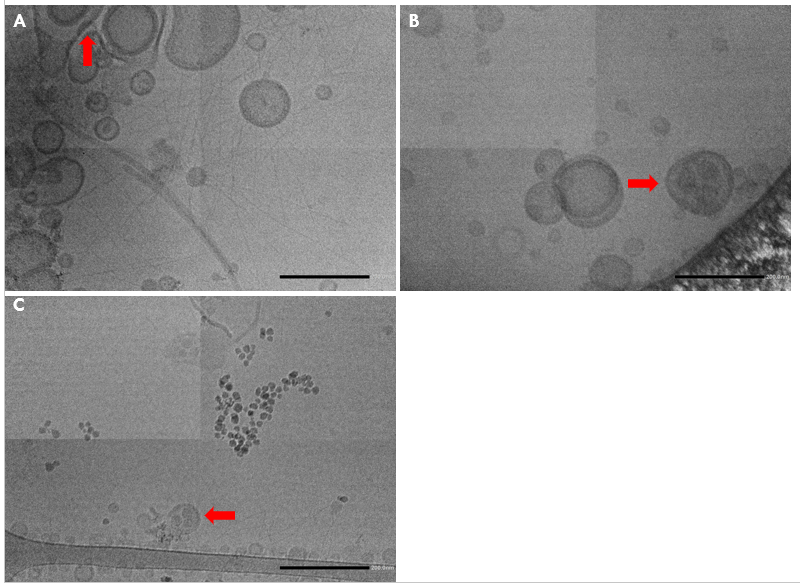
**Figure S8.2. Cryo-EM images of different samples showing atypical OIMVs.** (A) WT culture treated with mitomycin C. A rather large OIMV with multiple cytoplasmic compartments and irregular structure is visible (red arrow). (B) WT culture treated with mitomycin C. An OIMV with three cytoplasmic compartments is marked (red arrow). (C) Δ*mal* culture treated with mitomycin C. A smaller OIMV with three cytoplasmic compartments is marked (red arrow).

Scale bar – 200 nm.


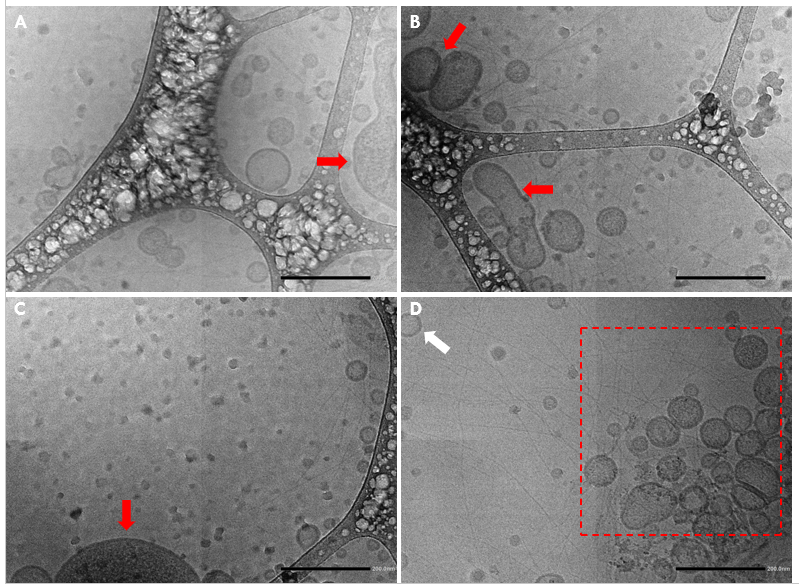

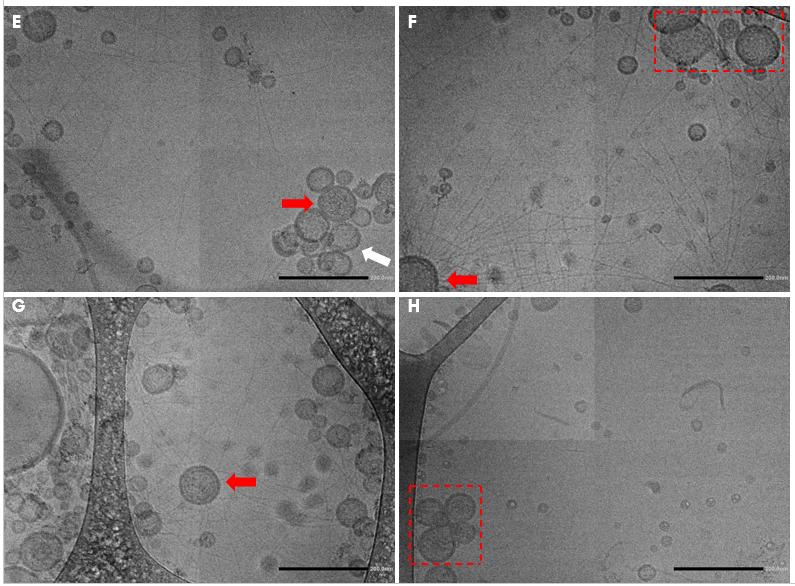
**Figure S8.3. Cryo-EM images of different samples showing possible CMVs or E-OMVs.** MVs with darker staining profiles (likely indicating cytoplasmic cargo) were found across all samples, predominantly those from the WT (red arrows and boxes). These were found to differ from the bulk of single-layered MVs with lighter staining patterns (white arrow). The darker MVs were usually found to occur in groups of 2–4 and sometimes associated with several filamentous particles, while some were found to have large and irregular morphologies. (A) to (G) WT culture treated with norfloxacin or mitomycin C. (H) Δ*mal* culture treated with norfloxacin.

Scale bar – 200 nm.

**Supplementary Material 9. Sequences of homology regions and inserts used**

**Δ*mal* and *mal:egfp***

Upstream homology sequence

CGCGCCTGCGGCGCTGTTGGCGAGGGCCTGCTGGTGCGCGCGCTGGACTGCATCGGTGCGGGCCTCCTGCTGCGCTGCCCGGGTGGTGGCAACGGCAAGGTCCGCAGTGCGGTCGCGCAGGGTCCATCCCAGCCAGGCGCAGCTCGCGTGGCTACCGGCAAGGACCAGCAACCCGATGCCCAACCTCAGTCCCGCGGGCG

Downstream homology sequence

CTGCAGGGCTCCGCCGGCGGCACGGTACGCCGCGCGCAGTGTTTCCAGTGCGTGCTCCTTCTGGCCGTAGCCGGCGCCCGGCAGCGATGCCCAGATGCGCCGGGCCGCGGTGACGGCGGCATCGAAGCGTCCCAGCCGGATCAGCTCGTAGGCACCGCACTGTTTCAGCAGGGCGACTGCTGCACGGTCCTGGGAGACTGGCCCGAAGTCAGGCAGACCCAGTCGCGCGCGCAGGTCATCCCAGGTGCTGCGCAGGAACTGGTAGCGGCCGGCGGCGCTGGATTTGATGCCATAGCGTGG

Linker + eGFP sequence (reverse complement)

TTACTTGTACAGCTCGTCCATGCCGAGAGTGATCCCGGCGGCGGTCACGAACTCCAGCAGGACCATGTGATCGCGCTTCTCGTTGGGGTCTTTGCTCAGGGCGGACTGGGTGCTCAGGTAGTGGTTGTCGGGCAGCAGCACGGGGCCGTCGCCGATGGGGGTGTTCTGCTGGTAGTGGTCGGCGAGCTGCACGCTGCCGTCCTCGATGTTGTGGCGGATCTTGAAGTTCACCTTGATGCCGTTCTTCTGCTTGTCGGCCATGATATAGACGTTGTGGCTGTTGTAGTTGTACTCCAGCTTGTGCCCCAGGATGTTGCCGTCCTCCTTGAAGTCGATGCCCTTCAGCTCGATGCGGTTCACCAGGGTGTCGCCCTCGAACTTCACCTCGGCGCGGGTCTTGTAGTTGCCGTCGTCCTTGAAGAAGATGGTGCGCTCCTGGACGTAGCCTTCGGGCATGGCGGACTTGAAGAAGTCGTGCTGCTTCATGTGGTCGGGGTAGCGGCTGAAGCACTGCACGCCGTAGGTCAGGGTGGTCACGAGGGTGGGCCAGGGCACGGGCAGCTTGCCGGTGGTGCAGATGAACTTCAGGGTCAGCTTGCCGTAGGTGGCATCGCCCTCGCCCTCGCCGGACACGCTGAACTTGTGGCCGTTTACGTCGCCGTCCAGCTCGACCAGGATGGGCACCACCCCGGTGAACAGCTCCTCGCCCTTGCTCACCATGCTGCCGCTGCCGCTGCC

***atpG:egfp***

Upstream homology sequence

ACGGCATGCGGCCCAGCAGTGCCGACACTTCGGTACCGGCCAGGGTGTAGCGGTAGATGTTGTCGACGAACAGCAGGACGTCCTTGCCCTTGCCGTTTTCGTCCTTCTCGTCGCGGAAGTACTCGGCCATGGTCAGGCCGGTCAGGGCGACGCGCAGACGGTTGCCCGGCGGCTCGTTCATCTGGCCGTACACCATCGCCACCTTGTCGAGGACGTTGGAGTCCTTCATTTCGTGGTAGAAGTCGTTGCCCTCACGGGTACGCTCACCCACGCCGGCGAACACGGACAGACCGCTGTGCGCCTTGGCGATGTTGTTGATCAGCTCCATCATGTTGACGGTCTTGCCGACGCCGGCGCCGCCGAACAGGCCGACCTTGCCGCCCTTGGCGAACGGGCACATCAGGTCGATGACCTTGATGCCGGTTTCCAGCAGTTCGGTGGCCGGGGACTGGTCTTCGTACGACGGGGCCGCACGGTGGATTTCCCAGCTGTCGCTGGCGGCCACCGGGCCGGCTTCGTCGATCGGACGGCCGAGCACGTCCATGATGCGGCCCAGGGTGCCGGCGCCGACCGGCACCGAGATGCCACGGCCGGTGTTGACGGCAACCAGGTTGCGCTTCAGGCCGTCGGTGGAACCGAGGGCGATGGTACGCACCACGCCGTCGCCCAGCTGCTGCTGGACTTCGAGGGTGATCTCGGTGTTTTCCACCTTCAGTGCGTCGTACACCTTCGGCACCGATTCACGCGGGAATTCGACGTCGACGACCGCGCCGATGATCTGAACGATCTTGCCCTGACTCATTGCTGCATCCTCTAATGTGTGCTTTGAACGAACGCGG

Downstream homology sequence

GACTGCTGCCGCGCCGCCGACGATTTCGGAGATTTCCTGGGTGATCGCTGCCTGGCGCGCCTTGTTGTAGACGAGCTGCAGGGTGCCGATCAGCTTGTTGGCGTTGTCGCTCGCCGCCTTCATCGCAACCATGCGTGCCGCATGCTCGGAGGCGACGTTTTCCAGCAGTGCCTGGTACACCAGCGACTCGATGTAACGCGTCATCACGTGCTCGAGCACGGTCGCGGCATCGGGTTCGTACAGGTAATCCCAGTCGTGGTGAGCGACCTGCTTCTCAGCCGGCGGCAGCGGCAGCAGCTGGTCGAAGCTGGCCTTCTGCACCATGGTGTTCACGAAGCGGTTGTAGACCAGGTACACGCGGTCGATCTTGCCTTCGGTGAAGGCGTCGAGCATGACCTTGATCACACCGATCAGCGATTCCAGCTTCGGCACATCGCCGATGTGGGTCACGCTGCCGACCATGTTGACCTTCACGCGGCGGAAGAAGGTCGAAGCCTTCTGGCCGATGGTCACCAGGTCCACTTCCGCACCCTTGTCCTGCCATGCCTTGGCTTCGCCCAGCATCTTGCGGAACAGGTTGTTGTTCAGGCCGCCGGCCAGGCCGCGATCGGAGGAGATCACGATGAAGCCGACCCGCTTGACCTGCTCGCGCTCGACCAGGAACGGATGCTGGTAGTCGGTGCTGGCCTGGGCCAGGTGACCGATCACCTGCTTCATCGCCTGCGCGTACGGACGCGAGGTCTTCATCCGATCCTGCGCCTTGCGGATCTTGGAGGCCGAGACCATTTCCAGGGCGCGCGTCACCTTGCGGGTGTTCTGCACGCTCTTGATCTTGGTTTTGATTTCGCGTCCGCTTGCCAT

Linker + eGFP sequence (reverse complement)

TTACTTGTACAGCTCGTCCATGCCGAGAGTGATCCCGGCGGCGGTCACGAACTCCAGCAGGACCATGTGATCGCGCTTCTCGTTGGGGTCTTTGCTCAGGGCGGACTGGGTGCTCAGGTAGTGGTTGTCGGGCAGCAGCACGGGGCCGTCGCCGATGGGGGTGTTCTGCTGGTAGTGGTCGGCGAGCTGCACGCTGCCGTCCTCGATGTTGTGGCGGATCTTGAAGTTCACCTTGATGCCGTTCTTCTGCTTGTCGGCCATGATATAGACGTTGTGGCTGTTGTAGTTGTACTCCAGCTTGTGCCCCAGGATGTTGCCGTCCTCCTTGAAGTCGATGCCCTTCAGCTCGATGCGGTTCACCAGGGTGTCGCCCTCGAACTTCACCTCGGCGCGGGTCTTGTAGTTGCCGTCGTCCTTGAAGAAGATGGTGCGCTCCTGGACGTAGCCTTCGGGCATGGCGGACTTGAAGAAGTCGTGCTGCTTCATGTGGTCGGGGTAGCGGCTGAAGCACTGCACGCCGTAGGTCAGGGTGGTCACGAGGGTGGGCCAGGGCACGGGCAGCTTGCCGGTGGTGCAGATGAACTTCAGGGTCAGCTTGCCGTAGGTGGCATCGCCCTCGCCCTCGCCGGACACGCTGAACTTGTGGCCGTTTACGTCGCCGTCCAGCTCGACCAGGATGGGCACCACCCCGGTGAACAGCTCCTCGCCCTTGCTCACCATGCTGCCGCTGCCGCTGCC

***ax21:mCherry***

Upstream homology sequence

CACGCGTTCGGCGCGCTGGTAGTCGGCCGCCTGCAGCGCGTGTGCGGTGTGTGGCGACAAGAGGGCAACGCATGCAAAGACAGCGGCGCGGACACACGTCCGCAGCGGATCGGAAACGAGCAAGGCAACCTCCTGTTGCAATCGTCGAAGGATTACAGATGCACGCGCGCCGCGCGCGTATGCCAGACGGAGCCTGAATCAAGCCCAAAAATTCAGCAATGCGAAGACCACTGTTTTCAGGGAGCGTTGTGGCGAATAACGGAGAATTTACATTCCCGATGCGATGGCGCCCTGGCGCTGCTGCACACCATAAGAAAGTAAAGGTGTACCCCCATGAAGAATTCGCTGATTGCTCTGGCCCTGGCCGCTGCCCTGCCGTTCACCGCTTCGGCT

Downstream homology sequence

TTGCTTCCTCGGCGCGCCTGCGCGCTGAAGATGCCTCGACAGGCCCGGCCCATGCCGGGCCTGTTGCGTTTCGGGCTGGGCAAACGCTACCTGACCGTGTTGAGGTGGACGGGCGGCATTGCAACGAATTGGATTCCGCAGGCGTCGGCTGCGCCCTAGGGTGAGTGGCCTGTCCTGCAGGAATGCAACGATGACCCCGTTTCCCTCGCCCCGCGATTCTCCCTGCCGCTTGTTGCTGCTGGATCCGCATCCACTGTTGCGGCATGGCGTGCAGGAACTGCTGGCCCGGCAGGCGGGCCTGCAGATCGATGGAAGCTATGGCCGCAGCGGTGATCTGCTGCAGCGCCTGCAGCAGCAGGCAGCTGCGATCGATCTGCTGCTGGTTGACGCCTTGCCGATCGGTGACAGCGGGGAAGGCCTGGAACTGCTGCGGCACGTGTCACGACATTGGCCGGCAGTGCCGATCCTGGTGCTGTCCGCACACTGCAACGCGGGTGTCG

Replacement gene

ATGAAGAATTCGCTGATTGCTCTGGCCCTGGCCGCTGCCCTGCCGTTCACCGCTTCGGCTATGGTGAGCAAGGGCGAGGAGGATAACATGGCCATCATCAAGGAGTTCATGCGCTTCAAGGTGCACATGGAGGGCTCCGTGAACGGCCACGAGTTCGAGATCGAGGGCGAGGGCGAGGGCCGCCCCTACGAGGGCACCCAGACCGCCAAGCTGAAGGTGACCAAGGGTGGCCCCCTGCCCTTCGCCTGGGACATCCTGTCCCCTCAGTTCATGTACGGCTCCAAGGCCTACGTGAAGCACCCCGCCGACATCCCCGACTACTTGAAGCTGTCCTTCCCCGAGGGCTTCAAGTGGGAGCGCGTGATGAACTTCGAGGACGGCGGCGTGGTGACCGTGACCCAGGACTCCTCCCTGCAGGACGGCGAGTTCATCTACAAGGTGAAGCTGCGCGGCACCAACTTCCCCTCCGACGGCCCCGTAATGCAGAAGAAGACCATGGGCTGGGAGGCCTCCTCCGAGCGGATGTACCCCGAGGACGGCGCCCTGAAGGGCGAGATCAAGCAGAGGCTGAAGCTGAAGGACGGCGGCCACTACGACGCTGAGGTCAAGACCACCTACAAGGCCAAGAAGCCCGTGCAGCTGCCCGGCGCCTACAACGTCAACATCAAGTTGGACATCACCTCCCACAACGAGGACTACACCATCGTGGAACAGTACGAACGCGCCGAGGGCCGCCACTCCACCGGCGGCATGGACGAGCTGTACAAGGGCAGCGGCAGCGGCAGCGCTGAGAACCTGTCGTACAACTACGCTGAAGCCGACTACGCCAAGACCGACGTCGATGGCATCAAGGCTGACGGCTGGGGCGTCAAGGGTTCCTACGGCTTCCTGCCGAACTTCCACGCGTTCGGTGAATACAGCCGTCAGGAAGTCGACCACACCAACATCAAGGTTGACCAGTGGAAGGTCGGTGCCGGCTACAACGTCGAAATCGCTCCGTCGACCGACTTCGTTGCCCGCGTTGCCTACCAGAAGTTCGATCGCAAGCACGGCCTGGACTTCAACGGCTACAGCGCTGAAGCCGGTATCCGCACCGCCTTCGGTGCCCACGCCGAGGTCTACGGCATGGTCGGCTACGAAGACTACGCCAAGAAGCACGGCGTCGACATCGACGGCCAGTGGTACGGCCGCCTGGGTGGTCAGGTCAAGCTGAACCAGAACTGGGGCCTGAACGGCGAGCTGAAGATGAACCGCCACGGCGACAAGGAATACACCGTCGGCCCGCGCTTCAGCTGGTAA

**Supplementary Videos 1 and 2. Time-lapse videos of *mal* upregulation**

Time-lapse videos submitted as separate video files available online.

**Video 1. Upregulation of *mal* in response to norfloxacin exposure.** Time-lapse video (3 minutes) showing the upregulation of *mal* in response to excessive norfloxacin (10 μg/ml). The arrow shows a cell with polar foci appearing, corresponding to the localisation of LysSM.

Scale bar – 5 μm.

**Video 2. Upregulation of *mal* in response to norfloxacin exposure.** Time-lapse video (3 minutes) showing the upregulation of *mal* in response to excessive norfloxacin (10 μg/ml). Multiple cells in this field of view show polar foci appearing, corresponding to the localisation of LysSM, and the white arrow shows one example.

Scale bar – 5 μm.
